# The role of selected nutraceuticals in management of prediabetes and diabetes: An updated review of the literature

**DOI:** 10.1002/ptr.7564

**Published:** 2022-08-01

**Authors:** Giuseppe Derosa, Angela D'Angelo, Pamela Maffioli

**Affiliations:** ^1^ Department of Internal Medicine and Therapeutics University of Pavia Pavia Italy; ^2^ Centre of Diabetes, Metabolic Diseases, and Dyslipidemias University of Pavia Pavia Italy; ^3^ Regional Centre for Prevention, Surveillance, Diagnosis and Treatment of Dyslipidemias and Atherosclerosis Fondazione IRCCS Policlinico San Matteo Pavia Italy; ^4^ Italian Nutraceutical Society (SINut) Bologna Italy; ^5^ Laboratory of Molecular Medicine University of Pavia Pavia Italy

**Keywords:** dysglycemia, IFG, IGT, nutraceuticals, pre‐diabetes

## Abstract

Dysglycemia is a disease state preceding the onset of diabetes and includes impaired fasting glycemia and impaired glucose tolerance. This review aimed to collect and analyze the literature reporting the results of clinical trials evaluating the effects of selected nutraceuticals on glycemia in humans. The results of the analyzed trials, generally, showed the positive effects of the nutraceuticals studied alone or in association with other supplements on fasting plasma glucose and post‐prandial plasma glucose as primary outcomes, and their efficacy in improving insulin resistance as a secondary outcome. Some evidences, obtained from clinical trials, suggest a role for some nutraceuticals, and in particular *Berberis*, *Banaba*, *Curcumin*, and *Guar gum*, in the management of prediabetes and diabetes. However, contradictory results were found on the hypoglycemic effects of *Morus*, *Ilex paraguariensis*, *Omega‐3*, *Allium cepa*, and *Trigonella faenum graecum,* whereby rigorous long‐term clinical trials are needed to confirm these data. More studies are also needed for *Eugenia jambolana*, as well as for *Ascophyllum nodosum* and *Fucus vesiculosus* which glucose‐lowering effects were observed when administered in combination, but not alone. Further trials are also needed for quercetin.

**Abbreviations**
FPG: fasting plasma glucose
FPI: fasting plasma insulin
HOMA‐B: homeostatic model assessment of β‐cell function
HOMA‐IR: homeostasis model assessment of insulin resistance
HOMA‐IS: homeostatic model assessment of insulin sensitivity
IFG: impaired fasting glucose
IGT: impaired carbohydrate tolerance
n‐3 PUFAs: omega‐3 polyunsaturated fatty acids
OGTT: oral glucose tolerance test
PPG: post‐prandial plasma glucose
PPI: post‐prandial insulin
QUICKI: quantitative insulin sensitivity check index
T2DM: type 2 diabetes mellitus
TGR: total glucose requirement

## 1 INTRODUCTION

Dysglycemia is a significant risk factor for diabetes and contributes to the development of cardiovascular disease. The term dysglycemia refers to two conditions: impaired fasting glucose (IFG) and impaired glucose tolerance (IGT), which can both evolve into type 2 diabetes mellitus (T2DM) (Gerstein, 2010).

Dysglycemia needs to be classified so that the correct therapeutic intervention can be applied: lifestyle modification, possibly with the administration of nutraceuticals in the case of IFG and IGT, or behavioral intervention combined with pharmacological treatment in the case of T2DM. Dysglycemia is defined as a fasting plasma glucose (FPG) level ≥100 mg/dl (American Diabetes Association, 2021). T2DM is diagnosed when two FPG readings are ≥126 mg/dl or when an occasional blood glucose level is ≥200 mg/dl, together with polyuria, polydipsia, polyphagia, and weight loss in the previous months.

If FPG levels are ≥100 mg/dl, but <126 mg/dl, an oral glucose tolerance test (OGTT) should be performed with 75 g of glucose and with the determination of blood glucose at time 0 (baseline) and after 120 min.

Based on blood glucose levels 2 hr after glucose loading, the patient will be diagnosed with

- IFG if the plasma glucose level after 2 hr is <140 mg/dl.
- IGT if the plasma glucose level after 2 hr is 140–199 mg/dl.
- Diabetes mellitus if the plasma glucose level after 2 hr is ≥200 mg/dl.

An OGTT is normally performed if T2DM is suspected, as type 1 diabetes mellitus is usually characterized by blood sugar levels ≥200 mg/dl, so OGTT is not needed.

In all cases, the first intervention is lifestyle modification with the patient following a healthy diet to reduce overweight or obesity, achieve adequate glycemic control, and prevent complications. Daily nutrition should be based on the Mediterranean diet, and consist of <30% fat, <10% saturated fat, >15 g insoluble and soluble fiber per 1,000 kcal, 45–60% carbohydrates, and 15–20% protein. Patients should limit their saturated fat intake to <7% of daily calorie needs; monounsaturated fatty acids, such as olive oil and other vegetable oils, however, are recommended (American Diabetes Association, 2021). The patient is also encouraged to increase their physical activity and to engage in a minimum of 30–40 min of aerobic activity at least three or four times a week. The patient should also be advised to stop smoking and consuming alcohol.

However, as it is difficult to maintain a healthy lifestyle, especially if the individual has a sedentary job, nutraceuticals have recently been marketed to help patients. Nutraceuticals include food or part of food that provides medical or health benefits including the prevention or treatment of a disease. Bioactive compounds with medicinal value isolated from food/edible materials are accepted as nutraceutical compounds. Other than food/edible materials, medicinal plants which are generally considered safe without toxicity are also considered nutraceuticals by many, but not by all.

This review aimed to evaluate the effects of the most studied nutraceuticals on glycemia in humans, evaluating their efficacy and safety.

## 2 MATERIALS AND METHODS

A systematic search strategy was developed to identify randomized controlled trials in both MEDLINE (National Library of Medicine, Bethesda, MD; 1996 through July 2021) and the Cochrane Register of Controlled Trials (The Cochrane Collaboration, Oxford, United Kingdom). The terms “nutraceutical,” “botanicals,” “dysglycemia,” “berberine,” “banaba,” “curcumin,” “guar gum,” “omega‐3 polyunsaturated fatty acids,” “psyllium,” “quercetin,” “stevia rebaudiana,” “gymnemic acid,” “ginseng,” “naringenin,” “trigonella faenum graecum,” “syzygium cumini,” “ascophyllum nodosum,” “fucus vesiculosus,” “ilex paraguariensis,” and “morus alba” were incorporated into an electronic search strategy that included the Dickersin filter for randomized controlled trials (Dickersin, Scherer, & Lefebvre, 1994). The bibliographies of all identified randomized trials and review articles were reviewed to look for additional studies of interest. We reviewed all the citations retrieved from the electronic search to identify potentially relevant articles for this review. We subsequently reviewed the potential trials to determine their eligibility. To qualify for inclusion, clinical trials were required to meet a series of predetermined criteria regarding study design, study population, interventions evaluated, and outcome measure. Eligible trials had to present results on glycemic control and adverse events. The outcome of primary interest was the mean amount decrease (in mg/dl or mmol/L) of FPG and post‐prandial plasma glucose (PPG). Variation of insulin resistance, measured using homeostasis model assessment of insulin resistance (HOMA‐IR), that occurred during various trials was a secondary outcome of interest, as was the frequency of patients having one or more adverse events. The following data were abstracted onto standardized case report forms: authors; year of publication; country of study; source of funding; study goal; means of randomization and blinding; duration of treatment; treatment characteristics; sex; quantity of and reasons for study withdrawal; age characteristics of the treatment and control groups; outcomes; and adverse event data. A validated, 3‐item scale was used to evaluate the overall reporting quality of the trials selected for inclusion in the present review. This scale provided scoring for randomization (0–2 points), double blinding (0–2 points), and account for withdrawals (1 point). Scores ranged between 0 and 5, and scores 3 indicated a study of high quality (Jadad et al., 1996) and study selection was restricted to randomized controlled trials to ensure the inclusion of only high‐quality evidence.

## 3 NUTRACEUTICALS

### 3.1 Berberis

Berberine is an isoquinoline alkaloid utilized in the folk medicine of China to treat mainly gastrointestinal infections. More recently, thanks to the hypoglycemic properties of berberine, it has been thought about its use in the treatment of diabetes. This alkaloid was isolated from many plant families including Annonaceae (*Annickia*, *Rollinia*, and *Xylopia*), Berberidaceae (*Berberis*, *Caulophyllum*, *Jeffersonia*, *Mahonia*, *Nandina*, and *Sinopodophyllum*), Menispermaceae (*Tinospora*), Papaveraceae (*Argemone*, *Bocconia*, *Chelidonium*, *Corydalis*, *Eschscholzia*, *Glaucium*, *Hunnemannia*, *Macleaya*, *Papaver*, and *Sanguinaria*), Ranunculaceae (*Coptis*, *Hydrastis*, and *Xanthorhiza*), and Rutaceae (*Evodia*, *Phellodendron*, and *Zanthoxyllum*).

It is known that, among these medicinal plants, the genus *Berberis* is the natural source of berberine most largely distributed. Although this substance was widely found in the bark, roots, and stems of plants, the parts that resulted to have the largest concentration of berberine were the bark and roots. In addition, *Coptis rhizoma* and barberry have the highest content of berberine which is between 5.2 and 7.7% (Neag et al., 2018; Yin, Ye, & Jia, 2012). As regard the chemical forms, berberine hydrochloride is the one most common that has been used in many clinical studies (Affuso et al., 2012; Derosa, Maffioli, & Cicero, 2012; Yin, Xing, & Ye, 2008; Y. Zhang et al., 2008; H. Zhang et al., 2010).

#### 3.1.1 Mechanisms of action

**FIGURE 1:**
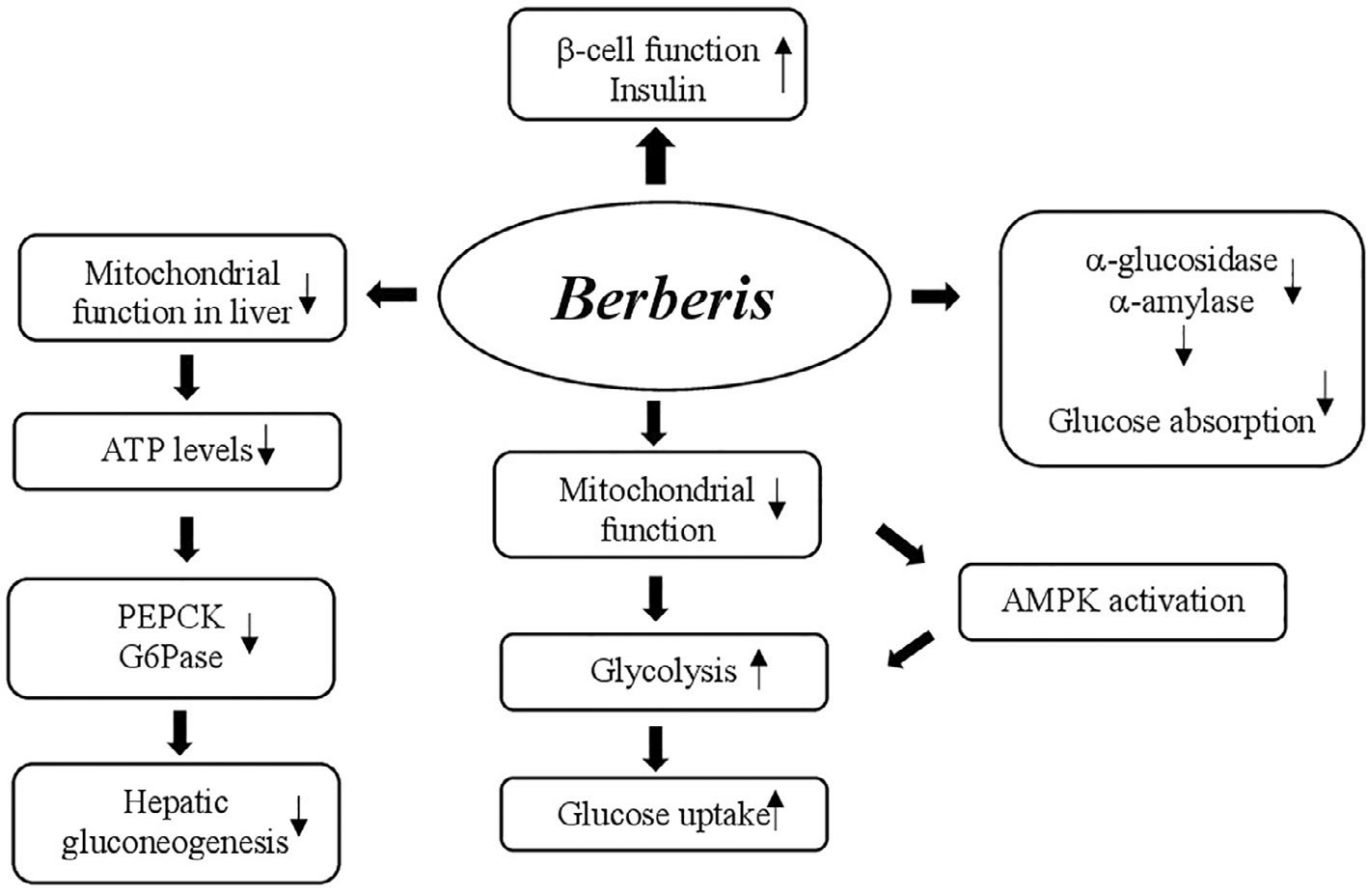
Proposed mechanisms of action of *Berberis* in glucose metabolism regulation. AMPK, adenosine monophosphate‐activated protein kinase; G6Pase, glucose‐6‐phosphatase; PEPCK, phosphoenolpyruvate carboxykinase

Different mechanisms may be involved in glucose metabolism regulation by berberine. They include (a) the increase of insulin sensitivity and improvement of pancreatic β‐cell function by repairing the exhausted islets; (b) the decrease of intestinal glucose absorption by inhibition of α‐amylase and α‐glucosidase activity; (c) the stimulation of glucose uptake through induction of glycolysis caused by inhibition of mitochondrial function. The latter also leads to adenosine monophospate‐activated protein kinase (AMPK) pathway activation; and (d) the inhibition of hepatic gluconeogenesis due to the decrease of gluconeogenic gene expression (phosphoenolpyruvate carboxykinase [PEPCK] and glucose‐6‐phosphatase [G6Pase]). This reduction has been generated by ATP depletion as a result of mitochondrial function suppression in the liver (Figure 1) (Xia et al., 2011; Xiao‐Ping, Chun‐Qing, Ping, & Ren‐Gang, 2010; Yin et al., 2012; Yin, Zhang, & Ye, 2008).

#### 3.1.2 Bioavailability

An important limit in berberine therapeutic use is its low oral bioavailability (W. Chen et al., 2011) that, in humans, is probably caused by an intestinal extrusion process P‐glycoprotein‐mediated (Pan, Wang, Liu, Fawcett, & Xie, 2002) and considerable excretion in bile (Tsai & Tsai, 2004). Since P‐glycoprotein appears to reduce the intestinal absorption of berberine by about 90% (Chae et al., 2008) its inhibition could ameliorate the oral poor bioavailability of this alkaloid. For this purpose, berberine is administered in association with nutraceuticals able to modulate P‐glycoprotein activity, such as silymarine from *Silybum marianum* which resulted in one of the best candidates both for its very low oral bioavailability and good safety profile (Zhou, Lim, & Chowbay, 2004). Other strategies such as penetration enhancers and lipid particle delivery systems have been also developed to increase berberine bioavailability (C. S. Liu, Zheng, Zhang, & Long, 2016).

#### 3.1.3 Clinical trials

In 1988, for the first time, it has been reported that the administration of berberine to treat diarrhea in 60 non‐insulin‐dependent T2DM patients reduced blood glucose levels (Ni, 1988). Since then, several trials investigated the hypoglycemic action of berberine (Table 1).

**TABLE 1:** Summary of clinical trials on the hypoglycemic effect of *Berberis*

Subjects	Drug treatment and period	Results	References
T2DM patients (*n* = 36) T2DM poorly controlled patients (*n* = 48)	1.5 g/day berberine or metformin 1.5 g/day berberine plus hypoglycemic agents 3 months	FPG, PPG, and HbA_1c_ decrease FPG, PPG, HbA_1c_, FPI, and HOMA‐IR decrease	Yin, Xing, and Ye (2008)
T2DM and dyslipidemia patients (*n* = 116)	1.0 g/day 3 months	FPG, PPG, and HbA_1c_ decrease	Y. Zhang et al. (2008)
T2DM patients (*n* = 97) T2DM or IFG with chronic hepatitis B and C patients (*n* = 35)	1.0 g/day berberine or 1.5 g/day metformin or 4 mg/day rosiglitazone Berberine 1 g/day 2 months	FPG and HbA_1c_ decrease; Insulin levels decrease; Percentage of peripheral blood lymphocytes increase FPG and liver enzymes decrease	H. Zhang et al. (2010)
T2DM and dyslipidemia patients (*n* = 60)	1.0 g/day 3 months	FPG, PPG, and HbA_1c_ decrease	Gu et al. (2010)
T2DM patients (*n* = 22)	1 g/day berberine + 210 mg/day silymarin 3 months	HBA_1c_ and FPI decrease	Di Pierro et al. (2012)
T2DM patients (*n* = 69)	1 g/day berberine + 210 mg/day silymarin or 1 g/day berberine 4 months	FPG and HbA_1c_ decrease	Di Pierro et al. (2013)
Dyslipidemia patients (*n* = 102)	1 g/day berberine + 210 mg/day silymarin 14 months	FPG and C‐peptide after 6 min glucagon test increase; FPG lower increase and C‐peptide higher increase during glucagon test	Derosa et al. (2013a)
Obesity and dyslipidemia patients (*n* = 105)	1 g/day berberine + 210 mg/day silymarin 14 months	FPI and HOMA‐IR decrease	Derosa et al. (2013b)
Dyslipidemia patients (*n* = 137)	1 g/day berberine + 210 mg/day silymarin 6 months	FPG, FPI and HOMA index decrease	Derosa, Romano, D'Angelo, and Maffioli (2015)
T2DM and hypercholesterolemia patients (*n* = 45)	1 g/day berberine + 210 mg/day silymarin or 1 g/day berberine + 210 mg/day silymarin with low dose statins or 1 g/day berberine + 210 mg/day silymarin with ezetimibe 12 months	FPG and HbA_1c_ decrease	Di Pierro, Bellone, Rapacioli, and Putignano (2015)
Type 1 diabetes mellitus patients (*n* = 85)	1 g/day berberine + 210 mg/day silymarin 6 months	FPG, PPG and HbA_1c_ decrease; Insulin consumption decrease	Derosa, D'Angelo, and Maffioli (2016)
Dyslipidemia patients (*n* = 226)	500 mg/day berberine + 105 mg/day silymarin + 10 mg/day monacolins K and KA 6 months	HbA_1c_ and HOMA‐IR decrease	Di Pierro et al. (2016)
Low cardiovascular risk patients (*n* = 143)	500 mg/day berberine + 105 mg/day silymarin + 10 mg/day monacolins K and KA 3 months	FPG and HOMA index decrease; FPI increase	Derosa, D'Angelo, Romano, and Maffioli (2017)
T2DM and dyslipidemia patients (*n* = 59)	500 mg/day berberine + 105 mg/day silymarin + 10 mg/day monacolins K and KA 6 months	HbA_1c_ decrease	Di Pierro, Putignano, and Villanova (2018)
Metabolic syndrome patients (*n* = 64)	500 mg/day berberine + 10 mg/day policosanol + 3 mg/day monacolin K 18 weeks	FPG, FPI, PPI, and HOMA‐IR decrease	Affuso et al. (2012)
IFG patients (*n* = 40)	310 mg/day berberine + 500 mg/day *lagerstroemia speciose* + 250 mg/day curcumin + 2.6 mg/day chromium picolinate + 0.30 mg/day folic acid + 220 mg/day α‐lipoic acid 8 weeks	FPG, FPI, and HOMA index decrease	Cicero et al. (2017)
IFG and IGT patients (*n* = 148)	200 mg/day berberine + 200 mg/day curcumin + 300 mg/day inositol + 40 mg/day banaba + 100 mg/day chromium picolinate 3 months	FPG, PPG, HbA_1c_, and HOMA‐IR decrease; FPI increase	Derosa, D'Angelo, Vanelli, and Maffioli (2020)

Abbreviations: FPG, fasting plasma glucose; FPI, fasting plasma insulin; HbA_1c_, glycated hemoglobin; HOMA‐IR, homeostatic model assessment of insulin resistance; IFG, impaired fasting glucose; IGT, impaired glucose tolerance; PPG, post‐prandial plasma glucose; PPI, post‐prandial plasma insulin; T2DM, type 2 diabetes mellitus.

Yin, Xing, and Ye (2008) and Yin, Zhang, and Ye (2008) evaluated the effect of berberine or metformin administration, 1.5 g/day, for 3 months, in 36 newly diagnosed T2DM subjects. It has been shown that the supplement produced a significant decrease in FPG (−66.67 mg/dl, −34.90%, *p* < .01), PPG (−156.76 mg/dl, −43.90%, *p* < .01), and glycated hemoglobin (HbA_1c_) levels (−2.00%, −27.50%, *p* < .01) compared to baseline. It has been also observed that the glucose‐lowering action of berberine was similar to that of metformin. Moreover, the authors investigated the efficacy of berberine 1.5 g/day, in addition to conventional hypoglycemic agents (sulfonylureas, metformin, acarbose, or insulin) for 3 months in 48 poorly controlled diabetics. Berberine was found to significantly reduced FPG (−36.03 mg/dl, −20.80%, *p* < .001), PPG (−91.90 mg/dl, −34.50%, *p* < .001), and HbA_1c_ values (−0.80%, −13.80%, *p* < .001) respect to baseline. The supplement also lowered fasting plasma insulin (FPI) levels (−9.90 μU/ml, −28.10%, *p* < .01) and HOMA‐IR (−6.80, −44.70%, *p* < .001) by comparison to baseline values (Yin, Xing, & Ye, 2008). The improvement of HbA_1c_ by berberine reported in the study of Yin, Xing, and Ye (2008) and Yin, Zhang, and Ye (2008) was comparable to that of metformin (DeFronzo & Goodman, 1995).

Another trial reported that berberine assumption at the dose of 1 g/day for a period of 3 months in 116 newly diagnosed T2DM patients with dyslipidemia decreased FPG, PPG, and HbA_1c_ concentrations compared to values before treatment (−25.23 mg/dl, −20.00%, *p* < .0001 for FPG; −55.86 mg/dl, −25.80%, *p* < .0001 for PPG; −0.90%, −12.00%, *p* < .0001 for HbA_1c_) and to placebo (−14.42 mg/dl, −12.50%, *p* < .0001 for FPG; −37.84 mg/dl, −19.10%, *p* < .0001 for PPG; −0.70%, −9.60%, *p* < .0001 for HbA_1c_). Regarding HOMA‐IR, FPI, and post‐prandial insulin (PPI) values no significant differences were found between berberine and placebo (Y. Zhang et al., 2008). The lowering of HbA_1c_ obtained with berberine in this study was like that achieved with conventional glucose‐lowering oral agents (Bloomgarden, Dodis, Viscoli, Holmboe, & Inzucchi, 2006).

In the trial by H. Zhang et al. (2010), 97 patients with T2DM were enrolled and divided into 3 groups treated with 1 g/day of berberine, 1.5 g/day of metformin, and 4 mg/day of rosiglitazone, respectively, for 2 months. Berberine therapy significantly decreased FPG (−48.65 mg/dl, −26.00%, *p* < .001) and HbA_1c_ levels (−1.50%, −23.90%, *p* < .001) compared to baseline, and the lowering of these glycemic parameters was similar to that obtained with metformin (−59.46 mg/dl, −30.30%, *p* < .001 for FPG; −2.20%, −30.40%, *p* < .001 for HbA_1c_) and rosiglitazone (−28.82 mg/dl, −17.60%, *p* < .01 for FPG; −1.50%, −23.90%, *p* < .001 for HbA_1c_). In addition, berberine supplementation reduced insulin levels (−28.2%, *p* < .01) and increased the percentage of peripheral blood lymphocytes that express insulin receptors (*p* < .01). The authors also reported the hypoglycemic effect of berberine administration at the dose of 1 g/day for 2 months in 35 chronic hepatitis B and hepatitis C subjects with T2DM or IFG. In both hepatitis B and C groups, the supplement decreased FPG levels in T2DM (−19.82 mg/dl, −15.10%, *p* < .01 for hepatitis B group; −23.40 mg/dl, −17.10%, *p* < .01 for hepatitis C group), and IFG patients (−16.22 mg/dl, −14.10%, *p* < .01 for hepatitis B group; −16.22 mg/dl, −13.40%, *p* < .01 for hepatitis C group) respect to baseline. Moreover, these patients showed a significant lowering of liver enzymes after berberine treatment (H. Zhang et al., 2010).

Gu et al. (2010) showed that 1 g/day of berberine improved the glycemic parameters in 60 subjects with newly diagnosed T2DM and dyslipidemia. After 3 months of treatment, it has been observed a significant reduction of FPG, PPG, and HbA_1c_ levels in the berberine group by comparison with baseline (−25.22 mg/dl, −20.30%, *p* < .001 for FPG; −46.85 mg/dl, −22.80%, *p* < .001 for PPG; −0.90%, −11.80%, *p* < .001 for HbA_1c_) and placebo (−14.41 mg/dl, −12.70%, *p* = .008 for FPG; −48.65 mg/dl, −23.50%, *p* = .001 for PPG; −0.70%, −9.50%, *p* = .004 for HbA_1c_) (Gu et al., 2010).

Some clinical trials demonstrated the hypoglycemic action of berberine in association with other nutraceuticals.

Di Pierro et al. (2012) tested a nutraceutical association, in tablet form, composed of 588 mg of *Berberis aristata* extract (titrated as 85% berberine) corresponding to 500 mg of berberine and 105 mg of *S. marianum* extract (titrated as >60% silymarin) in 22 T2DM patients with suboptimal glycemic control and on therapy with traditional hypoglycemic drugs. The supplement, dosed at 1 g of berberine and 210 mg of silymarin daily for 3 months, determined a significant lowering of HbA_1c_ (−0.85%, −14.10%, *p* = .003) and FPI levels (−7.20 μU/ml, −31.70%, *p* = .04), together with HOMA‐IR values (−1.80, −26.10%, *p* = .04) compared to baseline (Di Pierro et al., 2012).

The same authors investigated the effect of *B. aristata* and *S. marianum* combination versus *B. aristata* alone in 69 T2DM patients who had suboptimal glycemic control and who were treated with diet and conventional glycemic‐lowering therapy. These patients received 1 g/day of berberine with 210 mg/day of silymarin or 1 g/day of only berberine for 4 months as an adjunct therapy. Both treatments similarly lowered FPG (−28.39 mg/dl, −18.00%, *p* = .006 for berberine and silymarin combination; 27.12 mg/dl, −17.10%, *p* = .007 for berberine alone) and HbA_1c_ levels (−0.56%, −7.20%, *p* < .001 for berberine and silymarin combination; −0.99%, −12.30%, *p* < .001 for berberine alone) respect to baseline. However, the nutraceutical combination produced a higher reduction in HbA_1c_ values (−0.22%, −3.00%, *p* < .05) as compared to berberine alone (Di Pierro et al., 2013).

In a 14‐month clinical trial, it has been evaluated the effect of *B. aristata* and *S. marianum* combination on insulin secretion in a sample of 102 euglycemic dyslipidemic subjects. At baseline, they followed a controlled‐energy diet and made exercised for 6 months. Subsequently, they were randomized to receive 1 g/day of berberine with 210 mg/day of silymarin or placebo for 3 months and after an interruption of 2 months they took again the supplement or placebo for other 3 months. In the nutraceutical group, during glucagon stimulation test, FPG and C‐peptide levels were increased after 6 min (+26.60 mg/dl, +32.10%, *p* < .05 for FPG; +4.65 ng/ml, +97.70%, *p* < .05 for C‐peptide) versus time 0 as well as the values of the latter at time 0 were found to be higher respect to those at time 0 at baseline (+1.50 ng/ml, +46.00%, *p* < .05), at randomization (+1.69 ng/ml, +55.00%, *p* < .05), and at the end of study in placebo group (+1.62 ng/ml, +51.60%, *p* < .05). It has been also observed that the supplement had increased to a lesser extent FPG concentrations after 6 min from the glucagon injection compared to those at 6 min at baseline (−14.30 mg/dl, −11.60%, *p* < .05) at randomization (−8.80 mg/dl, −7.40%, *p* < .05) and at the end of the study in the placebo group (−16.80 mg/dl, −13.30%, *p* < .05), whereas had raised to a greater extent C‐peptide levels at 6 min respect to those at 6 min at baseline (+0.57 ng/dl, +6.40%, *p* < .05) at randomization (+1.10 ng/dl, +13.20%, *p* < .05) and at the end of the study in the placebo group (+0.88 ng/dl, +10.30%, *p* < .05) (Derosa et al., 2013a).

The same authors tested the efficacy of *B. aristata* and *S. marianum* association in another study whose design was identical to that described above and in which were enrolled 105 euglycemic, overweight, and dyslipidemic patients. The nutraceutical combination improved insulin resistance by reducing FPI levels and HOMA‐IR. In detail, these parameters were lowered at 3 months from randomization both to baseline (−1.80 μU/ml, −17.80%, *p* < .05 for FPI; −0.40, −19.00%, *p* < .05 for HOMA‐IR) and placebo (−1.10 μU/ml, −11.70%, *p* < .05 for FPI; −0.23, −11.90%, *p* < .05 for HOMA‐IR). The interruption of supplementation produced an increase in FPI levels (+1.60 μU/ml, +19.30%, *p* < .05) and HOMA‐IR (+0.35, +20.60%, *p* < .05) respect to 3 months from randomization while its reintroduction lowered again these parameters compared to wash‐out (−1.60 μU/ml, −16.20%, *p* < .05 for FPI; −0.34, −16.60%, *p* < .05 for HOMA‐IR) and placebo (−1.50 μU/ml, −15.30%, *p* < .05 for FPI; −0.30, −14.90%, *p* < .05 for HOMA‐IR) (Derosa et al., 2013b).

In one trial, a total of 137 euglycemic, dyslipidemic subjects taking half dose of statin being intolerant to these drugs at high dosages, received a placebo or *B. aristata* combined with *S. marianum* at the dose of 1 g/day of berberine with 210 mg/day of silymarin for 6 months. At the end of treatment, the supplement significantly reduced FPG, FPI, and HOMA‐IR respect to baseline (−9.00 mg/dl, −9.70%, *p* < .05 for FPG; −0.70 μU/ml, −7.90%, *p* < .05 for FPI; −0.35, −17.00%, *p* < .05 for HOMA‐IR) and placebo (−6.40 mg/dl, −7.10%, *p* < .05 for FPG; −1.00 μU/ml, −10.90%, *p* < .05 for FPI; −0.33, −16.20%, *p* < .05 for HOMA‐IR) (Derosa et al., 2015).

The beneficial effect of *B. aristata* and *S. marianum* association on glycemic profile has been also demonstrated in 45 patients with T2DM, hypercholesterolemia, and intolerance to statins. In this trial, the subjects were divided into 3 groups who received the supplement (1 g/day of berberine with 210 mg/day of silymarin) alone and together with low dose statins or ezetimibe, respectively. It has been observed that the nutraceutical combination significantly reduced FPG levels after 6 months of treatment only when administered as monotherapy (−13.10 mg/dl, −9.30%, *p* < .05), while a significant lowering was found in all groups after 12 months (−14.40 mg/dl, −10.20%, *p* < .05 for supplement alone; −14.50 mg/dl, −10.40%, *p* < .05 for supplement plus statins; −13.10 mg/dl, −8.90%, *p* < .05 for supplement plus ezetimibe) respect to baseline values. The groups treated with the nutraceutical association alone and in addition to statins showed a significant decrease in FPG values (−13.60 mg/dl, −9.60%, *p* < .05 for supplement alone; −13.80 mg/dl, −9.70%, *p* < .05 for supplement plus statins) as compared to that who received the nutraceutical combination together with ezetimibe, after 6 months of therapy. It has been also reported a significant reduction of HbA_1c_ levels after 6 months (−0.40%, −5.30%, *p* < .05 for supplement alone; −0.20%, −2.60%, *p* < .05 for supplement plus statins; −0.20%, −2.60%, *p* < .05 for supplement plus ezetimibe) and at the end of treatment (−0.40%, −5.30%, *p* < .05 for supplement alone; −0.40%, −5.20%, *p* < .01 for supplement plus statins; −0.20%, −2.60%, *p* < .05 for supplement plus ezetimibe) in all groups respect to baseline (Di Pierro et al., 2015).

Derosa, D'Angelo, and Maffioli (2016) showed the role of *B. aristata* and *S. marianum* combination in 85 patients with type 1 diabetes mellitus treated with insulin for at least 3 months and subjected to a hypocaloric diet together with increased physical activity. The administration of the nutraceutical at the dose of 1 g/day of berberine with 210 mg/day of silymarin for 6 months as add‐on therapy resulted in a significant lowering of FPG and PPG concentrations both to baseline (−17.20 mg/dl, −11.60%, *p* < .05 for FPG; −23.30 mg/dl, −12.00%, *p* < .05 for PPG) and to placebo (−14.20 mg/dl, −9.80%, *p* < .05 for FPG; −14.20 mg/dl, −7.70%, *p* < .05 for PPG) unlike HbA_1c_ levels that were reduced only by comparison to values before the supplementation (−0.40%, −5.00%, *p* < .05). The nutraceutical also decreased total insulin consumption (−0.42 IU, −14.70%, *p* < .05 vs. baseline, and −0.42 IU, −14.50%, *p* < .05 vs. placebo) and that at meals (for breakfast: −75.00 IU, −13.80%, *p* < .05 vs. baseline, and −59.00 IU, −11.20%, *p* < .05 vs. placebo; for lunch: −150.00 IU, −26.90%, *p* < .01 vs. baseline, and −153.00 IU, −27.30%, *p* < .01 vs. placebo; for dinner: −96.00 IU, −15.0%, *p* < .01 vs. baseline, and −106.00 IU, −16.30%, *p* < .01 vs. placebo), as well as that at bedtime (−150.00 IU, −14.00%, *p* < .05 vs. baseline, and −143.00 IU, −13.50%, *p* < .05 vs. placebo) (Derosa, D'Angelo, & Maffioli, 2016).

In a retrospective study, it has been evaluated the effect of a supplement, in form of a tablet, containing 500 mg of berberine from *B. aristata*, 105 mg of silymarin from *S. marianum*, and 10 mg of monacolins K and KA from *Monascus purpureus* fermented rice in 226 dyslipidemic patients that were naïve and intolerant to statins. After 6 months of treatment, in the group that received the supplement alone it has been observed a significant reduction of HbA_1c_ (−0.64%, −10.90%, *p* < .05) and HOMA‐IR values (−0.45, −16.70%, *p* < .05) respect to baseline. In addition, the group that consumed the nutraceutical as adjunct therapy presented a reduction of HbA_1c_ (−0.47%, −8.40%, *p* < .05) and HOMA‐IR (−0.38, −14.10%, *p* < .05) slightly lower than that obtained from supplement alone, by comparison to baseline values (Di Pierro et al., 2016).

One trial also demonstrated that the administration of the same supplement association for 3 months in 143 subjects with low cardiovascular risk determined a significant lowering of FPG (−11.50 mg/dl, −12.20%, *p* < .05) with respect to placebo and an increase of FPI levels both to baseline (+0.70 μU/ml, +7.40%, *p* < .05) and placebo (+1.00 μU/ml, +9.90%, *p* < .05). Moreover, HOMA‐IR was decreased after nutraceutical treatment (−0.06, −2.85%, *p* < .05) compared to placebo (Derosa et al., 2017).

Furthermore, the same nutraceuticals combination was examined in a retrospective analysis including 59 T2DM patients with dyslipidemia subjected to hypoglycemic treatments consisting of hypocaloric and low glycemic index diet, physical activity, and hypoglycemic agents. Of the 59 patients analyzed, only 31 were treated with one tablet of supplement per day for 6 months, whereas 28 were used as controls. At the end of therapy, the nutraceutical treated group showed a significant decrease in HbA_1c_ levels (−0.35%, −5.20%, *p* < .05) as compared to those before the supplementation (Di Pierro et al., 2018).

Based on these data the authors state that the effect of *B. aristata* and *S. marianum* combination on glycemic parameters is likely attributable to the presence of berberine.

One short‐term study investigated the effect of a nutraceuticals combination as tablet form each containing berberine 500 mg, policosanol 10 mg, and red yeast rice (monacolin K 3 mg) administered to 64 subjects with metabolic syndrome, one tablet daily, for 18 weeks. At the end of treatment, the supplement group showed a significant decrease in FPG (−7.92 mg/dl, −7.70%, *p* = .006) respect to baseline and FPI levels (−5.04 μU/ml, −31.50%, *p* = .042), as well as mean PPI (−30.09 μU/ml, −41.20%, *p* = .023) by comparison to placebo. It has been also found a reduction of HOMA‐IR both to baseline (−0.60, −18.80%, *p* = .019) and to placebo (−0.60, −18.80%, *p* = .023) in the group that received the nutraceuticals combination (Affuso et al., 2012).

A short‐term trial evaluated the hypoglycemic activity of berberine 155 mg combined with *Lagerstroemia speciose* extract 250 mg, curcumin extract 125, α‐lipoic acid 110 mg, chromium picolinate 1.3 mg, and folic acid 0.15 mg in 40 IFG patients in primary prevention of cardiovascular disease that received the supplement or placebo as pills twice daily for 8 weeks. The patients treated with nutraceuticals association showed a significant decrease in FPG levels (−16.00 mg/dl, −14.50%, *p* < .05), as well as the placebo group (−4.80 mg/dl, −4.20%, *p* < .05) respect to baseline even if to a lesser extent than the supplement. In addition, the nutraceuticals combination significantly lowered FPI and HOMA‐IR both to baseline (−2.60 μU/ml, −13.40%, *p* < .05 for FPI; −1.28, −24.60%, *p* < .05 for HOMA‐IR) and to placebo (−1.30 μU/ml, −7.20%, *p* < .05 for FPI; −1.01, −20.50%, *p* < .05 for HOMA‐IR). However, these findings require confirmation through middle‐ and long‐term studies with a higher number of subjects (Cicero et al., 2017).

Recently it has been shown the effect of berberine associated with curcumin, inositol, banaba, and chromium picolinate in IFG or IGT patients not treated with any glucose‐lowering agents. A total of 148 subjects received one tablet daily containing berberine 200 mg, curcumin 200 mg, inositol 300 mg, banaba 40 mg, and chromium picolinate 100 mg or placebo for 3 months. At the end of treatment, the nutraceutical combination reduced FPG, PPG, and HbA_1c_ levels compared to baseline (−15.00 mg/dl, −12.80%, *p* < .05 for FPG; −9.70 mg/dl, −7.60%, *p* < .05 for PPG; −0.40%, −6.70%, *p* < .05 for HbA_1c_) and to placebo (−14.10 mg/dl, −12.10%, *p* < .05 for FPG; −12.10 mg/dl, −9.30%, *p* < .05 for PPG; −0.20%, −3.40%, *p* < .05 for HbA_1c_). It has been observed also an increase in FPI and a decrease in HOMA‐IR after taking the supplement respect to baseline values (+0.60 μU/ml, +6.70%, *p* < .05 for FPI; −0.18, −7.00%, *p* < .05 for HOMA‐IR) and to placebo (+0.60 μU/ml, +6.70%, *p* < .05 for FPI; −0.12, −4.80%, *p* < .05 for HOMA‐IR). As regards the improvement of glycemic parameters shown in this study it is probably due to a synergistic action between berberine and the other nutraceuticals (Derosa, D'Angelo, Vanelli, & Maffioli, 2020).

### 3.2 Banaba [*L. speciose* (L) Pers]

Banaba [*L. speciose* (L) Pers] is a plant that grows in tropical countries such as the Philippines, India, Malaysia, southern China, and Australia where it is utilized as a folk medicine for health benefits. In the Philippines, tea from the leaves is used for the treatment of diabetes and kidney disease. Banaba leaves are rich in corosolic acid and ellagitannins to which the hypoglycemic activity of banaba has been attributed (Judy et al., 2003; Stohs, Miller, & Kaats, 2012).

#### 3.2.1 Mechanisms of action

**FIGURE 2:**
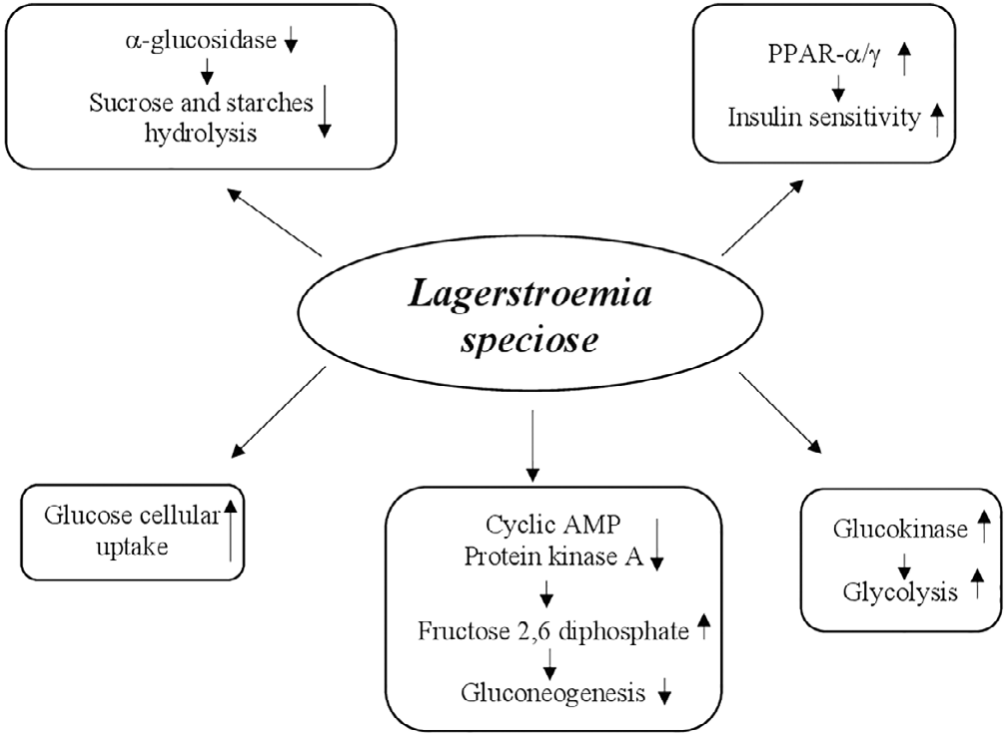
Proposed hypoglycemic mechanisms of *Lagerstroemia speciose*. *P*, adenosine monophosphate; PPAR‐α/γ, peroxisome proliferator‐activated receptor‐α/γ

Studies in vivo and in vitro showed that the glucose‐lowering effect of banaba seems due to multiple mechanisms including (a) enhancement of glucose cellular uptake, (b) reduction of sucrose and starches hydrolysis, (c) decrease of gluconeogenesis by increasing fructose 2,6 diphosphate levels through the reduction of cyclic AMP and inhibition of protein kinase A activity, (d) increase of glycolysis by raising glucokinase activity, (e) enhancement of insulin sensitivity by increasing expression of liver peroxisome proliferator‐activated receptor‐α (PPAR‐α) mRNA and adipose tissue peroxisome proliferator‐activated receptor‐γ (PPAR‐γ) mRNA, and (f) inhibition of α‐glucosidase activity (Stohs et al., 2012) (Figure 2).

#### 3.2.2 Clinical trials

The human studies in which it was analyzed the hypoglycemic action of banaba and its constituents are summarized in Table 2.

**TABLE 2:** Summary of clinical trials on glucose‐lowering activity of *Lagerstroemia speciose*

Subjects	Drug treatment and period	Results	References
FPG > 100 mg/dl patients (*n* = 15)	100 mg/day banaba extract 1 year	FPG decrease	Ikeda et al. (2002)
T2DM non‐insulin‐dependent patients (*n* = 10)	16 mg/day; 32 mg/day; 48 mg/day banaba extract standardized to 1% corosolic acid 2 weeks	FPG decrease at the highest dose	Judy et al. (2003)
FPG = 104 mg/dl patients (*n* = 12)	10 mg/day corosolic acid 2 weeks	FPG and 1 hr‐PPG decrease	Tsuchibe, Kataumi, Mori, and Mori (2006)
IFG, IGT, and T2DM patients, subjects with normal glucose tolerance (*n* = 31)	10 mg corosolic acid 5 min before OGTT Different occasions	PPG from 60 to 120 min decrease	Fukushima et al. (2006)
Prediabetes patients (*n* = 45)	300 mg/day banaba extract 2 g/day soybean leaf extract 12 weeks	FPG, HbA_1c_, and HOMA‐IR decrease	M. S. Choi et al. (2014)
T2DM patients (*n* = 24)	9 tablets/day containing banaba extract, green tea, green coffee, *garcinia*	FPG decrease	Ikeda, Chen, and Matsuda (1999)
IGT and T2DM patients (*n* = 62)	6 g/day mixture of banaba leaf extract, mulberry leaf extract, Korean red ginseng powder 6 months	Glucose AUC decrease Downward trend insulin AUC	H. J. Kim, Yoon, et al. (2012)
IFG patients (*n* = 40)	310 mg/day berberine + 500 mg/day *lagerstroemia speciose* + 250 mg/day curcumin + 2.6 mg/day chromium picolinate + 0.30 mg/day folic acid + 220 mg/day α‐lipoic acid 8 weeks	FPG, FPI, and HOMA index decrease	Cicero et al. (2017)
IFG and IGT patients (*n* = 148)	200 mg/day berberine + 200 mg/day curcumin + 300 mg/day inositol + 40 mg/day banaba + 100 mg/day chromium picolinate 3 months	FPG, PPG, HbA_1c_, and HOMA‐IR decrease; FPI increase	Derosa, D'Angelo, Vanelli, and Maffioli (2020)

Abbreviations: AUC, area under the curve; FPG, fasting plasma glucose; FPI, fasting plasma insulin; HbA_1c_, glycated hemoglobin; HOMA‐IR, homeostatic model assessment of insulin resistance; HOMA index, homeostatic model assessment index; IFG, impaired fasting glucose; IGT, impaired glucose tolerance; OGTT, oral glucose tolerance test; PPG, post‐prandial glucose; T2DM, type 2 diabetes mellitus.

A 1‐year trial performed on 15 subjects with FPG levels greater than 110 mg/dl that received 100 mg/day of a water‐soluble banaba extract in the form of tablets showed that the supplement reduced FPG levels (16.60%). Although the extract was not standardized and the components responsible for the hypoglycemic effect were not determined, the latter is mainly due to ellagitannins since the low aqueous solubility of corosolic acid (Ikeda et al., 2002).

Judy et al. (2003) evaluated the glucose‐lowering action of a banaba extract standardized to 1% corosolic acid in 10 T2DM non‐insulin‐dependent. These subjects were treated with a daily dose of 16, 32, and 48 mg of supplement in the soft gel or hard gel formulations for 2 weeks. At the end of treatment, the highest dosage of nutraceuticals determined a significant reduction of FPG in both formulations (−30.00%, *p* ≤ .002 for soft gel form; −20.20%, *p* ≤ .001 for hard gel form) respect to baseline. Since the decrease was greatest in the soft gel formulation, the latter presents a better bioavailability compared to the dry‐powder formulation. However, has not been established if corosolic acid and tannins alone or in combination are responsible for FPG reduction (Judy et al., 2003).

Another study reported that a soft gel capsule of banaba extract standardized to 18% corosolic acid containing 10 mg of the latter was daily given to 12 subjects with FPG levels of 104 mg/dl. After 2 weeks of supplementation, it has been recorded a 12% decrease in FPG and 1 hr‐PPG levels. Despite the high content of corosolic acid has not been clarified if the hypoglycemic activity is exclusively attributed to the latter or to a combination of corosolic acid and tannins (Tsuchibe et al., 2006).

In the trial by Fukushima et al. (2006) 31 subjects of which 19 with T2DM, 7 with IGT, 1 with IFG, and 4 with normal glucose tolerance took a capsule containing 10 mg of corosolic acid or placebo on different occasions for 5 min before OGTT. The interval between corosolic acid and placebo treatments was 7 days. It has been observed that the supplement reduced plasma glucose levels from 60 to 120 min and achieved statistical significance at 90 min (*p* < .05) compared to placebo. Moreover, having been used corosolic acid 99% pure, the glucose‐lowering effect was unequivocally due to the latter (Fukushima et al., 2006).

M. S. Choi et al. (2014) compared the hypoglycemic effect of banaba extract (BE) and soybean leaf extract (SLE) in 45 subjects with prediabetes that consumed 300 mg/day of BE (0.3% corosolic acid), 2 g/day of SLE or 2 g/day placebo during meals for 12 weeks. The supplementation of BE and SLE similarly decreased HbA_1c_ levels (−0.58%, −8.60%, *p* < .05 for BE; −0.59%, −8.80%, *p* < .05 for SLE) respect to placebo. Moreover, the BE and SLE groups exhibited significantly reduced levels of baseline‐adjusted final FPG (−17.70 mg/dl, −14.40%, *p* < .05 for BE; −22.07 mg/dl, −17.97%, *p* < .05 for SLE) and HOMA‐IR (−0.32, −27.10%, *p* < .05 for BE; −0.26, −22.00%, *p* < .05 for SLE) compared to placebo (M. S. Choi et al., 2014).

Some studies showed the hypoglycemic action of banaba in association with other nutraceuticals.

One trial reported the effect of a product in tablet form containing an aqueous extract of banaba, green tea, green coffee, and *Garcinia* administered at the dose of three tablets three times daily to 24 mild T2DM subjects. It has been observed an average reduction of 13.50% in FPG levels. However, the components responsible for the hypoglycemic effect were not determined (Ikeda et al., 1999).

A 6‐month study conducted on 62 IGT and mild T2DM patients that have been treated with a 6 g/day mixture of banaba leaf water extract, mulberry leaf water extract, and Korean red ginseng powder in an equivalent ratio or placebo, demonstrated that the nutraceutical combination did not affect FPG, FPI HOMA‐IR, and HbA_1c_ levels both to baseline and to placebo. However, in the treatment group, it has been observed a significant decrease in the glucose area under the curve (AUC) during OGTT (−1.96 μM/ml, −8.20%, *p* < .05) and a tendency to reduction of insulin AUC during OGTT which did not reach statistical significance respect to baseline (H. J. Kim, Yoon, et al., 2012).

Moreover, the effect of two different nutraceuticals associations containing *L. speciose*, curcumin, berberine, α‐lipoic acid, chromium picolinate, folic acid, and banaba, curcumin, berberine, inositol, chromium picolinate respectively on glucose homeostasis has been previously cited (Cicero et al., 2017; Derosa, D'Angelo, Vanelli, & Maffioli, 2020).

### 3.3 Curcumin [*Curcuma longa* (L) Pers]

Curcumin is the main and most representative bioactive substance present in the orange tuberous rhizome of *C. longa* also known as turmeric. In curcuminoids, curcumin is represented by ~75% followed by demethoxycurcumin at ~20% and bisdemethoxycurcumin at ~5% (Stanić, 2017). Curcumin may play a role in the prevention and treatment of several diseases thanks to its antioxidant, antiinflammatory, antimicrobial, hypoglycemic, and antineoplastic action. All these biological and pharmacological activities are due to the peculiar chemical structure of curcumin which is capable to have a great number of molecular targets (Pivari, Mingione, Brasacchio, & Soldati, 2019). Regarding curcumin effects the hypoglycemic one was examined.

#### 3.3.1 Mechanisms of action

**FIGURE 3:**
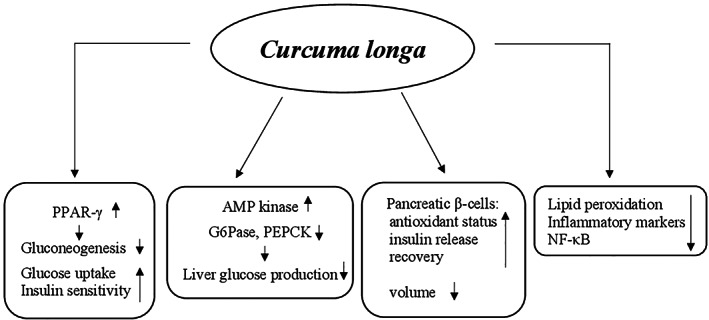
Potential hypoglycemic mechanisms of action of *Curcuma longa*. AMP, adenosine monophosphate; G6Pase, glucose‐6‐phosphatase; NF‐κB: nuclear factor kappa chain transcription in B cells; PEPCK: phosphoenolpyruvate carboxykinase; PPAR‐γ, peroxisome proliferator‐activated receptor‐γ

Many animal studies reported that different mechanisms and several molecular targets are responsible for the amelioration of the pathological events in T2DM made by curcumin. This supplement exerts the glucose‐lowering action enhancing PPAR‐γ activation, similarly to thiazolidinediones, a class of oral hypoglycemic agents. The PPAR‐γ activation determined inhibition of gluconeogenesis and increase of glucose uptake and insulin sensitivity (Nishiyama et al., 2005). Curcumin is also able to act on pancreatic β‐cells by (a) increasing their antioxidant status, (b) enhancing the release of insulin after induction of electrical activity, (c) decreasing the volume probably due to a loss of Cl^−^ and water following activation of the anion channel, and (d) improving the recovery through the induction of heme oxygenase‐1 expression together with heat‐shock protein 70 (Aggarwal & Harikumar, 2009). The hypoglycemic action of curcumin is also mediated by the reduction of lipid peroxidation and inflammatory markers such as interleukin‐6 (IL‐6), TNF‐α, and monocyte chemoattractant protein‐1 (MCP‐1) in addition to inhibition of nuclear factor kappa chain transcription in B cells (NF‐κB) signaling pathway (Jain, Rains, Croad, Larson, & Jones, 2009). Moreover, curcumin inhibits liver glucose production through activation of AMP kinase and inhibition of G6Pase as well as PEPCK activity (Fujiwara et al., 2008; Seo et al., 2008) (Figure 3).

##### Bioavailability

Bioavailability constitutes the greater problem for the clinical use of curcumin, because of its rapid metabolism, intestinal poor absorption, and rapid excretion when it is administered orally (Anand, Kunnumakkara, Newman, & Aggarwal, 2007). Some techniques have been developed to solve these problems (Neerati, Devde, & Gangi, 2014).

The early approach to enhancing absorption of curcumin was the use of adjuvants like piperine, an inhibitor of hepatic and intestinal glucuronidation. Shoba et al. (1998) had given curcumin alone and combined with piperine to rats and healthy human volunteers. In rats, when curcumin was given alone at the dose of 2 g/kg, modest serum levels were detected over a period of 4 hr, whereas the addition of piperine 20 mg/kg produced a rise in curcumin serum concentration for 1–2 hr and a decrease of elimination half‐life and clearance increasing bioavailability of 154%. In healthy volunteers, after the intake of curcumin 2 g, serum concentrations were not detectable or very low, whereas concomitant consumption of piperine 20 mg increased serum levels from 0.25 to 1 hr rising bioavailability by 2000%. Therefore, the effect of piperine on the bioavailability of curcumin was higher in humans than in rats. The inhibition of glucuronidation by piperine may be considered the major mechanism to increase the bioavailability of curcumin. Moreover, the use of piperine caused no adverse events favoring the positive effects of curcumin on various diseases.

Another approach is represented by curcumin nanoparticles which are particles with a diameter of about 1–100 nm which have been encapsulated the supplement. In general, nanoformulations can be useful for drug delivery because they can improve pharmacokinetics and solubility, provide targeted delivery, and control drug release. To date, polymer, solid lipid, magnetic, gold, and albumin‐based nanoparticles are widely used to ameliorate the use of curcumin as a therapeutic agent (Karthikeyan, Senthil, & Min, 2020). Experimental studies with curcumin nanoformulations showed highly increased absorption and a desirable blood level of the active forms of the supplement (Stohs et al., 2020). In addition, in vivo pharmacokinetic studies demonstrated that nanocurcumin increased oral bioavailability by at least ninefold compared to curcumin administered in combination with piperine. (Shaikh, Ankola, Beniwal, Singh, & Kumar, 2009). These data support curcumin nanoparticles' use in various diseases including diabetes prophylaxis, pain management, and tissue protection (Pivari et al., 2019; Stohs et al., 2020).

It has also shown that phytosome technology is one of the most promising in regard to the efficacy and tolerability of curcumin in several diseases (Appendino et al., 2011; Mirzaei et al., 2017).

#### 3.3.2 Clinical trials

Several human studies investigated the glucose‐lowering action of curcumin (Table 3).

**TABLE 3:** Summary of clinical trials on glucose metabolism *Curcuma longa* effect

Subjects	Drug treatment and period	Results	References
T2DM patients (*n* = 72)	300 mg/day curcumin 10 mg/day atorvastatin 8 weeks	No decrease in FPG and HbA_1c_	Usharani, Mateen, Naidu, Raju, and Chandra (2008)
Healthy subjects (*n* = 14)	6 g curcumin Single meal	PPI increases at 30 and 60 min after OGTT; PPI AUC increases at 15, 30, 90, and 120 min after OGTT; GII increase	Wickenberg, Ingemansson, and Hlebowicz (2010)
T2DM nephropathy patients (*n* = 40)	66.3 mg/day curcumin 2 months	No changes in FPG and 2 hr‐PPG	Khajehdehi et al. (2011)
IFG and IGT patients (*n* = 240)	250 mg/day curcumin 9 months	FPG, 2 hr‐PPG, HbA_1c_, FPI, HOMA‐IR decrease; HOMA‐B increase	Chuengsamarn, Rattanamongkolgul, Luechapudiporn, Phisalaphong, and Jirawatnotai (2012)
T2DM, overweight and obesity patients (*n* = 100)	300 mg/day curcuminoids 3 months	FPG, HbA_1c_, and HOMA‐IR decrease	Na et al. (2013)
IGT and T2DM patients (*n* = 71)	11.36 g/day *Jiangtang Xiaozhi* (1.5 g/day curcumin) 16 weeks	PPG and FPI decrease	Grant et al. (2013)
T2DM patients (*n* = 8)	475 mg/day curcumin 5 mg/day glyburide 10 days	Downward trend 2 hr‐PPG, 6 hr‐PPG, and 12 hr‐PPG	Neerati et al. (2014)
Metabolic syndrome patients (*n* = 65)	1.89 g/day curcumin extract 12 weeks	HbA_1c_ downward trend in the female subgroup	Yang et al. (2014)
T2DM patients (*n* = 240)	1.5 g/day curcumin 6 months	FPI and HOMA‐IR decrease	Chuengsamarn, Rattanamongkolgul, Phonrat, Tungtrongchitr, and Jirawatnotai (2014)
Metabolic syndrome patients (*n* = 250)	2.4 g/day curcumin 1.5 g/day black seed 900 mg/day black seed + 1.5 g/day curcumin 8 weeks	FPG decreased by black seed + curcumin	Amin, Islam, Anila, and Gilani (2015)
Metabolic syndrome, NAFLD patients (*n* = 80)	70 mg/day curcuminoids 8 weeks	FPG and HbA_1c_ decrease	Rahmani et al. (2016)
Diabetic and non‐diabetic proteinuric CKD patients (*n* = 101)	320 mg/day curcumin 8 weeks	Downward trend FPG	Jiménez‐Osorio et al. (2016)
T2DM patients (*n* = 70)	80 mg/day curcumin as nano‐micelle 3 months	FPG and HbA_1c_ decrease	Rahimi et al. (2016)
T2DM patients (*n* = 100)	500 mg/day curcuminoids + 5 mg/day piperine 3 months	FPG, HbA_1c_, FPI, HOMA‐IR and C‐peptide decrease	Panahi et al. (2018)
T2DM, hyperlipidemia patients (*n* = 80)	2.1 g/day curcumin 8 weeks	No decrease in FPG, HbA_1c_, FPI and HOMA‐IR	Adab et al. (2019)
T2DM, overweight patients (*n* = 44)	1.5 g/day curcumin 10 weeks	FPG decrease	Hodaei, Adibian, Nikpayam, Hedayati, and Sohrab (2019)
IFG, T2DM non‐insulin‐dependent patients (*n* = 33)	180 mg/day highly absorbable curcumin preparation 6 months	No significant FPG decrease	Funamoto et al. (2019)
T2DM, dyslipidemia patients (*n* = 32)	1.2 g/day curcumin + garlic; 1.6 g/day curcumin + garlic; 2.4 g/day curcumin + garlic 12 weeks	2 hr‐PPG and HbA_1c_ decreased at the highest dose	Sukandar et al. (2010)
T2DM, with or without dyslipidemia patients (*n* = 29)	2.4 g/day curcumin + garlic 5 mg/day glibenclamide 14 weeks	FPG, 2 hr‐PPG, and HbA_1c_ decrease	Sukandar, Sudjana, Adnyana, Setiawan, and Yuniarni (2014)
Hypercholesterolemia patients (*n* = 80)	250 mg/day curcumin + 166 mg/day fermented red rice + 425 mg/day sterol esters and stanols + 2.5 mg/day olive polyphenols 3 months	No decrease in FPG	Derosa et al. (2018)
IFG, IGT (*n* = 64)	180 mg/day curcumin + 1.2 g/day n‐3 PUFAs 12 weeks	FPG, HbA_1c_, FPI, HOMA‐IR, and HOMA‐IS not altered	Thota, Acharya, and Garg (2019)
IFG patients (*n* = 40)	310 mg/day berberine + 500 mg/day *lagerstroemia speciose* + 250 mg/day curcumin + 2.6 mg/day chromium picolinate + 0.30 mg/day folic acid + 220 mg/day α‐lipoic acid 8 weeks	FPG, FPI, and HOMA index decrease	Cicero et al. (2017)
IFG and IGT patients (*n* = 148)	200 mg/day berberine + 200 mg/day curcumin + 300 mg/day inositol + 40 mg/day banaba + 100 mg/day chromium picolinate 3 months	FPG, PPG, HbA_1c_, and HOMA‐IR decrease; FPI increase	Derosa, D'Angelo, Vanelli, and Maffioli (2020)

Abbreviations: AUC, area under the curve; CKD, chronic kidney disease; FPG, fasting plasma glucose; FPI, fasting plasma insulin; GII, post‐prandial insulinemic index; HbA_1c_, glycated hemoglobin; HOMA‐B, homeostatic model assessment of β‐cell function; HOMA‐IR, homeostatic model assessment of insulin resistance; HOMA‐IS, homeostatic model assessment of insulin sensitivity; HOMA index, homeostatic model assessment index; IFG, impaired fasting glucose; IGT, impaired glucose tolerance; NAFLD, non‐alcoholic fatty liver disease; OGTT, oral glucose tolerance test; PPG, post‐prandial plasma glucose; PPI, post‐prandial plasma insulin; T2DM, type 2 diabetes mellitus.

In the trial by Usharani et al. (2008) 72 T2DM patients were treated with curcumin 300 mg/day, atorvastatin 10 mg/day, or placebo for 8 weeks. At the end of treatment, FPG and HbA_1c_ values have not been altered both by the supplement and atorvastatin (Usharani et al., 2008).

Another study conducted on 14 healthy individuals subjected to an OGTT after the assumption of curcumin 6 g or placebo in a single meal showed that the supplement significantly increased PPI levels at 30 (*p* = .048) and 60 min (*p* = .033) after the test respect to the OGTT performed following placebo ingestion. Curcumin also determined an increase of PPI AUC 15 min (+48.30 mUmin/L, +22.20%, *p* = .048), 30 min (+253.80 mUmin/L, +43.40%, *p* = .035), 90 min (+698.00 mUmin/L, +31.60%, *p* = .03), and 120 min (+821.40 mUmin/L, +29.90%, *p* = .02) after OGTT compared to that with placebo. In addition, the supplement raised the post‐prandial insulinemic index (GII) at 90 min (+36.40%, *p* = .024) in response to OGTT respect to the test with placebo. Since curcumin intake did not affect PPG levels, PPG AUC, and post‐prandial glycemic index (GI) at different times during OGTT it has been suggested that the supplement influences insulin secretion (Wickenberg et al., 2010).

Khajehdehi et al. (2011) investigated the effect of curcumin 66.3 mg/day or placebo administration for a period of 2 months in 40 patients with overt T2DM nephropathy. It has been observed that the supplement did not affect FPG and 2 hr‐PPG levels respect to placebo (Khajehdehi et al., 2011).

One study evaluated the efficacy of curcumin on glucose metabolism in 240 IFG and IGT subjects that received 250 mg/day of supplement or placebo for 9 months. It has been found that curcumin decreased FPG, 2 hr‐PPG and HbA_1c_ levels at 3 months (−7.54 mg/dl, − 7.30%, *p* < .01 for FPG; −8.04 mg/dl, −5.60%, *p* < .01 for 2 hr‐PPG; −0.09%, −1.50%, *p* < .01 for HbA_1c_), 6 months (−12.89 mg/dl, − 12.40%, *p* < .01 for FPG; −16.25 mg/dl, −11.30%, *p* < .01 for 2 hr‐PPG; −0.18%, −3.10%, *p* < .01 for HbA_1c_), and 9 months (−17.18 mg/dl, −16.60%, *p* < .01 for FPG; −20.13 mg/dl, −14.00%, *p* < .01 for 2 hr‐PPG; −0.26%, −4.40%, *p* < .01 for HbA_1c_) respect to placebo. The supplement also significantly lowered FPI levels after 9 months of treatment (−0.38 μIU/ml, −2.40%, *p* < .05) and HOMA‐IR at 6 months (−0.64, −15.90%, *p* < .05) and 9 months (−0.81, −20.10%, *p* < .001), whereas increased homeostatic model assessment of β‐cell function (HOMA‐B) (+12.47, +25.40%, *p* < .01) at 9 months compared to placebo. It has also been observed the efficacy of curcumin in retarding T2DM onset. About that none of the patients treated with curcumin developed the disease unlike the 16.4% of subjects in placebo group which was diagnosed with T2DM (Chuengsamarn et al., 2012).

Na et al. (2013) reported that the administration of curcuminoids 300 mg/day or placebo in 100 overweight and obese T2DM subjects for a period of 3 months produced a significant reduction of FPG (−16.04 mg/dl, −10.90%, *p* < .01), HbA_1c_ (−0.97%, −12.10%, *p* = .031), and HOMA‐IR (−1.35, −24.60%, *p* < .01) following supplementation in comparison to placebo (Na et al., 2013).

In a randomized, double‐blind, placebo‐controlled study performed on 71 IGT and controlled T2DM subjects, it has been investigated the effect of *Jiangtang Xiaozhi* 11.36 g/day, an herbal medicine containing 13% curcumin (1.5 g/day) on glucose metabolism. After 16 weeks of treatment, this compound significantly reduced PPG (−14.23 mg/dl, −7.60%, *p* = .03) respect to baseline and FPI (−10.50 mmol/L, −47.50%, *p* = .04) respect to placebo. The herbal medicine also slightly decreased HOMA‐IR without reaching statistical significance (−0.85, −35.00%, *p* = .06) and did not modify FPG and HbA_1c_ levels compared to placebo (Grant et al., 2013).

Another trial evaluated the effect of curcumin 475 mg/day given as adjunct therapy to 8 T2DM patients treated with glyburide 5 mg/day. After 10 days of treatment curcumin plus hypoglycemic drug significantly reduced blood glucose levels within 24 hr and in particular 2 hr‐PPG (after breakfast) (−42.00 mg/dl, −22.30%, *p* < .001), 6 hr‐PPG (after lunch) (−35.40 mg/dl, −21.60%, *p* < .001), and 12 hr‐PPG (after dinner) (−26.70 mg/dl, −13.70%, *p* < .001), respect to glyburide alone (Neerati et al., 2014).

In the report of Yang et al. (2014), 65 metabolic syndrome patients received curcumin extract of 1.89 g/day or a placebo for 12 weeks. The supplement did not significantly modify FPG levels while those of HbA_1c_ showed a mild downward trend in the female subgroup (−0.27%, −4.20%, *p* = .030) respect to baseline (Yang et al., 2014).

A 6‐month trial including 240 T2DM subjects taken 1.5 g/day of curcumin or placebo reported that the supplement reduced significantly FPI and HOMA‐IR at 3 months (−0.34 μIU/ml, −13.80%, *p* = .03 for FPI; −1.87, −31.60%, *p* < .01 for HOMA‐IR) and 6 months (−0.81 μIU/ml, −33.7%, *p* < .01 for FPI; −2.91, −51.40%, *p* < .01 for HOMA‐IR) respect to placebo. At the end of treatment curcumin also slightly lowered FPG (−16.05 mg/dl, −11.50%, *p* = .08) and HbA_1c_ levels (−0.52%, −7.40%, *p* = .09) without reach statistical significance compared to placebo (Chuengsamarn et al., 2014).

A cohort of 250 subjects with metabolic syndrome was divided in 4 groups treated with black seed 1.5 g/day, curcumin 2.4 g/day, and their association (black seeds 900 mg/day and curcumin 1.5 g/day) or placebo respectively for 8 weeks. It was found that curcumin alone did not change FPG levels unlike the combination that significantly decreased them at 4 weeks (−6.60 mg/dl, −5.60%, *p* = .04) and 8 weeks (−14.40 mg/dl, −12.40%, *p* < .001) compared to placebo (Amin et al., 2015).

Rahmani et al. (2016) observed that the assumption of a curcumin amorphous dispersion (500 mg/day equivalent to 70 mg curcuminoids) or placebo for 8 weeks in 80 metabolic syndrome subjects with non‐alcoholic fatty liver disease (NAFLD) (grades 1–3) determined a significant reduction of FPG levels (−10.61 mg/dl, −9.00%, *p* = .048) by comparison to placebo and HbA_1c_ values both respect to baseline (−0.78%, −12.40%, *p* < .001) and to placebo (−2.00%, −26.60%, *p* < .001) following the supplementation (Rahmani et al., 2016).

One study evaluated the efficacy of curcumin 320 mg/day or placebo treatment for 8 weeks in 101 patients with diabetic and non‐diabetic proteinuric chronic kidney disease (CKD). It has been observed a not significant downward trend of FPG levels in subjects with diabetic proteinuric CKD respect to baseline and no alteration of HbA_1c_ values within and between groups after curcumin intervention (Jiménez‐Osorio et al., 2016).

Rahimi et al. (2016) reported that 70 T2DM patients treated with curcumin as nano‐micelle 80 mg/day or placebo for 3 months presented a significant decrease of FPG and HbA_1c_ both to baseline (−15.21 mg/dl, −11.20%, *p* = .049 for FPG; −0.28%, −3.70%, *p* < .001 for HbA_1c_) and to placebo (−9.00 mg/dl, −31.70%, *p* = .004 for FPG; −1.69%, −18.80%, *p* = .02 for HbA_1c_) after intervention.

Another study showed that a cohort of 100 T2DM subjects taking a combination of curcuminoids 500 mg/day and piperine 5 mg/day or placebo for 3 months exhibited a significant decrease of FPG (−9.00 mg/dl, −5.50%, *p* < .001), HbA_1c_ (−0.90%, −12.20%, *p* < .001), FPI (−1.00 μIU/L, −5.00%, *p* = .017), HOMA‐IR (−0.70, −8.60%, *p* = .007), and C‐peptide (−0.50 ng/ml, −11.10%, *p* < .001) respect to baseline after nutraceutical assumption. The supplement also lowered FPG (−17.00 mg/dl, −9.90%, *p* = .048), HbA_1c_ (−0.80%, −11.00%, *p* < .001), and C‐peptide levels (−0.60 ng/ml, −15.00%, *p* < .001) compared to placebo (Panahi et al., 2018). Regarding the use of piperine in association with curcumin it has been demonstrated that this compound seems to enhance curcumin bioavailability by a 20‐fold (Patil, Das, & Balasubramanian, 2016) and pharmacokinetics (Shoba et al., 1998).

In the study of Adab et al. (2019), 80 hyperlipidemic T2DM subjects received 2.1 g/day of curcumin or placebo for a period of 8 weeks. It has been observed that the supplement did not significantly lowered FPG, HbA_1c_, FPI, and HOMA‐IR both to baseline and to placebo.

In a sample of 44 overweight T2DM patients supplemented with 1.5 g/day of curcumin or placebo for 10 weeks, there was a significant decrease in FPG levels (−7.00 mg/dl, −4.40%, *p* = .02) compared to baseline after curcumin intervention, but there was not a significant change of FPI, HbA_1c_, HOMA‐IR, and HOMA‐B within or between groups (Hodaei et al., 2019).

Funamoto et al. (2019) evaluated the effect of a highly absorbable curcumin preparation of 180 mg/day or placebo given to 33 IFG and non‐insulin‐dependent T2DM patients for 6 months. At the end of treatment, the supplement lowered FPG levels, although not significantly (*p* = .079), and did not alter HbA_1c_ values respect to baseline.

Some studies investigated the glucose‐lowering activity of curcumin in combination with other nutraceuticals.

Sukandar et al. (2010) investigated the effect of curcumin and garlic association in 32 T2DM dyslipidemic patients treated with 1.2, 1.6, and 2.4 g/day of the nutraceutical combination or placebo for 12 weeks. It has been observed that the highest dose of supplement significantly decreased 2 hr‐PPG (−60.40 mg/dl, −22.70%, *p* = .032) and HbA_1c_ levels (−0.32%, −2.80%, *p* = .025) compared to baseline. The three doses of nutraceutical combination not significantly reduced FPG values by 18, 11, and 20%, respectively, showing that the highest one resulted in a more effective in decreasing glycemic parameters (Sukandar et al., 2010). The same authors performed a study to compare the efficacy of curcumin and garlic association with oral hypoglycemic agent glibenclamide in 29 T2DM patients with or without dyslipidemia. The subjects were divided into two groups treated with 2.4 g/day of nutraceutical combination and 5 mg/day of the oral hypoglycemic drug, respectively, for a period of 14 weeks. The supplement significantly decreased FPG (−51.05 mg/dl, −26.50%, *p* = .028), 2 hr‐PPG (−91.00 mg/dl, −30.80%, *p* = .019), and HbA_1c_ levels (−2.32%, −22.32%, *p* < .05) respect to baseline. In the nutraceutical combination group, it has been also found a not statistically significant decrease of FPI (−1.33 μIU/ml, −16.30%, *p* = .153) that was greater than that in the glibenclamide group (−0.04 μIU/ml, −0.50%, *p* = .675) (Sukandar et al., 2014).

In a randomized, double‐blind, placebo‐controlled trial were enrolled 80 hypercholesterolemic subjects at low cardiovascular risk that received a nutraceutical association as a capsule each consisting of curcumin extract 250 mg, fermented red rice 166 mg, sterol esters and stanols 425 mg, and olive polyphenols 2.5 mg, once a day for 3 months in addition to diet and physical activity. It has been observed that the supplement did not affect FPG levels both to baseline and placebo (Derosa et al., 2018).

Thota et al. (2019) demonstrated that the combination of 1 g/day curcumin tablets containing 180 mg of curcumin and 2 g/day fish oil capsules providing 1.2 g of EPA + DHA given to 64 dysglycemic patients for 12 weeks did not significantly influence FPG, HbA_1c_, FPI, HOMA‐IR and homeostatic model assessment of insulin sensitivity (HOMA‐IS) by comparison both to baseline and placebo (Thota et al., 2019).

In addition, it has been explored the efficacy of two nutraceuticals associations on glucose metabolism (combination of curcumin, *L. speciose*, berberine, α‐lipoic acid, chromium picolinate, and folic acid combination or curcumin, banaba, berberine, inositol, and chromium picolinate) as already discussed (Cicero et al., 2017; Derosa, D'Angelo, Vanelli, & Maffioli, 2020).

### 3.4 Guar gum [*Cyamopsis tetragonolobus* (L) Pers]

Guar gum is a natural polysaccharide consisting of a polymer of d‐galactose and d‐mannose named galactomannan. It is extracted from the seed endosperm of *C. tetragonolobus*, a plant belonging to the family leguminosae that is grown in India, Pakistan, Sudan, and some zones of United States. Guar gum is able to modify its rheological properties (Dodi, Hritcu, & Popa, 2011; Iqbal & Hussain, 2013). The high content of hydroxyl groups in this polysaccharide increases its ability to form hydrogen bonds when added to water giving a considerable viscosity and thickening to the solution. Besides the ability to thicken, emulsify, bind, and forms gels or films, guar gum is characterized by a rapid solubility in cold water, large pH stability, and biodegradability. Thanks to these peculiar properties guar gum are extensively used in several fields for various applications including water purification, drug delivery, pharmaceutical, textile, cosmetic, and food industries. About its application in pharmacotherapy, guar gum behaves as a water‐soluble and bulk‐forming laxative fiber resulting in effective in stimulating peristaltic movements thus relieving constipation and other chronic bowel disorders. Guar gum is also used in the treatment of cholera and diarrhea (Tripathy & Das, 2013). Moreover, it showed cholesterol and glucose lowering actions as well as chemo‐preventive and antiinflammatory properties (G. Sharma et al., 2018; Thombare, Jha, Mishra, & Siddiqui, 2016). In particular, the hypoglycemic activity of guar gum was investigated.

#### 3.4.1 Mechanisms of action

**FIGURE 4:**
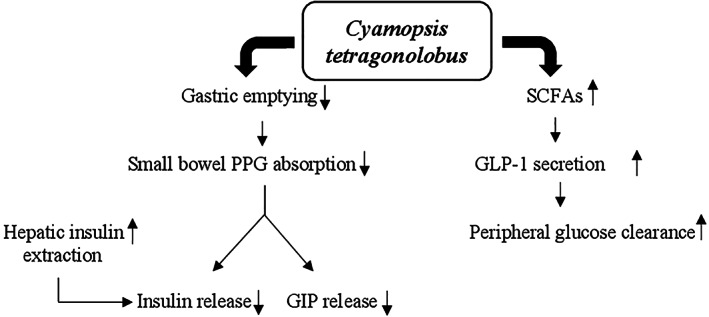
Potential mechanisms for hypoglycemic action of *Cyamopsis tetragonolobus*. GIP, gastric inhibitory polypeptide; GLP‐1, glucagon‐like peptide‐1; PPG, post‐prandial glucose; SCFAs, short‐chain fatty acids

The potential hypoglycemic mechanisms of action of guar gum are mainly linked to its unique properties. The gel‐forming ability to increase the viscosity of intragastric content involves a slowing of gastric emptying and glucose absorption in the small intestine where guar gum acts as a physical barrier thus decreasing the exposure of glucose molecules to bowel mucosa cells and slowing their diffusion through the small intestinal mucosa (Blackburn, Holgate, & Read, 1984; French & Read, 1994; Lin, Doty, Reedy, & Meyer, 1989; Meyer, Gu, Jehn, & Taylor, 1988). The presence of glucose in this organ promotes the release of some molecules such as insulin and incretin hormones, glucagon‐like peptide‐1 (GLP‐1), and gastric inhibitory polypeptide (GIP) (Lavin et al., 1998; Schirra et al., 1996). In association with decreased bowel glucose absorption guar gum also reduces insulin and GIP secretion (Simões Nunes & Malmlöf, 1992). Moreover, the insulin levels are attenuated by increasing liver extraction (HIE) (Gatenby, Ellis, Morgan, & Judd, 1996). In addition, the hypoglycemic action of guar gum is mediated by the short‐chain fatty acids produced by intestinal bacterial fermentation that stimulate GLP‐1 secretion thus increasing peripheral glucose clearance (den Besten et al., 2015) (Figure 4).

#### 3.4.2 Clinical trials

Several trials on guar gum hypoglycemic action agree that this soluble fiber is effective in reducing PPG and PPI levels (Table 4).

**TABLE 4:** Summary of human studies on glucose‐lowering effect of *Cyamopsis tetragonolobus*

Subjects	Drug treatment and period	Results	References
Healthy subjects (*n* = 4)	14.5 g guar flour 1 occasion	PPG and PPI decrease	D. J. Jenkins, Leeds, Gassull, Cochet, and Alberti (1977)
Healthy subjects (*n* = 4/5) Diabetes insulin‐dependent patients (*n* = 6)	Mixed meal with 10 g guar High carbohydrate meal with 10 g guar 1 occasion	PPG, PPI, and GIP decrease	L. M. Morgan, Goulder, Tsiolakis, Marks, and Alberti (1979)
Healthy subjects (*n* = 6) T2DM patients (*n* = 17)	20 g/day guar gum 1 or 3 weeks	FPG, PPG, and PPI decrease	Smith and Holm (1982)
Obesity patients (*n* = 9)	10 g guar gum 1 occasion 2 20 g/day guar gum 8 weeks	PPG decrease	Krotkiewski (1984)
T2DM patients (*n* = 10)	8 g/day guar gum 6 weeks	FPG, FPI, and HbA_1c_, decrease M/I ratio increase	Tagliaferro et al. (1985)
Diabetes insulin‐dependent patients (*n* = 28)	Wheat flour bread with 29 g/day guar gum Wheat flour bread with 33 g/day wheat bran 3 months	PPG and HbA_1c_ decrease	Vaaler et al. (1986)
Healthy subjects (*n* = 7) T2DM patients (*n* = 8) Diabetes insulin‐dependent patients (*n* = 7)	3.2 g guar gum 1 occasion	PPG decrease	Ceffa, Sartori, and Carena (1987)
T2DM patients (*n* = 29)	15 g/day guar gum 8 weeks	FPG, PPG, HbA_1c_, and, C‐peptide no effect	Holman, Steemson, Darling, and Turner (1987)
Healthy subjects (*n* = 7) T2DM patients (*n* = 5)	5 g guar gum 1 occasion	Glucose absorption decrease	Upadhyaya, Raghuram, and Krishnaswamy (1988)
Type 1 diabetes patients (*n* = 9)	20 g/day guar gum 4 weeks	PPG and daily insulin requirements decrease	Ebeling et al. (1988)
T2DM patients (*n* = 15)	15 g/day guar gum 48 weeks	FPG, HbA_1c_, PPG, PPI, and post‐prandial C‐peptide decrease	Groop, Aro, Stenman, and Groop (1993)
T2DM patients (*n* = 11)	9 g guar gum 1 occasion	PPG and PPI decrease	Russo et al. (2003)
Hypertension, overweight patients (*n* = 141)	10.5 g/day PHGG 10.5 g/day psyllium 6 months	FPG, HbA_1c_, FPI, and HOMA index decrease (PHGG) FPG, HbA_1c_, FPI, and HOMA index decrease (psyllium)	Cicero et al. (2007)
Metabolic syndrome patients (*n* = 141)	10.5 g/day PHGG 10.5 g/day psyllium 6 months	FPG, HbA_1c_, FPI, and HOMA index decrease (PHGG) FPG, HbA_1c_, FPI, and HOMA index decrease (psyllium)	Cicero, Derosa, Di Gregori, et al. (2010); Cicero, Derosa, Bove, et al. (2010)
T2DM patients (*n* = 44)	10 g/day PHGG 6 weeks	HbA_1c_ decrease	Dall'Alba et al. (2013)
T2DM patients (*n* = 57)	HFD (5.4 g soluble fiber) HFS (5.4 g soluble fiber, 60% PHGG, 40% inulin) UF (0.8 g soluble fiber)	iAUC plasma glucose decrease	De Carvalho et al. (2017)
Controls (*n* = 30) T2DM patients (*n* = 30)	10 g/day herbal powder (4 guar gum + 0.1 g *Cephalandra indica* + 0.6 g *Trigonella faenum graecum* + 0.2 g *Gymnema sylvestre*) 4 weeks	FPG, 1 hr‐PPG, 2 hr‐PPG, and 3 hr‐PPG decrease	Bhardwaj, Dasgupta, Prashar, and Kaushal (1994)
Prediabetes patients (*n* = 11) T2DM patients (*n* = 13)	15 g whey protein + 3 g lactose + 5 g guar gum + 1 g flavor material 2 days	3 hr‐PPG decrease at all time points except 120 min	Clifton, Galbraith, and Coles (2014)
T2DM patients (*n* = 79)	17 g whey + 5 g guar gum 12 weeks	PPG at 30 and 60 min, HbA_1c_ decrease	Watson et al. (2019)

Abbreviations: FPG, fasting plasma glucose; FPI, fasting plasma insulin; GIP, gastric inhibitory polypeptide; HbA_1c_, glycated hemoglobin; HFD, high fiber from the diet; HFS, high fiber from a supplement; HOMA index, homeostatic model assessment index; iAUC, incremental area under curve; PHGG, partially hydrolyzed guar gum; PPG, post‐prandial glucose; PPI, post‐prandial insulin; T2DM, type 2 diabetes mellitus; UF, usual fiber.

One study performed on 4 healthy volunteers subjected to a liquid test meal to which added 14.5 g guar flour showed that PPG was significantly reduced at 30 min (−28.10 mg/dl, −24.60%, *p* < .05) as well as PPI at 15 min (−9.00 mU/L, −40.90%, *p* < .05) and 30 min (−35.00 mU/L, −61.40%, *p* < .05) respect to control (D. J. Jenkins et al., 1977).

L. M. Morgan et al. (1979) investigated the effect of two types of meals with and without the addition of guar flour on PPG, PPI, and gut hormones in healthy and insulin‐dependent diabetics. A mixed meal containing 10 g guar was administered to five normal subjects and six diabetics. The addition of guar significantly decreased the post‐prandial rise in GIP (47% in normal subjects and 30% in diabetics) and blood glucose levels (48% in normal subjects and 58% in diabetics). The supplement also caused a 48% decrease in PPI only in healthy subjects. A high carbohydrate meal with 10 g guar was given to four normal subjects and six diabetics. The presence of this fiber caused a reduction in PPG (78%) and in PPI (59%) in healthy and a lowering in maximum post‐prandial GIP (71%) and blood glucose (68%) in diabetics (L. M. Morgan et al., 1979).

A similar trend for PPG and PPI was observed in 6 healthy subjects and 17 diabetics treated with 10 g guar gum, twice daily, taken before meals, for periods of 1 or 3 weeks, respectively. At the end of treatment, it has been also shown a significant decrease in FPG levels in diabetics compared to baseline values (Smith & Holm, 1982).

In the study by Krotkiewski (1984), it has been evaluated the acute and long‐term effects of guar gum on PPG in nine obese subjects. In the acute study, the patients received a standardized meal with or without 10 g guar gum and it has been demonstrated that the supplement significantly decreased PPG levels at 30 min (−9.01 mg/dl, −9.80%, *p* < .05) respect to control. In the long‐term study, the subjects consumed 10 g of guar gum twice daily usually before lunch and dinner for 8 weeks. At the end of treatment, the supplement further reduced PPG levels at 30 min (*p* < .05) in those patients with the highest PPG levels (Krotkiewski, 1984).

Guar gum in the form of flavored powder was administered at the dose of 8 g/day in addition to the usual diet in 10 T2DM patients. After 6 weeks of supplementation, it has been found a significant decrease in FPG (−13.00 mg/dl, −9.60%, *p* < .05), FPI (−3.00 μU/ml, −23.10%, *p* < .05), and HbA_1c_ levels (−0.57%, −6.70%, *p* < .05) compared to baseline. Guar gum also produced a significant increase in M/I ratio (+10.00, +69.90%, *p* < .025) indicating an improvement in peripheral sensitivity to insulin (Tagliaferro et al., 1985).

In another trial, 28 insulin‐dependent diabetics were given different dietary regimens consisting of wheat flour bread (run‐in period) subsequently enriched with guar gum 29 g/day or wheat bran 33 g/day for periods of 3 months each. It has been shown that guar gum supplementation resulted in a significant decrease of PPG levels (−2.30 mg/dl, −19.20%, *p* < .05) after 3 months and HbA_1c_ values after 1 month (−0.70%, −6.70%, *p* < .05) and at the end of treatment (−0.80%, −7.60%, *p* < .05) respect to run‐in period (Vaaler et al., 1986).

Ceffa et al. (1987) reported the effects on morning post‐prandial glycemic and insulin metabolism of a test meal with or without 3.2 g guar gum given to 22 subjects including 8 T2DM non‐insulin‐dependent patients, 7 insulin‐dependent diabetics, and 7 healthy individuals. It has been observed that the supplement significantly reduced PPG at 30 min (−46.00 mg/dl, −23.50%, *p* < .05) and 60 min (−50.00 mg/dl, −22.20%, *p* < .05) in insulin and non‐insulin‐dependent diabetics, respectively (Ceffa et al., 1987).

However, Holman et al. (1987) demonstrated that in 29 T2DM non‐insulin‐dependent patients that took 15 g/day guar gum or placebo with main meals for 8 weeks, the supplement did not affect FPG, PPG, and HbA_1c_ levels as well as basal or incremental plasma C‐peptide values (Holman et al., 1987).

On the contrary, Upadhyaya et al. (1988) showed that five T2DM non‐insulin‐dependent patients and seven normal subjects subjected to OGTT with or without 5 g guar gum, presented a significant reduction in glucose absorption that was higher in diabetics (−20%, *p* < .05) than in healthy (−13.80%, *p* < .05) (Upadhyaya et al., 1988).

A 4‐week study including nine type 1 diabetic patients given 20 g/day of guar gum or placebo before meals reported that the supplement significantly reduced PPG levels both after breakfast (−39.64 mg/dl, −22.20%, *p* < .02) and lunch (−30.63 mg/dl, −18.90%, *p* < .05) compared to placebo. Moreover, the daily insulin requirement resulted lower at the end of guar gum supplementation than placebo (39.60 ± 2.70 U vs. 40.50 ± 2.60 U, *p* < .02) (Ebeling et al., 1988).

Groop et al. (1993) evaluated the effects of guar gum on glycemic control and insulin secretion in 15 T2DM non‐insulin‐dependent and diet‐treated subjects that received 15 g/day of fiber for 48 weeks. The supplementation was preceded and followed by an 8‐week period of placebo. During guar gum treatment mean FPG levels were reduced not significantly (−5.40 mg/dl, −3.20%, *p* = .231) respect to placebo period 1 and the lowest values were achieved after 16 weeks of supplementation (−9.01 mg/dl, −5.30%). Instead, FPG concentrations were significantly increased in placebo period 2 (+19.82 mg/dl, +12.00%, *p* = .004) compared to guar gum therapy. The supplement significantly decreased HbA_1c_ levels (−0.50%, −5.60%, *p* < .015) respect to the first placebo period recording the highest reduction at 32 week of supplementation (−0.80%, −8.90%). However, HbA_1c_ values resulted unchanged during the second placebo period. The patients were also taken a standardized test meal both after placebo periods and after 16 and 48 weeks of guar gum treatment. It has been reported that plasma glucose and insulin responses to test meals were significantly reduced after 16 weeks of supplementation (*p* = .017 for plasma glucose and *p* = .012 for insulin) compared to the first placebo period. Moreover, after 48 weeks of guar gum treatment, the responses of both parameters did not differ from those of placebo periods and from the response after 16 weeks on the supplement. It has been also observed that post‐prandial C‐peptide response to test meal was significantly increased after 16 weeks of supplementation (*p* = .032) with respect to the first placebo period and this rise was higher after 48 weeks of guar gum therapy either versus placebo period 1 or 16 weeks on the supplement (*p* = .001 for both). At the end of the second placebo period, post‐prandial C‐peptide response diminished but remained significantly higher than that of the first placebo period and of 16 weeks on guar gum (*p* = .001 for both) (Groop et al., 1993).

Another study showed that 11 T2DM patients after having a drink containing 50 g glucose with or without 9 g guar gum exhibited PPG and PPI values significantly reduced starting from 15 min (−23.42 mg/dl, −13.00%, *p* < .05) and 30 min (−17.00 μU/ml, −38.60%, *p* < .01), respectively following guar gum treatment compared to control (Russo et al., 2003).

A 6‐month trial included 141 hypertensive and overweight subjects randomized to receive partially hydrolyzed guar gum (PHGG) or psyllium husk powder at the dose of 3.5 g thrice day 20 min before the main meals or standard diet. Guar gum supplementation significantly reduced FPG at 4 months (−10.00 mg/dl, −16.40%, *p* = .009) and at 6 months (−12.00 mg/dl, −21.80%, *p* = .009) whereas decreased HbA_1c_ (−0.70%, −9.20%, *p* < .001), FPI (−2.70 μU/ml, −10.80%, *p* = .008), and HOMA‐IR (−1.20, −16.70%, *p* = .008) at the end of treatment respect to baseline. The psyllium intake determined a significant decrease in FPG (−18.00 mg/dl, −16.40%, *p* < .001 after 4 months; −24.00 mg/dl, −21.80%, *p* < .001 after 6 months), HbA_1c_ (−0.50%, −7.50%, *p* < .01 after 4 months; −0.70%, −10.40%, *p* < .01 after 6 months), FPI (−3.10 μU/ml, −12.30%, *p* = .005 after 4 months; −5.10 μU/ml, −20.20%, *p* < .001 after 6 months), and HOMA‐IR values (−2.10, −28.40%, *p* < .001 after 4 months; −2.90, −39.20%, *p* < .001 after 6 months) compared to baseline. It has also been observed that FPG and HOMA‐IR values were lower after 4 months (−6.00 mg/dl, −6.10%, *p* < .01 for FPG; −1.10, −17.20%, *p* < .01 for HOMA‐IR) and 6 months (−10.00 mg/dl, −10.40%, *p* < .01 for FPG; −1.50, −25.00%, *p* < .01 for HOMA‐IR) while FPI levels were inferior at the end of treatment (−2.10 μU/ml, −9.50%, *p* < .01) in psyllium group in comparison to guar gum one (Cicero et al., 2007).

The same authors obtained similar results in another study whose design was identical to that described above and in which were enrolled 141 patients with metabolic syndrome (Cicero, Derosa, Bove, et al., 2010).

In the study by Dall'Alba et al. (2013), 44 T2DM patients followed their usual diet or added 10 g/day of PHGG at lunch and dinner for 6 weeks. The supplement reduced HbA_1c_ values after 4 weeks (−0.24%, −3.50%, *p* < .05) respect to baseline and at the end of treatment (−0.07%, −1.10%, *p* < .05) compared to 4 weeks (Dall'Alba et al., 2013).

De Carvalho et al. (2017) evaluated the acute effect of soluble fiber ingestion from foods or supplements comprising 60% PHGG and 40% inulin powder on PPG and PPI in 57 T2DM patients. These subjects received breakfasts containing a high amount of fiber from food (HFD) or supplement (HFS) (soluble fiber 5.4 g) and the usual amount of fiber (UF) (soluble fiber 0.8 g). It has been found that the incremental AUC (iAUC) for plasma glucose resulted lower in HFD (−1,666.00 mg dl^−1^ min^−1^, −17.50%, *p* = .014) and HFS (−1,680.00 mg dl^−1^ min^−1^, −17.60%, *p* = .037) meals respect to UF breakfast whereas there were no differences among insulin iAUCs of tested meals (de Carvalho et al., 2017).

The hypoglycemic action of guar gum in combination with other nutraceuticals was also studied.

Bhardwaj et al. (1994) evaluated the hypoglycemic effect of an herbal powder consisting of guar gum 4.0 g, *Cephalandra indica* 0.1 g, *Trigonella faenun graecum* 0.6 g, and *Gymnema sylvestre* 0.2 g. This nutraceuticals association was given to 30 T2DM non‐insulin‐dependent and 30 control subjects at the dose of 5 g prior to breakfast and dinner for 4 weeks. These patients also underwent OGTT. It has been observed that herbal powder significantly decreased FPG (−67.87 mg/dl, −38.30%, *p* < .001) 1 hr‐PPG (−103.26 mg/dl, −38.60%, *p* < .001), 2 hr‐PPG (−121.60 mg/dl, −41.30%, *p* < .001), and 3 hr‐PPG levels (−83.27 mg/dl, −38.90%, *p* < .001) in diabetics respect to baseline (Bhardwaj et al., 1994).

In the study by Clifton et al. (2014), 11 subjects with prediabetes and 13 with moderately well‐controlled T2DM took a test drink composed of cold water with whey protein 15 g, lactose 3 g, guar gum 5 g, and flavor material 1 g for 2 days or cold water on the other 2 days 15 min before breakfast. Three‐hour glucose measurement performed with a finger prick test showed a significant reduction at all time points except 120 min recording the highest decrease at 45 min (−37.84 mg/dl, −24.40%, *p* < .0001) respect to control. The lowering in blood glucose over 3 hr was similar for both patients with prediabetes and T2DM (Clifton et al., 2014).

A 12‐week trial was conducted on 79 T2DM patients that received a shake containing a combination of whey 17 g and guar gum 5 g or placebo 15 min before breakfast and dinner. It has been observed a significant reduction of PPG levels at 30 and 60 min (−36.04 mg/dl, −15.20%, *p* < .05) both after 1 week and at the end of supplementation compared to placebo. The nutraceuticals association also significantly lowered HbA_1c_ values (−2.20%, −2.00%, *p* < .05) respect to placebo (Watson et al., 2019).

### 3.5 Omega‐3

Omega‐3 polyunsaturated fatty acids (n‐3 PUFAs) are bioactive compounds including eicosapentaenoic acid (EPA, 20:5n‐3) and docosahexaenoic acid (DHA, 22:6n‐3) mainly derived from fish and seafood, and alpha‐linolenic acid (ALA, 18:3n‐3) from plant sources such as leafy greens, seeds, and nuts. It has been supposed that n‐3 PUFAs supplementation may positively affect glucose metabolism (Storlien et al., 1991).

#### 3.5.1 Mechanisms of action

**FIGURE 5:**
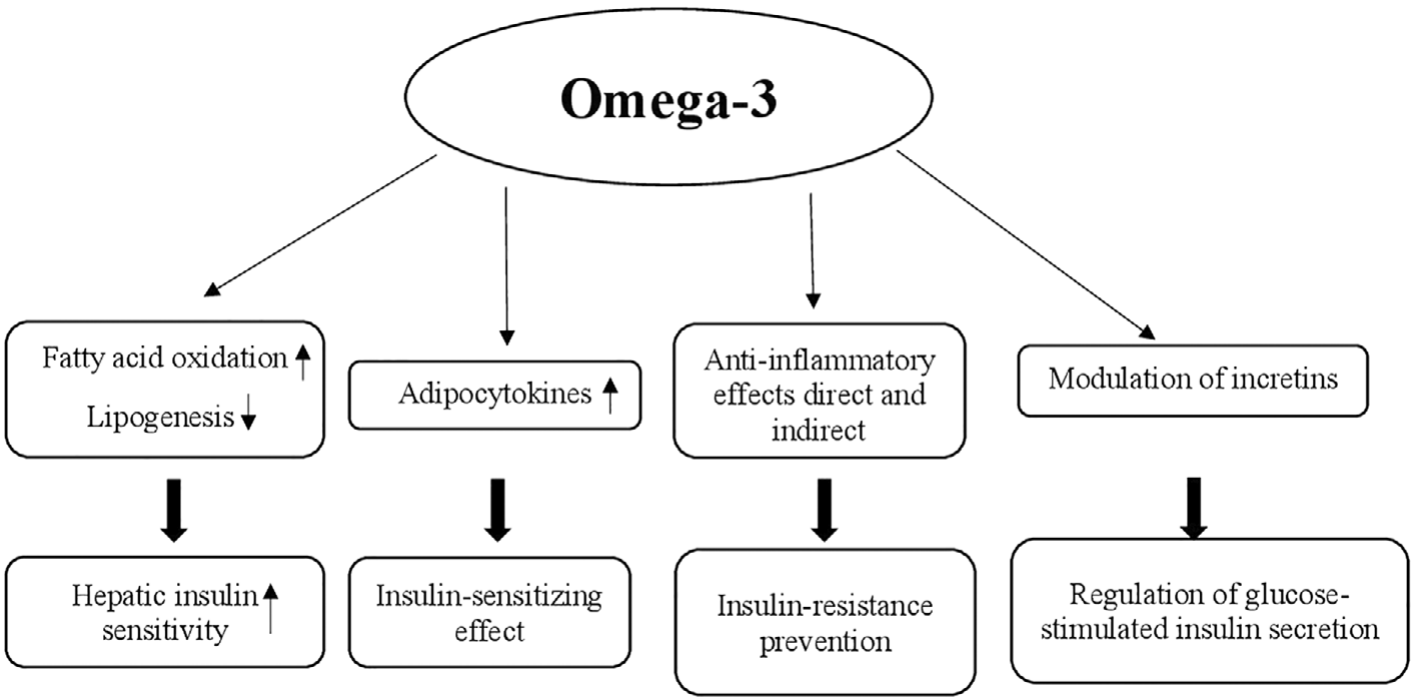
Possible mechanisms of action for Omega‐3 in glycemic control

To date, the mechanisms through which n‐3 PUFAs improve glycemic control are still unclear (Lalia & Lanza, 2016). However, animal model studies showed some potential mechanisms which consist of (a) improvement of hepatic insulin sensitivity (Matsuura et al., 2004) via enhanced fatty acid oxidation, and decreased lipogenesis (Kuda et al., 2009; Wu et al., 2012), (b) insulin‐sensitizing effect by increased release of adipocytokines such as adiponectin and leptin (Rossi et al., 2005), and (c) prevention of insulin‐resistance via direct (D. Y. Oh et al., 2010) and indirect (González‐Périz et al., 2009; Serhan, Chiang, & Van Dyke, 2008) antiinflammatory effects. In addition, n‐3 PUFAs are able to modulate incretins that are involved in glucose‐stimulated insulin release (Flachs, Rossmeisl, & Kopecky, 2014) (Figure 5).

#### 3.5.2 Clinical trials

Clinical trials present in the literature reported controversial findings about the effect of n‐3 PUFAs on glycemic control (Table 5).

**TABLE 5:** Summary of clinical trials on omega‐3 glycemic control

Subjects	Drug treatment and period	Results	References
T2DM non‐insulin dependent patients (*n* = 6)	5.5 g/day n‐3 PUFAs 1 months	FPG and HbA_1c_ increase FPI slightly not significant decrease	Glauber, Wallace, Griver, and Brechtel (1988)
T2DM non‐insulin dependent patients (*n* = 8)	8.0 g/day n‐3 PUFAs 8 weeks	FPG increase FPI and HbA_1c_ not altered	Friday et al. (1989)
T2DM non‐insulin dependent patients (*n* = 10)	3.0 g/day n‐3 PUFAs 3 weeks	FPG increase FPI and C‐peptide not altered	Borkman et al. (1989)
Type 1 diabetes mellitus patients (*n* = 12)	2.7 g/day n‐3 PUFAs 10 weeks	FPG and HbA_1c_ not altered	Rillaerts, Engelmann, Van Camp, and De Leeuw (1989)
T2DM non‐insulin dependent patients (*n* = 80)	3.0 g/day n‐3 PUFAs 6 weeks	FPG increase HbA_1c_ not altered	Hendra et al. (1990)
T2DM non‐insulin dependent, obesity patients (*n* = 20)	3.1 g/day n‐3 PUFAs 3 weeks	FPG, PPG and HbA_1c_ not altered	Pelikánová, Kohout, Válek, Kazdová, and Base (1993)
T2DM non‐insulin dependent, hyperlipidemia patients (*n* = 40)	5.0 g/day n‐3 PUFAs 10.0 g/day n‐3 PUFAs 12 weeks	FPG and HbA_1c_ not altered	W. A. Morgan, Raskin, and Rosenstock (1995)
T2DM non‐insulin dependent patients (*n* = 414)	2.6 g/day n‐3 PUFAs 2 months 2 1.7 g/day n‐3 PUFAs 10 months	FPG, FPI, and HbA_1c_ not altered	Sirtori et al. (1998)
T2DM patients (*n* = 26)	1.8 g/day n‐3 PUFAs 2 months	FPG, FPI, HbA_1c_, HOMA‐B, and HOMA‐IS not altered	Kabir et al. (2007)
Combined dyslipidemia patients (*n* = 333)	3.0 g/day n‐3 PUFAs 6 months	FPG, FPI, and HOMA‐IR not altered	Derosa et al. (2009)
T2DM patients (*n* = 97)	2.7 g/day n‐3 PUFAs 12 weeks	FPG and HbA_1c_ not altered	Wong et al. (2010)
Elderly (*n* = 124)	300 mg/day n‐3 PUFAs 6 months	FPG, FPI, and HOMA‐IR not altered	Fakhrzadeh et al. (2010)
Hypertrigliceridemia, untreated normal‐high blood pressure patients (*n* = 111)	2.0 g/day n‐3 PUFAs 12 months	FPG not altered except an increase at 3 months	Cicero, Derosa, Di Gregori, et al. (2010)
Combined dyslipidemia patients (*n* = 167)	3.0 g/day n‐3 PUFAs 6 months	FPG decrease FPI and HOMA‐IR not altered M value and TGR increase	Derosa et al. (2011) Derosa, Cicero, et al. (2012)
IFG, IGT patients (*n* = 12.536)	1.0 g/day n‐3 PUFAs Follow‐up 6.2 years	FPG and HbA_1c_ not altered	ORIGIN Trial Investigators et al. (2012)
T2DM, hypertension patients (*n* = 41)	900 mg/day n‐3 PUFAs 540 mg/day n‐3 PUFAs 1 months	QUICKI decrease in 85.7% of subjects FPG, HbA_1c_, and HOMA‐IR decrease in 42.9, 35.7, and 35.7% of subjects	Crochemore, Souza, de Souza, and Rosado (2012)
T2DM patients (*n* = 72)	1.85 g/day n‐3 PUFAs 8 weeks	FPG, FPI, and HOMA‐IR not altered	Mansoori et al. (2015)
IFG, IGT, overweight/obesity patients (*n* = 281)	3.0 g/day n‐3 PUFAs 18 months	FPG, FPI, and HOMA‐IR decrease	Derosa, Cicero, D'Angelo, Borghi, and Maffioli (2016)
T2DM patients (*n* = 54)	520 mg/day n‐3 PUFAs 6 months	FPG and HbA_1c_ decrease FPI and HOMA‐IR increase	Jacobo‐Cejudo et al. (2017)
IFG, IGT patients (*n* = 64)	1.2 g/day n‐3 PUFAs + 180 mg/day curcumin 12 weeks	FPG, HbA_1c_, FPI, HOMA‐IR, and HOMA‐IS not altered	Thota et al. (2019)

Abbreviations: FPG, fasting plasma glucose; FPI, fasting plasma insulin; HbA_1c_, glycated hemoglobin; HOMA‐B, homeostatic model assessment of β‐cell function; HOMA‐IR, homeostatic model assessment of insulin resistance; HOMA‐IS, homeostatic model assessment of insulin sensitivity; IFG, impaired fasting glucose; IGT, impaired glucose tolerance; n‐3 PUFAs, omega‐3 polyunsaturated fatty acids; QUICKI, quantitative insulin sensitivity check index; T2DM, type 2 diabetes mellitus; TGR, total glucose requirement.

First human studies showed that the administration of this supplement in T2DM patients did not improve glucose metabolism. In this regard, Glauber et al. (1988) observed an increase in FPG (+39.64 mg/dl, +16.80%, *p* = .03) and HbA_1c_ (+1.80%, +14.90%, *p* = .03) levels in 6 men with T2DM non‐insulin‐dependent treated daily with 18 g fish oil concentrate containing 5.5 g of n‐3 PUFAs (3.3 g EPA and 2.2 g DHA) and 108 mg of cholesterol for a period of 1 month. In addition, there was a slightly not significant decrease in FPI values (−2.88 μIU/ml, −18.20%, *p* = .22) compared to baseline. It has been also seen that the FPG levels returned to baseline values after the supplement discontinuation (Glauber et al., 1988).

Another trial evaluated the effect of n‐3 PUFAs assumption, 8 g/day for 8 weeks, in 8 T2DM non‐insulin‐dependent men. The supplement was administered in the form of capsules each containing 300 mg EPA, 200 mg DHA, and 1 mg cholesterol. At the end of treatment FPG concentration was increased (+34.00 mg/dl, +21.40%, *p* = .005) while FPI and HbA_1c_ values were not altered (Friday et al., 1989).

Borkman et al. (1989) reported that the administration of 10 g/day fish oil containing 18% EPA and 12% DHA for 3 weeks in 10 T2DM non‐insulin‐dependent patients produced a significant increase of FPG (+18.02 mg/dl, +14.00%, *p* < .05) without changing FPI and C‐peptide levels in comparison to baseline values that were those of subjects treated with standard diabetic diet alone. It has been also seen that FPG levels returned to baseline ones after a wash‐out period of 3 weeks (Borkman et al., 1989).

In contrast to the above, one trial demonstrated that 12 type 1 diabetic patient supplemented with 2.7 g/day of n‐3 PUFAs (1.8 g EPA and 0.9 g DHA) did not exhibit alterations in FPG and HbA_1c_ values after 10 weeks of treatment (Rillaerts et al., 1989).

However, in the study by Hendra et al. (1990) involving 80 T2DM non‐insulin‐dependent subjects treated with 10 g/day of fish oil containing 1.8 g EPA and 1.2 g DHA for 6 weeks it has been recorded an increase in FPG levels both at 3 and 6 weeks but it was significant only after 3 weeks of treatment (+25.95 mg/dl, [95% confidence interval: 0.34–2.5], *p* = .01) compared to control. There was also no significant alteration in HbA_1c_ values (Hendra et al., 1990).

As already seen by Rillaerts et al. (1989), subsequent studies showed neutral effects of this supplement on glucose metabolism. About this, it has been reported that 3.1 g/day of n‐3 PUFAs administered to 20 obese with T2DM non‐insulin‐dependent for 3 weeks did not alter FPG, PPG, and HbA_1c_ levels (Pelikánová et al., 1993).

W. A. Morgan et al. (1995) investigated the efficacy of fish oil supplementation, in capsule form, in 40 T2DM non‐insulin‐dependent with hyperlipidemia. The EPA and DHA content of each capsule was 28.8 and 27.3%, respectively. It has been found that the fish oil administration at the dose of 9 and 18 g/day for 12 weeks did not significantly affect FPG and HbA_1c_ values (W. A. Morgan et al., 1995).

Sirtori et al. (1998) found that 414 T2DM non‐insulin‐dependent patients treated with n‐3 PUFAs in the form of ethyl ester at the dose of 2.6 g/day (1,530 mg EPA and 1,050 mg DHA) for 2 months and 1.7 g/day (1,020 mg EPA and 700 mg DHA) for 4 months did not display modifications in FPG, FPI, and HbA_1c_ levels respect to placebo. The assumption of n‐3 PUFAs, 1.7 g/day, for a further 6 months confirmed the absence of a worsening in glycemic profile after 1 year of supplementation (Sirtori et al., 1998).

Another study reported that 2‐month administration of 3 g/day of fish oil containing 1.08 g EPA and 0.72 g DHA in 26 postmenopausal women with T2DM did not influence FPG, FPI, and HbA_1c_ concentrations as well as HOMA‐B and HOMA‐IS values (Kabir et al., 2007).

Derosa et al. (2009) found that daily supplementation with 3 g of n‐3 PUFAs consisting of EPA and DHA with a 0.9–1.5 proportion for 6 months in 333 patients with combined dyslipidemia did not affect FPG and FPI levels along with HOMA‐IR compared to baseline (Derosa et al., 2009).

Wong et al. (2010) reported that 97 T2DM patients treated with fish oil at the dose of 4 g/day containing 42% of EPA and 25% of DHA did not show any change in FPG and HbA_1c_ levels after 12 weeks of supplementation (Wong et al., 2010).

In accordance with the above, a 6‐month trial including 124 elderly who received 1 g/day of fish oil capsule with 300 mg n‐3 PUFAs (180 mg EPA and 120 mg DHA) proved that the supplement did not cause any variation in FPG and FPI levels as well as in HOMA‐IR values both respect to baseline and placebo (Fakhrzadeh et al., 2010).

In a retrospective study, it has been observed the efficacy of 2 g/day n‐3 PUFAs containing at least 85% of EPA and DHA in the ratio of 0.9–1.5 in 111 subjects with hypertriglyceridemia and untreated normal‐high blood pressure. After 12 months of treatment, no variation in FPG levels was recorded except for a significant increase at 3 months (+2.10 mg/dl, +2.10%, *p* < .01) respect to baseline (Cicero, Derosa, Di Gregori, et al., 2010).

In contrast, it has been reported that the administration of 3 g/day n‐3 PUFAs with content of 1,200 mg EPA and 1,350 mg DHA for 6 months in 167 patients with combined dyslipidemia produced a significant decrease in FPG levels (−9.00 mg/dl, −10.10%, *p* < .05) compared to a baseline without altering FPI and HOMA‐IR values. In patients undergoing euglycemic hyperinsulinemic clamp the supplement also increased M value (+1.89 μmol min^−1^ kg^−1^, +33.90%, *p* < .05) and total glucose requirement (+3.50 μmol min^−1^ kg^−1^, +9.90%, *p* < .05) by comparison to baseline (Derosa et al., 2011; Derosa, Cicero, et al., 2012).

However, it has been also shown that 12.536 dysglycemic patients at high risk of cardiovascular events treated with 1 g/day of n‐3 PUFAs containing 465 mg EPA and 375 mg DHA, during a median follow‐up of 6.2 years did not display changes in FPG and HbA_1c_ levels as compared to placebo (ORIGIN Trial Investigators et al., 2012).

Another study evaluated the influence of n‐3 PUFAs in 41 hypertensive and T2DM women. They were divided into 3 groups treated with 2.5 g/day fish oil (547.5 mg EPA and 352.5 mg DHA), 1.5 g/day fish oil (328.5 mg EPA and 211.5 mg DHA), and placebo, respectively. After 1 month of supplementation, despite the absence of statistical differences, in the group that received the lower dose of n‐3 PUFAs was found a greater frequency of FPG (42.9%), and HbA_1c_ (35.7%) decrease with respect to the other ones, while in the group treated with the largest dose of supplement the Quantitative Insulin Sensitivity Check Index (QUICKI) which represents the insulin sensitivity was reduced in 85.7% of women. In addition, HOMA‐IR decreased in 35.7% of patients supplemented with the lower dose of n‐3 PUFAs similarly to the placebo group (38.5%) (Crochemore et al., 2012).

Afterward it has been demonstrated that 2.4 g/day of fish oil (400 mg EPA and 1,450 mg DHA) administered to a sample of 72 T2DM subjects for 8 weeks did not affect FPG and FPI levels along with HOMA‐IR values respect to placebo (Mansoori et al., 2015).

As previously demonstrated by Derosa, Cicero, et al. (2012); Derosa et al. (2011) n‐3 PUFAs were also able to improve glucose metabolism. In an 18‐month clinical trial, the same authors evaluated the efficacy of this supplement on the glycemic profile in 281 overweight/obese subjects with IFG or IGT that received 3 g/day of supplement with content of EPA and DHA in the ratio 0.9–1.5. After 9 months and at the end of treatment it has been observed a significant decrease of FPG and HOMA‐IR both to baseline (at 9 months: −6.40 mg/dl, −5.50%, *p* < .05 for FPG and −0.90, −16.10%, *p* < .05 for HOMA‐IR; at 18 months: −13.30 mg/dl, −11.50%, *p* < .01 for FPG and −1.80, −32.10%, *p* < .01 for HOMA‐IR) and to placebo (at 9 months: −10.20 mg/dl, −8.50%, *p* < .05 for FPG and −1.10, −19.00%, *p* < .05 for HOMA‐IR; at 18 months: −19.50 mg/dl, −16.00%, *p* < .01 for FPG and −2.80, −42.40%, *p* < .01 for HOMA‐IR). Moreover, the supplement reduced FPI levels after 9 months (−2.30 μU/ml, −11.70%, *p* < .05) and 18 months (−4.40 μU/ml, −22.40%, *p* < .01) respect to baseline and at the end of treatment (−7.20 μU/ml, −32.10%, *p* < .05) compared to placebo (Derosa, Cicero, et al., 2016).

Jacobo‐Cejudo et al. (2017) also showed that the administration of 520 mg n‐3 PUFAs consisting of 320 mg EPA and 200 mg DHA for 6 months in 54 T2DM patients decreased FPG (−21.10 mg/dl, −11.90%, *p* = .011) and HbA_1c_ levels (−1.40%, −14.6%, *p* = .009) respect to baseline. The supplement also significantly increased FPI (+6.60 μU/ml, +86.80%, *p* = .000) and HOMA‐IR (+2.20, +71.00%, *p* = .000) by comparison to values before the n‐3 PUFAs intake.

As regard the glucose‐lowering activity of n‐3 PUFAs in association with other nutraceuticals it has been previously described the effect of fish oil capsules and curcumin tablets combination (Thota et al., 2019).

### 3.6 Psyllium [*Plantago ovata* (L) Pers]

Psyllium is derived from the seed of the *P. ovata* plant and it is also called Ispagula or Isabgol. Psyllium husk is a bulking fiber that, once ingested, forms a mucilaginous product in the colon by drawing in water and promotes so the correct intestinal transit. Since psyllium is less readily fermented it is better tolerated than the other fibers (Pal & Radavelli‐Bagatini, 2012). Generally, it is used as a laxative and it has been demonstrated the efficacy in treating some diseases such as irritable bowel syndrome, ulcerative colitis, colon cancer, hypercholesterolemia, obesity, and diabetes (Mishra, Sinha, Dey, & Sen, 2014). Among the many health benefits of psyllium, its glucose‐lowering effectiveness was taken into consideration.

#### 3.6.1 Mechanisms of action

**FIGURE 6:**
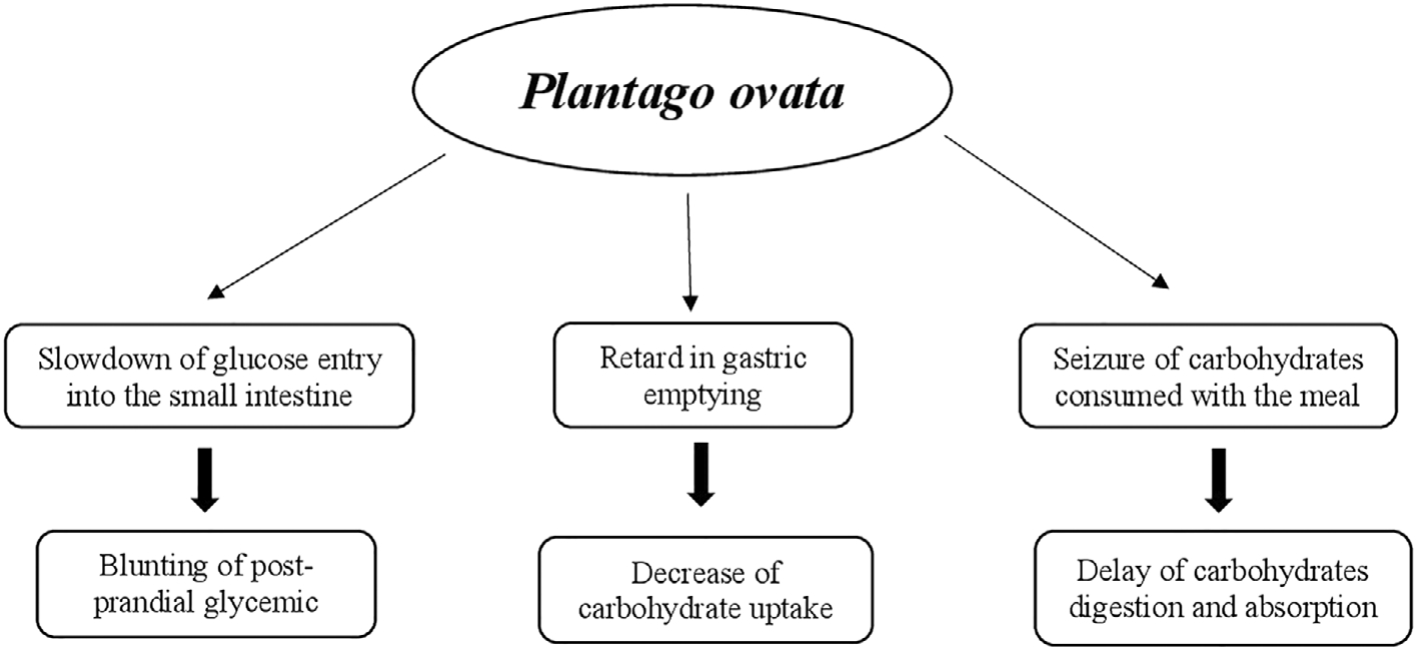
Potential glucose‐lowering mechanisms of *Plantago ovata*

The proposed hypoglycemic mechanisms of psyllium appear to be similar to those of other soluble fibers. They are represented by the slowed entry of glucose into the small intestine resulting in a blunting of post‐prandial glycemic peaks (C. A. Edwards, Johnson, & Read, 1988; D. J. Jenkins & Jenkins, 1985); retarded gastric emptying decreasing carbohydrate uptake (Holt, Heading, Carter, Prescott, & Tothill, 1979); seizure of carbohydrates consumed with the meal delaying their digestion and absorption (Dunaif & Schneeman, 1981) (Figure 6).

#### 3.6.2 Clinical trials

A large number of human studies investigated the hypoglycemic activity of psyllium (Table 6).

**TABLE 6:** Summary of clinical trials on *Plantago ovata* glycemic control

Subjects	Drug treatment and period	Results	References
T2DM patients (*n* = 12)	6.6 g psyllium *n* = 3 visit	PPG mean increment reduction	Sartor, Carlström, and Scherstén (1981)
T2DM non‐insulin dependent patients (*n* = 18)	13.6 g/day psyllium 15 hr with 7 days wash‐out	PPG decrease after breakfast and dinner Glucose AUC decrease after dinner PPI and insulin AUC decrease after breakfast	Pastors, Blaisdell, Balm, Asplin, and Pohl (1991)
T2DM patients (*n* = 125)	15.0 g/day psyllium 6 weeks	FPG decrease	Rodríguez‐Morán, Guerrero‐Romero, and Lazcano‐Burciaga (1998)
T2DM, hypercholesterolemia patients (*n* = 34)	10.2 g/day psyllium 8 weeks	All‐day and post‐lunch PPG decrease Not significant HbA_1c_ decrease	Anderson, Allgood, Turner, Oeltgen, and Daggy (1999)
Healthy subjects (*n* = 10)	10.5 g/day psyllium 2 days	Serum insulin decreased at 30, 60, and 90 min after OGTT Glucose and insulin AUC decrease	Sierra et al. (2001)
T2DM, overweight patients (*n* = 20)	14.0 g/day psyllium 6 weeks	Glucose and insulin AUC decrease Not significant HbA_1c_ and C‐peptide decrease	Sierra, García, Fernández, Diez, and Calle (2002)
T2DM patients (*n* = 49)	10.2 g/day psyllium 8 weeks	FPG and HbA_1c_ decrease Not significant insulin decrease	Ziai et al. (2005)
T2DM patients (*n* = 12)	5.0 g psyllium 1 day	2 hr‐PPG decrease Difference between 1‐hr PPG and FPG values decrease	Dastjerdi, Salehioun, Najafian, and Amini (2007)
Hypertension, overweight patients (*n* = 141)	10.5 g/day psyllium or PHGG 6 months	FPG, FPI, HbA_1c_, and HOMA‐IR decrease	Cicero et al. (2007)
Metabolic syndrome patients (*n* = 141)	10.5 g/day psyllium or PHGG 6 months	FPG, FPI, HbA_1c_, and HOMA‐IR decrease	Cicero, Derosa, Di Gregori, et al. (2010); Cicero, Derosa, Bove, et al. (2010)
T2DM patients (*n* = 40)	10.5 g/day psyllium 2 months	FPG and HbA_1c_ decrease	Sartore et al. (2009)
T2DM patients (*n* = 37)	6.8 g/day psyllium 12 weeks 2 13.6 g/day psyllium 12 weeks	FPG and HbA_1c_ decrease	Feinglos, Gibb, Ramsey, Surwit, and McRorie (2013)
T2DM patients (*n* = 36)	10.5 g/day psyllium 8 weeks	FPG, FPI, HbA_1c_, C‐peptide, HOMA‐IR, and HOMA‐B improvement	Abutair, Naser, and Hamed (2016)
Overweight, obesity patients (*n* = 159)	15.0 g/day psyllium 12 months	FPI and HOMA‐IR decrease	Pal, Ho, Gahler, and Wood (2017)
T2DM, overweight, obesity patients (*n* = 37)	7.0 g/day psyllium 2 weeks	FPI and HOMA‐IR decrease	Kamalpour, Ghalandari, and Nasrollahzadeh (2018)
T2DM patients (*n* = 51)	20.0 g/day psyllium 12 weeks	FPG and HbA_1c_ decrease	Noureddin, Mohsen, and Payman (2018)
T2DM patients (*n* = 30)	12.8 g/day of mixture (2.0 g psyllium + 600 mg *Allium sativum* + 600 mg *Aloe vera* + 3.6 g *Nigella sativa* + 1.0 g *Silybum marianum* + 5.0 g *Trigonella faenum graecum*) 40 days	FPG and HbA_1c_ decrease	Zarvandi, Rakhshandeh, Abazari, Shafiee‐Nick, and Ghorbani (2017)

Abbreviations: AUC, area under the curve; FPG, fasting plasma glucose; FPI, fasting plasma insulin; HbA_1c_, glycated hemoglobin; HOMA‐B, homeostatic model assessment of β‐cell function; HOMA‐IR, homeostatic model assessment of insulin resistance; HOMA index, homeostatic model assessment index; OGTT, oral glucose tolerance test; PHGG, partially hydrolyzed guar gum; PPG, post‐prandial plasma glucose; PPI, post‐prandial plasma insulin; T2DM, type 2 diabetes mellitus.

A short‐duration study showed that 12 T2DM patients supplemented with 6.6 g of psyllium added to a standardized breakfast during 3 occasions spaced at least 1 week apart exhibited a significant reduction of the mean increment PPG (−9.00%, *p* < .05) (Sartor et al., 1981).

Pastors et al. (1991) reported that the administration of psyllium at the dose of 13.6 g before breakfast and dinner in 18 T2DM patients significantly reduced the peak PPG levels at both meals (−17.66 mg/dl, − 14.00%, *p* = .08 after breakfast; −14.24 mg/dl, − 21.00%, *p* = .06 after dinner) and the glucose AUC after dinner (−3.40 mmol hr^−1^ L^−1^, −41.00%, *p* = .07) relative to placebo. The supplement also decreased the maximum PPI elevation (−9.51 μIU/ml, −12.00%, *p* = .09) and the insulin AUC (−24.80 μIU hr^−1^ ml^−1^, −17.00%, *p* = .02) after breakfast compared to placebo. In addition, residual psyllium taken 5 hr before breakfast reduced PPG peak values (−20.72 mg/dl, −31.00%, *p* = .01) and glucose AUC (−88.47 mg hr^−1^ dl^−1^, −64.00%, *p* = .02) after lunch where the supplement was not taken respect to placebo (Pastors et al., 1991).

In the study of Rodríguez‐Morán et al. (1998) 125 T2DM patients ingested 15 g/day of psyllium or placebo before regular meals in addition to a low‐fat diet. After 6 weeks of treatment, the supplement significantly decreased FPG levels (−52.50 mg/dl, −27.30%, *p* < .01) compared to baseline values (Rodríguez‐Morán et al., 1998).

Another study described the effect of psyllium 10.2 g/day given 20–30 min before breakfast and dinner to 34 men with T2DM and hypercholesterolemia. After an 8‐week treatment period, it has been observed that the supplement produced a significant decrease in all‐day and post‐lunch PPG levels both respect to baseline (−9.01 mg/dl, −4.20%, *p* < .05 for all‐day PPG; −12.61 mg/dl, −6.50%, *p* = .01 for post‐lunch PPG) and to placebo (−16.40 mg/dl, −7.40%, *p* < .05 for all‐day PPG; −30.63 mg/dl, −14.50%, *p* = .01 for post‐lunch PPG). Psyllium also reduced HbA_1c_ values compared to baseline (−0.46%, −6.30%) and to placebo (−0.60%, −8.10%) although not significantly (Anderson et al., 1999).

Sierra et al. (2001) showed that 10.5 g of psyllium added to an oral glucose load administered for 2 days in 10 healthy females significantly reduced serum insulin levels at 30 min (−22.01 μU/ml, −32.50%, *p* < .05), 60 min (−34.59 μU/ml, −41.00%, *p* < .05), and 90 min (−39.77 μU/ml, −49.70%, *p* < .05) respect to glucose load without supplement. In addition, the glucose and insulin AUC were reduced by 11.0 and 36.10%, respectively (*p* < .05 for both) (Sierra et al., 2001).

The same authors evaluated the effect of psyllium administration at the dose of 14 g/day before meals for 6 weeks in 20 T2DM overweight subjects treated with diet and sulphonylureas. According to the study protocol, the treatment (phase 2) was preceded by a 1‐week (phase 1) and followed by a 2‐week wash‐out and 4‐week period (phase 3) in which the patients did not receive the supplement. It has been shown that psyllium decreased the glucose AUC by 12.20 and 11.90% compared to phases 1 and 3, respectively (*p* < .05 for both). The supplement also lowered the insulin AUC by 5 and 15% respect to the value of phases 1 and 3 (*p* < .05 for both). In addition, the treatment reduced HbA_1c_ and C‐peptide (HbA_1c_: −3.80 and −5.50% compared to phases 1 and 3; C‐peptide: −14.90 and 10.00% respect to phase 1 and 3) though not significantly (Sierra et al., 2002).

Similarly, to the Anderson et al. (1999), Ziai et al. (2005) showed that the assumption of 10.2 g/day of psyllium 20–30 min before breakfast and dinner for 8 weeks in 49 T2DM patients determined a significant reduction of FPG (−28.00 mg/dl, −60.60%, *p* < .05) and HbA_1c_ levels (−1.60%, −15.20%, *p* < .05) without any change of insulin values by comparison to placebo (Ziai et al., 2005).

Another short‐term trial evaluated the effect of psyllium granules on the glycemic profile in 12 T2DM subjects treated with diet. The supplement added to breakfast at the dose of 5.0 g produced a significant reduction of 2 hr‐PPG levels (−50.00 mg/dl, −29.80%, *p* = .05) as well as a difference between 1 hr‐PPG and FPG values (−66.90 mg/dl, 36.00%, *p* = .037) compared to control group (Dastjerdi et al., 2007).

The effect of psyllium or PHGG administration in 141 patients with hypertension and overweight or with metabolic syndrome on glycemic parameters has been previously described (Cicero et al., 2007; Cicero, Derosa, Bove, et al., 2010; Cicero, Derosa, Di Gregori, et al., 2010).

One trial reported that 10.5 g/day of psyllium administered before breakfast, lunch, and dinner for 2 months in 40 T2DM patients treated with a controlled diet and sulfonylureas significantly decreased FPG (−4.83 mg/dl, −3.40%, *p* < .05) and HbA_1c_ levels (−0.20%, −2.90%, *p* < .05) respect to baseline (Sartore et al., 2009).

Feinglos et al. (2013) evaluated the dose–response efficacy of psyllium on the glycemic profile in 37 T2DM patients, already treated with diet and oral hypoglycemic agents, that received 6.8 or 13.6 g/day of supplement just before breakfast and dinner for a 12‐week period. Both psyllium doses significantly reduced FPG levels at week 4 (−46.04 mg/dl, −19.90%, *p* = .012 for 6.8 g/day of supplement; −69.49 mg/dl, −30.00%, *p* = .005 for 13.6 g/day of supplement), week 8 (−36.94 mg/dl, −16.10%, *p* = .028 for 6.8 g/day of supplement; −57.20 mg/dl, −25.00%, *p* = .018 for 13.6 g/day of supplement), and week 12 (−41.44 mg/dl, −17.60%, *p* = .028 for 6.8 g/day of supplement; −67.02 mg/dl, −28.50%, *p* = .009 for 13.6 g/day of supplement) respect to placebo. In addition, the supplement decreased HbA_1c_ values after 8 weeks of treatment with a 13.6 g/day dose (−0.58%, −7.40%, *p* = .003) and after 12 weeks with both dosages (−0.73%, −9.30%, *p* = .013 for 6.8 g/day of supplement; −0.65%, −8.30%, *p* = .003 for 13.6 g/day of supplement) compared to placebo (Feinglos et al., 2013).

In the study by Abutair et al. (2016), 36 T2DM patients received 10.5 g/day of psyllium 15 min before lunch and dinner for an 8‐week period. At the end of intervention, the supplement significantly improved FPG (−43.00 mg/dl, −26.40%, *p* < .001), FPI (−8.20 μIU/ml, −29.40%, *p* < .001), HbA_1c_ (−1.00%, −11.80%, *p* < .001), C‐peptide (−2.00 ng/ml, −34.50%, *p* < .001), HOMA‐IR (−5.50, −48.70%, *p* < .001), and HOMA‐B (+37.80%, +36.70%, *p* < .01) compared to baseline (Abutair et al., 2016).

In a 12‐month trial were enrolled 159 overweight and obese subjects were divided into three groups treated with 15 g/day of psyllium, a complexed polysaccharide or placebo respectively ingested 5–10 min before meals. It has been observed that psyllium significantly decreased FPI levels at 3 months (−5.50%, *p* = .032) respect to baseline while at 3 months (−9.40%, *p* = .004), 6 months (−9.10%, *p* = .040), and 12 months (−9.40%, *p* = .029) compared to control. In addition, in the group supplemented with psyllium was found a significant decrease of HOMA‐IR at 3 months (−7.00%, *p* = .011) and 6 months (−6.70%, *p* = .037) respect to baseline whereas at 3 months (−10.80%, *p* = .001), 6 months (−11.90%, *p* = .018), and 12 months (−11.00%, *p* = .011) compared to control (Pal et al., 2017).

A short‐term study evaluated the effect of psyllium added to a moderate carbohydrate diet or placebo added to a lower carbohydrate diet on glycemic control in 37 T2DM overweight and obese patients. Psyllium was given at the dose of 7 g/day for 2 weeks. At the end of supplementation, the group treated with a moderate carbohydrate diet plus psyllium showed a significant lowering of FPI levels (−0.87 μIU/ml, −20.70%, *p* = .01) and HOMA‐IR (−0.30, −21.70%, *p* = .016) respect to baseline values (Kamalpour et al., 2018).

Another trial showed that among 51 T2DM subjects with chronic constipation daily taking 20.0 g of psyllium or placebo for 12 weeks, the group treated with the supplement exhibited a significant lowered FPG both to baseline (−19.50 mg/dl, −11.40%, *p* < .05) and to placebo (−12.20 mg/dl, −7.50%, *p* < .05) and HbA_1c_ (−1.40%, −15.60%, *p* < .01) respect to placebo (Noureddin et al., 2018).

The hypoglycemic action of psyllium in combination with other nutraceuticals was also investigated. Zarvandi et al. (2017) evaluated the efficacy of *Plantago psyllium* combined with *Allium sativum*, *Aloe vera*, *Nigella sativa*, *S. marianum*, and *T. faenum graecum* on glucose metabolism in 30 advanced stage T2DM patients treated with oral hypoglycemic drugs. Patients received 12.8 g/day of nutraceuticals mixture composed of 2.0 g *P. psyllium*, 600 mg *A. sativum*, 600 mg *A. vera*, 3.6 g *N. sativa*, 1.0 g *S. marianum*, and 5.0 g *T. foenum‐graecum*. After 40 days of treatment, the supplement reduced FPG (−16.00 mg/dl, −9.90%, *p* < .05) and HbA_1c_ (−0.70%, −8.30%, *p* < .05) levels respect to baseline values. These findings demonstrated that the glucose‐lowering effect of psyllium is also due to the synergistic action of all nutraceuticals of the mixture whose individual hypoglycemic action has been proven (Zarvandi et al., 2017).

### 3.7 Quercetin [*Allium cepa* (L) Pers]

Quercetin is an important member of the flavonoids family and comes from various vegetables and fruits including onion, apple, berries, many nuts, seeds, barks, flowers, tea, brassica vegetables, and leaves (C. Chen, Zhou, & Ji, 2010). Recent studies showed that quercetin may exert different biological activities on human health, such as cardiovascular prevention, antiallergic, anticancer, antiinflammatory, antibacterial, antiviral, analgesic, and antidiabetes action (E. Y. Choi et al., 2015; H. N. Choi, Jeong, Huh, & Kim, 2015; A. Gupta, Birhman, Raheja, Sharma, & Kar, 2016). Quercetin has been extensively studied as a potential herbal alternative treatment for diabetes management (Shi et al., 2019).

#### 3.7.1 Mechanisms of action

**FIGURE 7:**
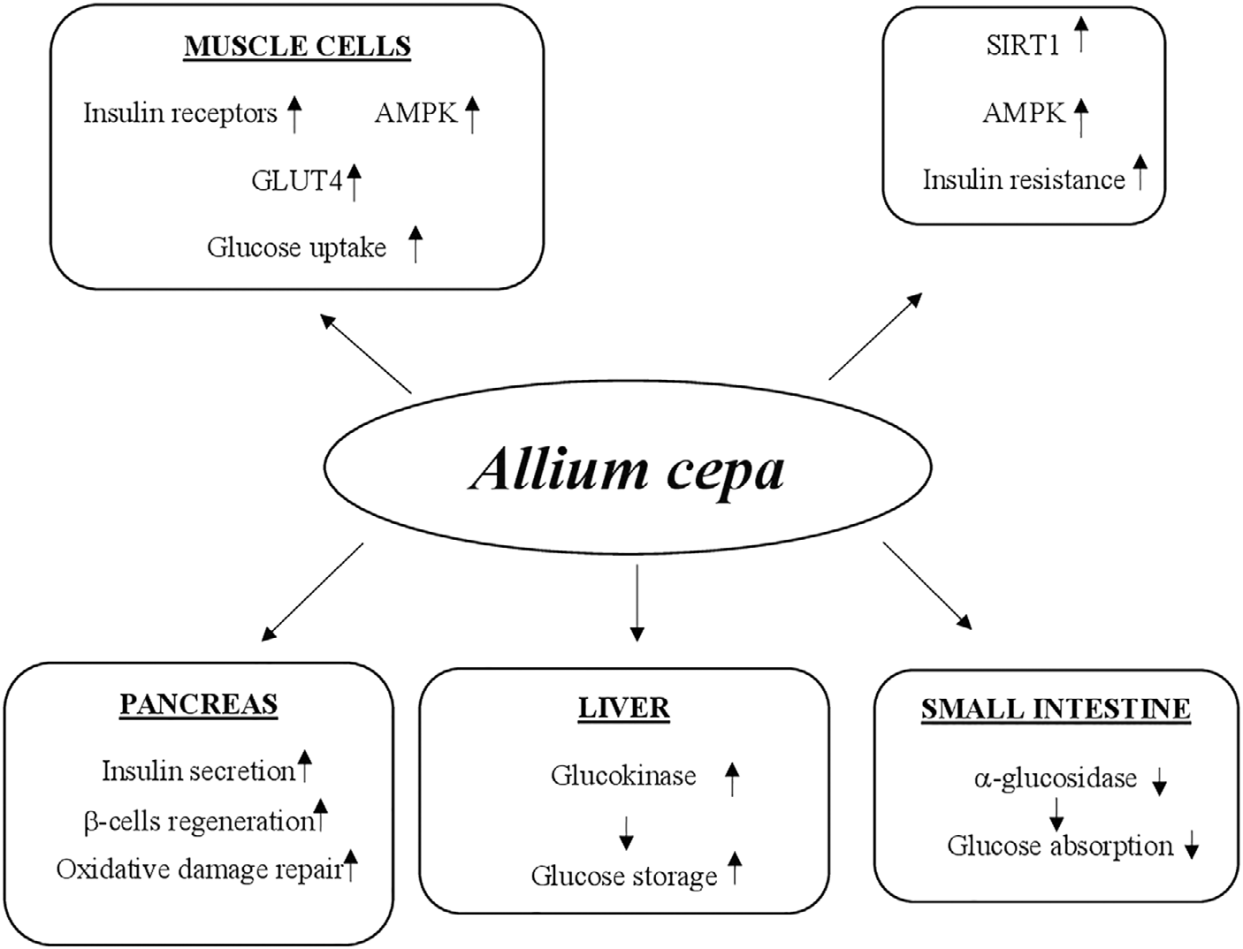
Potential mechanisms for hypoglycemic effect of *Allium cepa*. AMPK, adenosine monophospate‐activated protein kinase; GLUT4, glucose transporter 4; SIRT1, silent information regulator 1

Many studies mainly performed on diabetic rat models showed both hypoglycemic properties of quercetin and related possible mechanisms of action. This nutraceutical acts on the pancreas, liver, muscle, and small intestine to normalize blood sugar levels. As regard pancreas, the quercetin action on islet β‐cells consists of an increase in insulin secretion, protection of β‐cells from oxidative damage, and enhancement of their proliferation. This nutraceutical increases glucokinase activity enhancing glucose storage in the liver. Quercetin is able to promote glucose uptake in skeletal muscle cells under the condition of insulin absence due to raised glucose transporter 4 (GLUT4) expression in the plasma membrane. The endogenous GLUT4 translocation is stimulated by a signaling pathway involving insulin receptor tyrosine kinase or AMPK activation. Quercetin also inhibits α‐glucosidase activity to decrease intestinal glucose absorption. In addition, the nutraceutical improves insulin resistance and decreases glucose production in hepatocytes by raising silent information regulator 1 (SIRT1) protein expression and activating AMPK in duodenal mucosa (Shi et al., 2019) (Figure 7).

#### 3.7.2 Bioavailability

Quercetin is characterized by poor oral bioavailability mainly due to sugar moiety (Hussain, Ahmed, Mahwi, & Aziz, 2012). This molecule is found in plants as hydrophilic glycosides which are not easily absorbed. However, following hydrolysis of glycosides the absorption of quercetin in aglycone form can reach up to 65–81% (Shi et al., 2019). Recently has been developed a novel formulation of quercetin based on food‐grade lecithin, Quercetin Phytosome and it has been observed that its assumption allowed to reach quercetin plasma levels up to 20 times higher than those obtained after administering unformulated quercetin in healthy volunteers. Then, the incorporation into phytosomes led to an increase in quercetin solubility, and thus to an improvement of its bioavailability as well as biological activity (Riva, Ronchi, Petrangolini, Bosisio, & Allegrini, 2019).

#### 3.7.3 Clinical trials

Some clinical trials evaluated the effects of quercetin on glycemic control and conflicting results were reported (Table 7).

**TABLE 7:** Summary of human studies on hypoglycemic effect of *Allium cepa*

Subjects	Drug treatment and period	Results	References
Prehypertension patients (*n* = 19) Stage 1 hypertension patients (*n* = 22)	730 mg/day quercetin aglycone 4 weeks	FPG no effect	R. L. Edwards et al. (2007)
Overweight, obesity with MS traits patients (*n* = 93)	150 mg/day quercetin 6 weeks	FPG no effect	Egert et al. (2009)
Type 1 and 2 DM patients (*n* = 84)	100 g *A. cepa*	FPG and hyperglycemic peak decrease at 4 hr after GTT	Taj Eldin, Ahmed, and Elwahab (2010)
T2DM patients (*n* = 46)	250 mg/day quercetin 8 weeks	FPG, FPI and HbA_1c_ no effect	Mazloom, Abdollahzadeh, Dabbaghmanesh, and Rezaianzadeh (2014)
Overweight, obesity patients (*n* = 72)	100 mg/day quercetin 12 weeks	FPG no effect	E. Y. Choi, Lee, et al. (2015); H. N. Choi, Jeong, et al. (2015)
Lactose‐tolerance and intolerance patients (*n* = 24)	Onion meal (2.0 μg/ml quercetin, 39.5 μg/ml quercetin‐4′‐O‐glucoside, 3.5 μg/ml quercetin‐3‐O‐glucoside, 26.5 μg/ml quercetin‐3,4′‐O‐diglucoside)	Peak glucose and iAUC decrease greater in lactose‐intolerant subjects than lactose‐tolerant ones after GTT	Hoffman, Ranjbar, and Madden (2016)
Overweight, obesity patients (*n* = 72)	100 mg/day quercetin 12 weeks	FPG increase; FPI no effect	Lee, Cha, Lee, and Yim (2016)
Breast cancer patients (*n* = 56)	100–160 g/day (HO group) 30–40 g/day (LO group) 8 weeks	FPG, FPI and HOMA IR decrease in HO group	Jafarpour‐Sadegh et al. (2017)
Overweight, obesity, prehypertension, stage 1 hypertension patients (*n* = 68)	162 mg/day quercetin 6 weeks	FPG, FPI, HbA_1c_ and HOMA‐IR no effect	Brüll et al. (2017)
PCOS patients (*n* = 82)	1 g/day quercetin 12 weeks	FPG, FPI and HOMA IR decrease	Rezvan et al. (2017) Rezvan, Moini, Gorgani‐Firuzjaee, and Hosseinzadeh‐Attar (2018)
Overweight, obesity, PCOS patients (*n* = 78)	1 g/day quercetin 12 weeks	FPG, FPI and HOMA IR decrease	Khorshidi et al. (2018)

Abbreviations: FPG, fasting plasma glucose; FPI, fasting plasma insulin; GTT, glucose tolerance test; HbA_1c_, glycated hemoglobin; HO, high onion; HOMA‐IR, homeostatic model assessment of insulin resistance; iAUC, incremental area under curve; LO, low onion; MS, metabolic syndrome; PCOS, polycystic ovary syndrome; T2DM, type 2 diabetes mellitus.

In the study by R. L. Edwards et al. (2007), 19 prehypertensive and 22 stage 1 hypertensive subjects took 730 mg/day of quercetin aglycone or placebo for 4 weeks. At the end of treatment, it has been observed that the supplement did not alter FPG levels (R. L. Edwards et al., 2007).

Similarly, Egert et al. (2009) reported that the administration of 150 mg/day quercetin in 93 overweight or obese patients with metabolic syndrome traits for 6 weeks did not affect FPG levels (Egert et al., 2009).

One trial, instead, evaluated the hypoglycemic effect of red onion (*A. cepa*), one of the main sources of quercetin, in 84 types 1 and 2 diabetic patients subjected to glucose tolerance test (GTT) consisting of the administration of 75 g dextrose orally. All patients were divided into subgroups: those of type 1 diabetics received water 5 ml, insulin 5 IU/prescribed dose, or crude fresh slices *A. cepa* 100 g whereas those of T2DM subjects taken water 5 ml, glibenclamide 5 mg, or crude fresh slices *A. cepa* 100 g. FPG and GGT were measured at 0, 1, 2, and 4 hr. It has been observed that the supplement significantly reduced FPG (−88.62 mg/dl, −38.20%, *p* = .005) compared to the decrease produced by insulin (−144.33 mg/dl, −59.40%, *p* = .001) in type 1 diabetics and it lowered FPG (−39.50 mg/dl, −25.50%, *p* = .005) in relation to glibenclamide (−80.62 mg/dl, −44.50%, *p* = .003) in T2DM patients, after 4 hr. In addition, the supplement caused a significant reduction in the induced hyperglycemia (−120.60 mg/dl, −30.20%, *p* = .004) compared to insulin (−151.66 mg/dl, −49.80%, *p* = .001) in type 1 diabetics and it significantly decreased GTT (−159.50 mg/dl, −42.80%, *p* = .002) respect to glibenclamide (−113.80 mg/dl, −41.10%, *p* = .001) in T2DM patients, 4 hr later (Taj Eldin et al., 2010).

However, Mazloom et al. (2014) showed that in 46 T2DM patients that received 250 mg/day of quercetin or placebo for 8 weeks, the supplement did not change FPG, FPI, and HbA_1c_ levels (Mazloom et al., 2014).

E. Y. Choi, Lee, et al. (2015) and H. N. Choi, Jeong, et al. (2015) also reported that the ingestion of 100 mg/day quercetin or placebo in 72 overweight and obese subjects for 12 weeks did not affect FPG levels after supplementation (H. N. Choi, Jeong, et al., 2015; E. Y. Choi, Lee, et al., 2015).

In another study, 24 lactose‐tolerant and lactose‐intolerant individuals were subjected to GTT receiving glucose alone or glucose plus onion meal that contained 2.0 μg/ml quercetin, 39.5 μg/ml quercetin‐4′‐O‐glucoside, 3.5 μg/ml quercetin‐3‐O‐glucoside, and 26.5 μg/ml quercetin‐3,4′‐O‐diglucoside. It has been observed that onion meal produced a greater decrease in peak glucose levels and iAUC of glucose in lactose‐intolerant (−44.19%, *p* = .042 for peak blood glucose; −54.53% *p* = .42 for iAUC) than in lactose‐tolerant subjects (−19.28% for peak blood glucose; −42.06% for iAUC). These results suggest that subjects who cannot tolerate lactose probably retain intact quercetin glucosides that can inhibit glucose uptake while lactose‐tolerant people hydrolyze it to free quercetin that does not inhibit glucose uptake (Hoffman et al., 2016).

A 12‐week study including 72 overweight and obese subjects given 100 mg/day of quercetin or placebo showed that the supplement significantly increased FPG (+3.30 mg/dl, +3.40%, *p* = .04) and did not affect FPI levels (Lee et al., 2016).

Jafarpour‐Sadegh et al. (2017) evaluated the effect of raw yellow onions intake on glycemic control in 56 breast cancer patients treated with doxorubicin. These subjects received a body mass index (BMI)‐dependent number of yellow onions in addition to main meals for 8 weeks. Participants in the high onion (HO) group took 100 to 160 g/day of the product whereas those in the low onion (LO) group received 30–40 g/day. It has been observed that patients in HO group exhibited a significant decrease in FPG (−11.50 mg/dl, −10.80%, *p* = .014) and HOMA‐IR (−0.006, −11.50%, *p* = .021) compared to baseline as well as FPI (−0.01 ng/ml, −4.80%, *p* = .008) respect to LO group (Jafarpour‐Sadegh et al., 2017).

On the contrary, one trial reported that in a sample of 68 overweight and obese patients with pre‐hypertension and stage 1 hypertension that ingested 162 mg/day of quercetin or placebo, the supplement did not modify FPG, FPI, HbA1c, and HOMA‐IR values after a treatment period of 6 weeks (Brüll et al., 2017).

Other studies have shown a positive effect of quercetin on glucose homeostasis when it has been administered at higher doses than those used in the studies described above. In this regard, Rezvan et al. (2017, 2018) evaluated the efficacy of quercetin administration in 82 women with polycystic ovary syndrome (PCOS) treated with 1 g/day of supplement or placebo for 12 weeks. It was found that quercetin significantly reduced FPG levels (−1.21 mg/dl, −1.30%, *p* < .001) respect to baseline and FPI as well as HOMA‐IR values compared both to baseline (−1.64 μU/ml, −16.30%, *p* < .001 for FPI; −0.40, −17.50%, *p* < .001 for HOMA‐IR) and to placebo (−1.36 μU/ml, −13.90%, *p* = .013 for FPI; −0.29, −13.40%, *p* = .039 for HOMA‐IR) (Rezvan et al., 2017, 2018).

Similarly, Khorshidi et al. (2018) reported that 78 overweight or obese women with PCOS randomized to receive 1 g/day quercetin or placebo for 12 weeks presented a significant lowering in FPG (−1.20 mg/dl, −1.30%, *p* < .001), FPI (−1.50 μU/ml, −7.70%, *p* = .02), and HOMA‐IR (−0.30, −7.00%, *p* = .009) levels at the end of supplementation (Khorshidi et al., 2018).

### 3.8 *Stevia rebaudiana* (L) Pers

*S. rebaudiana* is a perennial herb that belongs to the Asteraceae family native to South America, particularly Paraguay and Brasil. In many countries, extracts from leaves of this plant are used as a zero‐calorie sweetener in a variety of products including foods and beverages as well as for their pharmacological actions comprising anticancer, antihypertensive, hypoglycemic, and antidiarrheal activity. Steviol glycosides are natural compounds of *S. rebaudiana* leaves responsible for the sweet taste and they are represented by stevioside (5–10%), rebaudioside A (2–4%), rebaudioside C (1–2%), and dulcoside A (0.5–1%). There are also rebaudioside B, D, and E that are present in very low amounts. *S. rebaudiana* also resulted in the richest source of steviol glycosides among the other species belonging to the genus Stevia. Stevioside and rebaudioside A, besides being the most abundant glycosides in the *S. rebaudiana* leaves, sweeten about 200–300 times more than 0.4 M sucrose. Steviol glycosides have been widely investigated for their potential hypoglycemic activity (Brahmachari, Mandal, Roy, Mondal, & Brahmachari, 2011; Bundgaard Anker, Rafiq, & Jeppesen, 2019).

#### 3.8.1 Mechanisms of action

**FIGURE 8:**
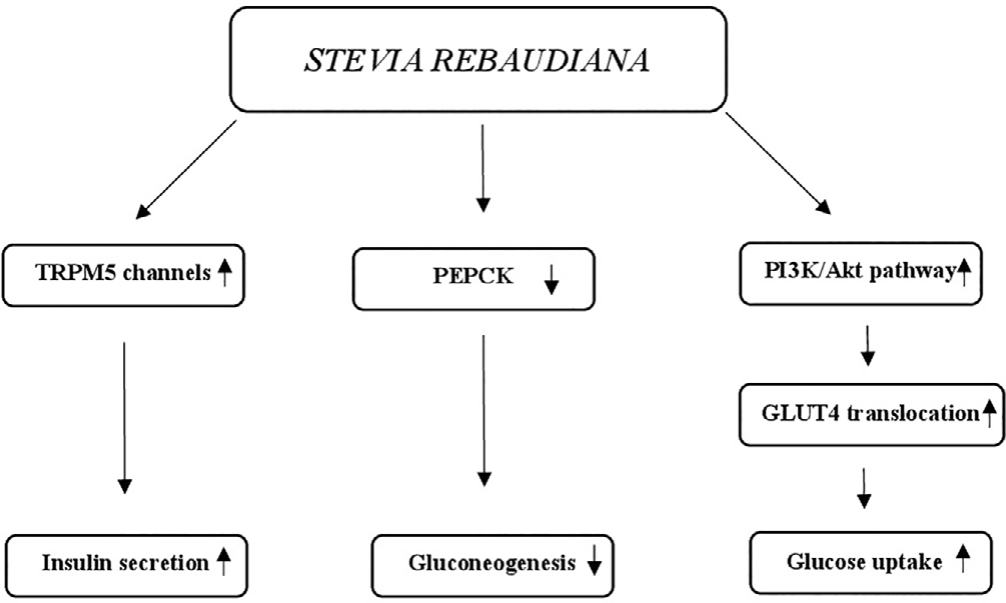
Hypoglycemic effects of *Stevia rebaudiana* extracts. Akt, serine/threonine kinase; GLUT4, glucose transporter 4; PEPCK, phosphoenolpyruvate carboxykinase; PI3K, phosphatidylinositol 3‐kinase; TRPM5, transient receptor potential cation channel subfamily melastin 5

In vivo and in vitro studies investigated the mechanisms that underlie the hypoglycemic action of steviol glycosides. It has been reported that the *S. rebaudiana* leaves extracts to increase insulin secretion by interacting with transient receptor potential cation channel subfamily melastin 5 (TRPM5), a monovalent Ca^2+^‐activated cation channel found in pancreatic β‐cells and involved in the perception of sweet, bitter, and umami tastes as well as in insulin secretion. Steviol glycosides also prolong TRPM5 channel activation thus increasing insulin release. Moreover, *S. rebaudiana* extracts exert a hypoglycemic effect by inhibiting of PEPCK gene expression cause a gluconeogenesis slowdown. In addition, steviol glycosides are able to enhance glucose uptake increasing GLUT4 translocation by activation of phosphatidylinositol 3‐kinase/serine/threonine kinase (PI3K/Akt) pathway (Ray et al., 2020). (Figure 8).

#### 3.8.2 Clinical trials

A number of human studies have been performed to explore the hypoglycemic effects of *S. rebaudiana* extracts (Table 8).

**TABLE 8:** Summary of human studies on hypoglycemic effect of *Stevia rebaudiana* extracts

Subjects	Drug treatment and period	Results	References
Normal (*n* = 16)	20 g/day aqueous extracts of *S. rebaudiana* leaves 3 days	FPG and PPG decrease	Curi et al. (1986)
Mild to moderate hypertension patients (*n* = 100)	750 mg/day stevioside 12 months	FPG no effect	Chan et al. (2000)
Mild hypertension patients (*n* = 168)	1,500 mg/day stevioside 24 months	FPG no effect	Hsieh et al. (2003)
T2DM patients (*n* = 12)	1 g/day stevioside 2 days	iAUC decrease. Insulinogenic index increase	Gregersen, Jeppesen, Holst, and Hermansen (2004)
T2DM patients (*n* = 55)	1,500 mg/day stevioside 3 months	FPG and HbA_1c_ no effect	Jeppesen et al. (2006)
Mild untreated hypertension patients (*n* = 14)	3.75 mg kg^−1^ day^−1^ stevioside 7 weeks 2 7.50 mg kg^−1^ day^−1^ stevioside 11 weeks 3 15.00 mg kg^−1^ day^−1^ 6 weeks	FPG, FPI and HOMA‐IR decrease	Ferri et al. (2006)
Untreated hyperlipidemia patients (*n* = 43)	200 mg/day stevioside 3 months	FPG no effect	Da Silva et al. (2006)
Subjects with or without type 1 and 2 diabetes (*n* = 76)	750 mg/day stevioside 3 months	FPG and HbA_1c_ no effect	Barriocanal et al. (2008)
T2DM patients (*n* = 122)	1 g/day rebaudioside A 16 weeks	FPG, FPI, HbA_1c_ and C‐peptide no effect	Maki et al. (2008)
Lean and obese subjects (*n* = 31)	Preload with *Stevia* Aspartame Sucrose 3 days	PPG and PPI decrease	Anton et al. (2010)
T2DM patients (*n* = 20)	1 g/day *stevia* leaf powder 2 months	FPG and PPG decrease	Ritu and Nandini (2016)
Volunteers (*n* = 30)	Preload with 1 g/day *stevia* 60 g/day sugar Water 3 days	PPG no effect	Farhat, Berset, and Moore (2019)
T2DM patients (*n* = 34)	Black tea with 2% *stevia* extract 8 weeks	FPG, 2 hr‐PPG, FPI, and HbA_1c_ no effect	Ajami et al. (2020)
Prediabetes patients (*n* = 24)	10 g/day rosella powder 250 mg/day *stevia* 14 days	FPG decrease 2 hr‐PPG no effect	Mayasari, Susetyowati, Wahyuningsih, and Probosuseno (2018)

Abbreviations: FPG, fasting plasma glucose; FPI, fasting plasma insulin; HbA_1c_, glycated hemoglobin; HOMA‐IR, homeostatic model assessment of insulin resistance; iAUC, incremental area under curve; PPG, post‐prandial glucose; PPI, post‐prandial insulin; T2DM, type 2 diabetes mellitus.

An early short‐term trial by Curi et al. (1986) reported that the daily intake of aqueous extracts of *S. rebaudiana* leaves 20 g for 3 days in 16 normal adults subjects who undergone GTT, significantly reduced FPG (−10.00 mg/dl, −11.10%, *p* < .01) and PPG levels at each time during the test compared to control. The maximal decrease of PPG was achieved at 30 min (−25.00 mg/dl, −19.60%, *p* < .01) after GTT.

Afterward, long‐term studies were performed and in one of them, including 100 mild to moderate hypertensive subjects given stevioside 750 mg/day or placebo for 12 months, it has been observed that the supplement did not affect FPG levels (Chan et al., 2000).

Similarly, Hsieh et al. (2003) showed that the oral administration of stevioside 1,500 mg/day or placebo in 168 mild essential hypertensive patients for 24 months did not alter FPG values after supplementation (Hsieh et al., 2003).

However, in an acute paired cross‐over study enrolling 12 T2DM patients that received a standard test meal added with 1 g of stevioside on 2 days separated by at least 1 week, the supplement significantly decreased the iAUC of glucose (−116.00 mmol/L × 240 min, −18.20%, *p* < .004) and increased the insulinogenic index (+24.00, +40.00%, *p* < .001) compared to control (Gregersen et al., 2004).

In accordance with previous long‐term trials, Jeppesen et al. (2006) reported that the administration of stevioside 1,500 mg/day in 55 T2DM subjects for 3 months did not modify both FPG and HbA_1c_ levels respect to baseline whereas these parameters were significantly increased after placebo (Jeppesen et al., 2006).

Instead, a sample of 14 untreated mild‐hypertensive subjects given crude stevioside at the dose of 3.75 mg kg^−1^ day^−1^ for 7 weeks, 7.50 mg kg^−1^ day^−1^ for 11 weeks, and 15.00 mg kg^−1^ day^−1^ for 6 weeks or placebo for 24 weeks, exhibited a significant lowering of FPG (−11.10 mg/dl, −12.10%, *p* < .05), FPI (−5.00 μU/L, −40.30%, *p* < .05), and HOMA‐IR (−1.50, −55.60%, *p* < .05) values respect to baseline following supplementation (Ferri et al., 2006).

On the contrary, in the study by da Silva et al., it has been shown that the intake of stevioside 200 mg/day or placebo in 43 untreated hyperlipidemic patients for 90 days did not alter FPG levels after supplementation (Da Silva et al., 2006).

Similar results were obtained in another study where 76 subjects with or without type 1 and T2DM were treated with stevioside 750 mg/day or placebo for a period of 3 months. At the end of treatment with *S. rebaudiana* extract, FPG and HbA_1c_ levels were not different from baseline, though in the placebo type 1 diabetics group was recorded a significant increase in FPG values compared to baseline (Barriocanal et al., 2008).

One study evaluated the administration of rebaudioside A 1 g/day or placebo in 122 T2DM subjects for 16 weeks demonstrating that the supplement did not alter FPG, FPI, HbA1c, and C‐peptide levels (Maki et al., 2008).

Anton et al. (2010) observed the effect of preloads containing *Stevia*, aspartame, or sucrose on PPG and PPI in 31 lean and obese subjects. The preloads were ingested before lunch and dinner for a period of 3 days. In the *Stevia* condition was found a significant decrease in PPG levels at 20 min after preload consumption and at 30 min after the test lunch meal with respect to sucrose (−20.70 mg/dl, −17.10%, *p* < .05 for 20 min post preload; −3.85 mg/dl, −3.80%, *p* < .05 for 30 min post lunch) and aspartame condition (−2.80 mg/dl, −2.70%, *p* < .05 for 20 min post preload; −4.55 mg/dl, −4.40%, *p* < .05 for 30 min post lunch). Post‐prandial glucose was also reduced at 60 min after the test lunch meal (−2.80 mg/dl, −2.80%, *p* < .05) compared to the aspartame condition. Post‐prandial insulin values were decreased in *Stevia* condition at 30 and 60 min after the test lunch meal compared to sucrose (−10.00 IU/kg, −16.70%, *p* < .05 for 30 min post lunch; −12.00 IU/kg, −20.70%, *p* < .05 for 60 min post lunch) and aspartame condition (−12.00 IU/kg, −19.40%, *p* < .05 for 30 min post lunch; −8.00 IU/kg, −14.80%, *p* < .05 for 60 min post lunch) as well as at 20 min post preload (−20.00 IU/kg, −41.70%, *p* < .05) respect to sucrose condition (Anton et al., 2010).

In a 2‐month trial were enrolled 20 T2DM patients of which 10 received *Stevia* leaf powder 1 g/day whereas the others were used as controls. At the end of treatment, the supplement significantly decreased FPG (−33.06 mg/dl, −21.10%, *p* < .05) and PPG (−24.57 mg/dl, −10.90%, *p* < .05) respect to baseline values. However, these data should be considered with prudence, since the various constituents of *Stevia* leaf powder might influence the results (Ritu & Nandini, 2016).

Another study investigated the effect of *Stevia* on PPG in 30 volunteers who ingested one of three different preloads containing (a) water, (b) sugar 60 g, and (c) *Stevia* 1 g on three different days followed by an ad libitum pizza meal. It has been observed that sugar preload increased AUC for glucose by comparison to water (*p* = .001) and *Stevia* (*p* = .007), whereas no significant difference between water and *Stevia* preloads was found (*p* = .2). In addition, PPG levels were not significantly different between the three preloads (Farhat et al., 2019).

In the Ajami et al. (2020) conducted on 34 T2DM patients who daily drank black tea sweetened with *Stevia* extract 2% or sucralose tablet at three meals for 8 weeks it has been shown that *Stevia* did not significantly change FPG, 2 hr‐PPG, FPI, and HbA_1c_ levels compared to sucralose (Ajami et al., 2020).

One study showed the glucose‐lowering activity of *S. rebaudiana* in association with other nutraceuticals. Mayasari et al. (2018) explored the efficacy of a combined herbal drink composed of *Hibiscuss sabdariffa* (rosella tea) with *S. rebaudiana* on glucose metabolism in 24 prediabetic women of which 12 received 10 g/day rosella powder with 250 mg/day of *Stevia* sweetener for 14 days while the others were used as controls. In the treatment group, a significant decrease in FPG levels (−22.67 mg/dl, −20.40%, *p* < .01) was found from baseline without affecting those of PPG at 2 hr (Mayasari et al., 2018).

### 3.9 Gymnemic acid [*G. sylvestre* (L) Pers]

Gymnemic acid was isolated from the leaves of *G. sylvestre* (GS), a medicinal plant that belongs to the Asclepiadaceae family. It consists of a mixture of saponins and has shown hypoglycemic activity due to its ability to delay the uptake of glucose into the blood (Pothuraju, Sharma, Chagalamarri, Jangra, & Kumar Kavadi, 2014).

#### 3.9.1 Mechanisms of action

**FIGURE 9:**
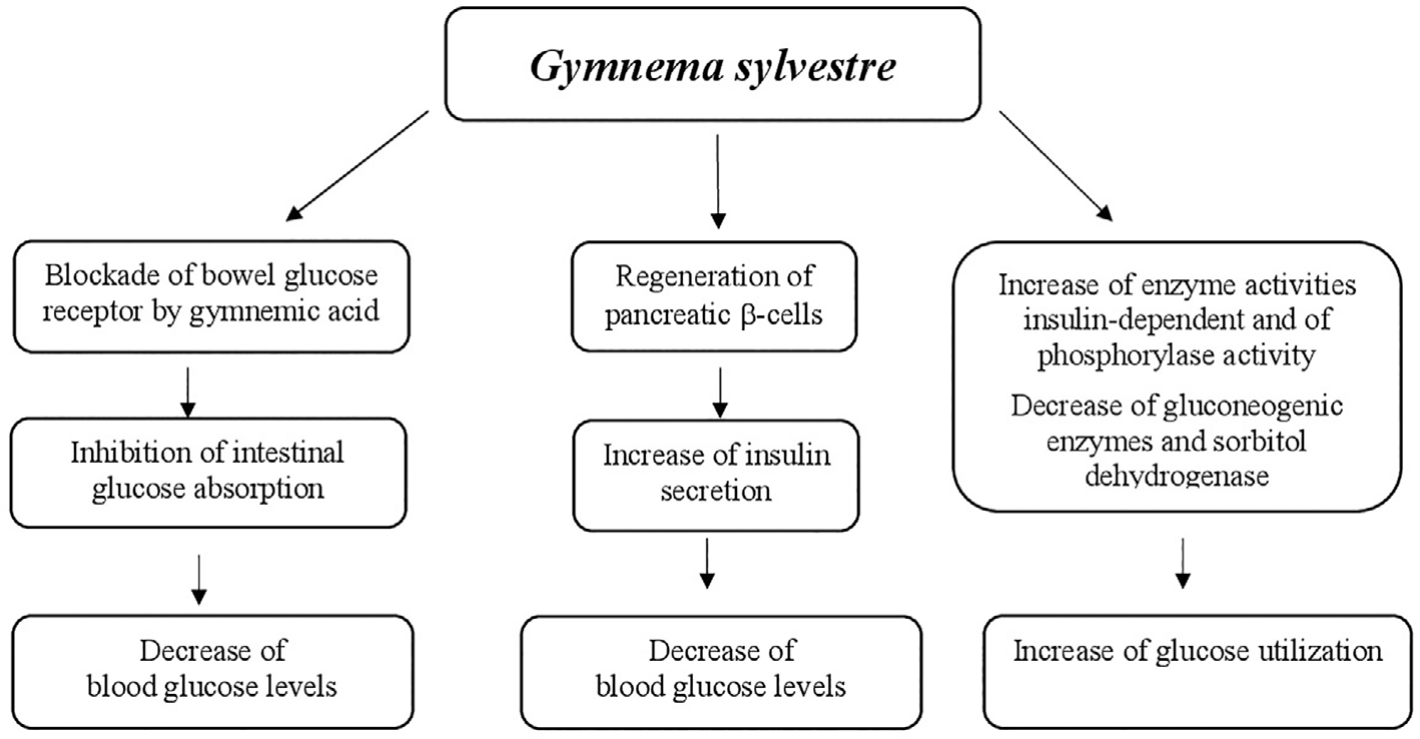
Potential mechanisms of *Gymnema sylvestre* hypoglycemic action

Given the similarity to glucose, gymnemic acid binds the receptor located on the taste buds that, thus, are not activated by the sugar present in the food resulting in non‐absorption of the latter. Gymnemic acid also binds the Na^+^‐glucose symporters located in the external layer of the intestine thus inhibiting glucose absorption (Sahu, Mahato, Sarkar, & Poddar, 1996). Moreover, hypoglycemic effects of gymnemic acid are exerted by increasing insulin secretion and promoting the regeneration of pancreatic β‐cells (Aralelimath & Bhise, 2012; Nakamura, Tsumura, Tonogai, & Shibata, 1999; Shanmugasundaram, Gopinath, Radha Shanmugasundaram, & Rajendran, 1990) (Figure 9). Hypoglycemic effects of gymnemic acid also includes a rise of glucose utilization due to an increase of enzyme activities insulin‐dependent (hexokinase, glycogen synthetase, glyceraldehydes 3‐phosphate dehydrogenase, and glucose 6‐phosphate dehydrogenase) and of phosphorylase activity together with a decrease of gluconeogenic enzymes and sorbitol dehydrogenase (Khan et al., 2019). Moreover, the hypoglycemic action of gymnemic acid is linked to its ability to modulate the incretin effect which results in the stimulation of insulin secretion and its release (Tiwari, Mishra, & Sangwan, 2014).

#### 3.9.2 Clinical trials

Several clinical trials showed the hypoglycemic activity of different GS extracts (Table 9).

**TABLE 9:** Scheme of clinical studies on *Gymnema sylvestre* (GS)

Subjects	Drug treatment and period	Results	References
Diabetic patients (*n* = 6)	6 g/day decoction of powdered GS leaves shade‐dried 15 days	FPG decrease, PPG at 30 min and 2 hr after OGTT decrease	Mayasari et al. (2018)
T2DM patients (*n* = 22)	400 mg/day GS_4_ 18–20 months	FPG and HbA_1c_ decrease, insulin increase in fasting and post‐prandial state	Baskaran, Kizar Ahamath, Radha Shanmugasundaram, and Shanmugasundaram (1990)
Type 1 diabetic patients (*n* = 27)	400 mg/day GS_4_ 6–30 months	FPG and HbA_1c_ decrease, less insulin requirement, fasting C‐peptide increase	Shanmugasundaram, Rajeswari, et al. (1990)
T2DM patients (*n* = 11)	1 g/day OSA 60 days	FPG and PPG decrease, insulin and C‐peptide increase	Al‐Romaiyan et al. (2010)
T2DM patients (*n* = 58)	250 mg/day capsule of GS leaf extract 3 months	FPG, PPG and HbA_1c_ decrease, insulin resistance reduction	S. N. Kumar, Mani, and Mani (2010)
T2DM patients (*n* = 32)	1 g/day capsule of GS leaf extract 30 days	FPG decrease	Li et al. (2015)
IGT patients (*n* = 30)	600 mg/day capsule of GS leaf extract 12 weeks	HbA_1c_ decrease, PPG 2 hr after OGTT decrease	Gaytán Martínez, Sánchez‐Ruiz, Zuñiga, González‐Ortiz, and Martínez‐Abundis (2021)

Abbreviations: FPG, fasting plasma glucose; GS_4_, ethanol extract of GS leaves; HbA_1c_, glycated hemoglobin; IGT, impaired glucose tolerance; OGTT, oral glucose tolerance test; OSA, Om Santal Adivasi; PPG, post‐prandial plasma glucose; T2DM, type 2 diabetes mellitus.

One study investigated the effect of a decoction of powdered GS leaves shade‐dried (10 g/100 ml) intake at the dose of 2 g three times daily for 15 days in 6 diabetic patients that were not taking any therapy. They were subjected to an OGTT before and after the intervention. It has been observed that the supplement produced a significant reduction of FPG values (−25.00 mg/dl, −18.40%, *p* < .02) as well as in PPG levels at 30 min (−39.30 mg/dl, −17.90%, *p* < .05) and 2 hr (−31.60 mg/dl, −20.70%, *p* < .01) after OGTT compared to control (Khare, Tondon, & Tewari, 1983).

Afterward, it has been shown that in a cohort of 22 T2DM patients treated with conventional oral hypoglycemic therapy the assumption of capsules containing an ethanol extract of GS leaves (GS_4_) 400 mg/day for 18–20 months resulted in a reduction of FPG levels (−50.00 mg/dl, −40.30%, *p* < .001) and HbA_1c_ values (−3.43%, −35.50%, *p* < .001) respect to baseline as well as an increase of insulin levels in the fasting (+8.00 mg/dl, +61.50%, *p* < .01) and post‐prandial state (+13.00 mg/dl, +26.00%, *p* < .01) compared to diabetics without supplement (Baskaran et al., 1990).

The supplement used in the study of Baskaran et al. (1990) was also given to 27 type 1 diabetes insulin‐dependent patients for a period ranging from 6 to 30 months. At the end of treatment, it has been observed a not significant decrease in FPG values (−80.00 mg/dl, −34.50%) and a reduced insulin daily requirement of about 50%, compared to baseline, probably because of supplement capacity to promote the remaining pancreatic β‐cells regeneration. Moreover, it has been shown that GS leaf extract determined a significant lowering of HbA_1c_ levels (−3.30%, −31.00%, *p* < .001) with respect to baseline during the initial 6–8 months of treatment, and this decrease was observed until the end of treatment. However, HbA_1c_ values were higher than those of controls. In addition, the supplement administration produced a significant increase in fasting C‐peptide levels (+0.08 mg/dl, +76.20%, *p* < .001) compared to diabetics without supplements which is indicative of improvement in the functionality of pancreatic β‐cells (Shanmugasundaram, Rajeswari, et al., 1990).

In a cohort of 11 patients with T2DM treated with a novel high molecular weight of GS leaf extract (Om Santal Adivasi [OSA]) 1 g/day for a period of 60 days it has been observed a significant reduction in FPG (−43.00 mg/dl, −26.50%, *p* < .005) and PPG levels (−55.00 mg/dl, −18.90%, *p* < .02) respect to baseline. It has been also reported an increase in insulin (+8.00 μU/ml, +33.30%, *p* < .001) and C‐peptide levels (+149.00 pmol/L, +50.00%, *p* < .05) compared to baseline (Al‐Romaiyan et al., 2010). Moreover, it has been demonstrated that OSA enhances insulin secretion in the isolated human islet of Langerhans (Al‐Romaiyan et al., 2012; B. Liu et al., 2009).

Another study showed that in 58 T2DM patients the intake of capsule enclosing GS leaf extract (250 mg) for 3 months determined a significant decrease of FPG (−26.20 mg/dl, −13.90%, *p* < .005) and PPG levels (−59.30 mg/dl, −21.50%, *p* < .001) with a concomitant reduction of HbA_1c_ value (−1.00%, −13.60%, *p* < .001) respect to baseline. After GS supplementation, it was also found a statistically significant lowering of glucose/insulin ratio (−10.00, −33.60%, *p* = .01) compared to baseline, together with a reduction of HOMA IR values (−0.87, −15.30%, *p* = .36) and an increase of HOMA B (+0.51, +20.20%, *p* = .26), were indicative of an insulin resistance reduction (S. N. Kumar et al., 2010).

Li et al. (2015) observed that the assumption of powdered GS leaves in hard gelatin capsules, 1 g/day, for 30 days in a group of 32 patients with T2DM produced a significant decrease in FPG levels (−81.00 mg/dl, −37.00%, *p* < .05) respect to baseline (Li et al., 2015).

Recently has been studied, for the first time, the effect of GS leaf extract administration in IGT patients not taking hypoglycemic agents. A total of 30 subjects were enrolled and divided in two groups of 15 individuals each that received either an oral capsule containing 300 mg of GS leaf powder, or a placebo, twice daily for 12 weeks. These patients were also subjected to an OGTT before and after the supplement intake. At the end of the study, it has been observed that the GS leaf extract assumption resulted in a significant decrease in HbA_1c_ values (−0.40%, −10.00%, *p* = .003) and PPG levels at 2 hr (−23.40 mg/dl, −14.30%, *p* = .025) after OGTT respect to baseline, thus entailing an increase of insulin sensitivity (Gaytán Martínez et al., 2021).

### 3.10 Ginseng [*Panax ginseng* (L) Pers and *Panax quinquefolius* (L) Pers]

The Asian (*P. ginseng*) and American (*P. quinquefolius*) ginseng are the main botanical species that showed hypoglycemic action. Ginseng contains different compounds among which saponins, also named ginsenosides, are thought to be involved in the hypoglycemic action of ginseng.

#### 3.10.1 Mechanisms of action

**FIGURE 10:**
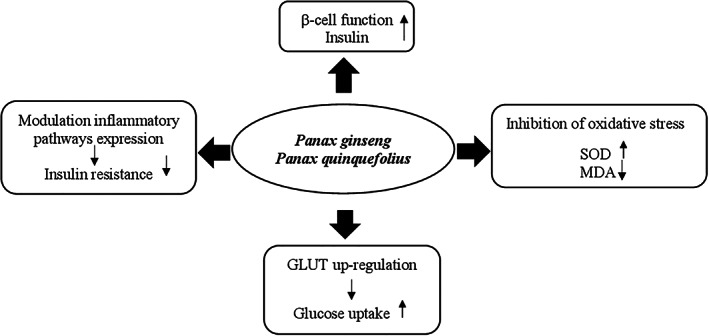
Potential mechanisms of *Panax ginseng* and *Panax quinquefolius* hypoglycemic effect. GLUT, glucose transporter; MDA, malondialdehyde; SOD, superoxide dismutase

The potential mechanisms that underlie the regulation of blood glucose by ginseng may be related to (a) pancreatic β‐cell function improvement and insulin sensitivity enhancement; (b) increase of glucose uptake through up‐regulating the GLUT expression; (c) inhibition of oxidative stress by rising superoxide dismutase (SOD) activity and lowering malondialdehyde (MDA) levels; and (d) modulation of inflammatory pathways expression (e.g., TNF‐α or endothelial nitric oxide synthase [eNOS]) to prevent insulin resistance development (Figure 10). However, the mechanisms of hypoglycemic action by ginseng are not yet fully clarified (W. Chen, Balan, & Popovich, 2019).

#### 3.10.2 Clinical trials

Many human studies have investigated the glucose‐lowering effect of ginseng (Asian ginseng and American ginseng) using different extracts or differently products processed (Table 10).

**TABLE 10:** Summary of clinical trials on hypoglycemic effect of *Panax ginseng* and *Panax quinquefolius*

Subjects	Drug treatment and period	Results	References
Non‐insulin‐dependent diabetic patients (*n* = 36)	100–200 mg/day unspecified ginseng type 8 weeks	FPG and HbA_1c_ decrease	Sotaniemi, Haapakoski, and Rautio (1995)
T2DM patients (*n* = 19)	6 g/day Korean red ginseng 12 weeks	FPI decrease HOMA‐IS index improvement Glucose and insulin AUC decrease	Vuksan et al. (2008)
T2DM patients (*n* = 20)	2.214 g/day *Panax ginseng* roots 4 weeks	FPG decrease HOMA‐IR decrease	Ma et al. (2008)
T2DM patients (*n* = 38)	780 mg/day fermented red ginseng 12 weeks	FPG and HbA_1c_ decrease FPI and HOMA‐IR decrease	H. O. Kim, Park, and Han (2011)
IFG patients (*n* = 45)	20 g/day red ginseng cheonggukjang 8 weeks	FPG decrease	Shin et al. (2011)
T2DM patients (*n* = 72)	1.5, 2, 3 g/day vinegar extract of ginseng 8 weeks	FPG and HbA_1c_ decrease	Yoon et al. (2012)
IFG patients (*n* = 23)	960 mg/day hydrolysed ginseng extract 8 weeks	FPG decrease; PPG at 60 min after OGTT decrease	Park et al. (2014)
IFG, IGT, T2DM patients (*n* = 41)	5 g/day Korean red ginseng 12 weeks	Serum and whole blood glucose at 30 min after OGTT decrease Serum insulin at 0 and 30 min after OGTT decrease C‐peptide at 0 and 30 min after OGTT decrease C‐peptide at 30 min after OGTT decrease insulin and C‐peptide AUC decrease HOMA‐IR decrease	Bang, Kwak, Ahn, Shin, and Lee (2014)
IFG, T2DM patients (*n* = 42)	2.7 g/day fermented red ginseng 4 weeks	FPG decrease PPG 2 hr after meal tolerance test decrease PPI 2 hr after meal tolerance test increase Glucose AUC improvement	M. R. Oh et al. (2014)
T2DM patients (*n* = 25)	Escalating doses (1, 3, 6 g) Korean white ginseng during five visits	Glycemic parameters unaffected	Shishtar, Jovanovski, Jenkins, and Vuksan (2014)
FPG levels 100–140 mg/dl subjects (*n* = 72)	1 g/day *Panax ginseng* berry extract 12 weeks	FPG decrease PPG at 60 min after OGTT decrease Glucose AUC decrease	H. S. Choi et al. (2018)
T2DM patients (*n* = 9)	3 g/day AG during four visits	PPG decrease Glucose AUC decrease	Vuksan, Sievenpiper, et al. (2000)
T2DM patients (*n* = 10)	3, 6, 9 g AG 120, 80, 40, and 0 min before 25‐g GCT	PPG decrease; Glucose AUC decrease	Vuksan, Stavro, et al. (2000)
T2DM patients (*n* = 23)	3 g/day AG 8 weeks	FPG and HbA_1c_ decrease	Vuksan, Xu, et al. (2000)
T2DM patients (*n* = 39)	KGB 6 g/day plus AG 3 g/day 12 weeks	HbA_1c_ decrease	A. L. Jenkins et al. (2018)
T2DM patients (*n* = 24)	3 g/day AG 8 weeks	FPG and HbA_1c_ decrease	Vuksan et al. (2019)
T2DM patients (*n* = 80)	2.25 g/day Korean red ginseng + AG 12 weeks	HbA_1c_ decrease	Jovanovski et al. (2021)
IGT and T2DM patients (*n* = 62)	6 g/day mixture of Korean red ginseng powder, banaba leaf extract, mulberry leaf extract 6 months	Glucose AUC decrease Downward trend insulin AUC	H. J. Kim, Yoon, et al. (2012)

Abbreviations: AG, American ginseng; AUC, area under the curve; FPG, fasting plasma glucose; FPI, fasting plasma insulin; GCT, glucose challenge test; HbA_1c_, glycated hemoglobin; HOMA‐IR, homeostatic model assessment of insulin resistance; HOMA‐IS index, homeostatic model assessment of insulin sensitivity index; IFG, impaired fasting glucose; IGT, impaired glucose tolerance; KGB, konjac‐glucomannan‐based fiber blended; OGTT, oral glucose tolerance test; PPG, post‐prandial plasma glucose; PPI, post‐prandial plasma insulin; T2DM, type 2 diabetes mellitus.

Sotaniemi et al. (1995) reported the effect of ginseng supplementation (Danks Droge, Copenhagen) at the dose of 100 or 200 mg/day for 8 weeks in 36 non‐insulin‐dependent diabetic patients. It has been observed that the dose of 100 mg significantly decreased FPG levels (−10.81 mg/dl, −7.20%, *p* < .05) while that of 200 mg reduced HbA_1c_ values (−6.00 mmol/mol, [−0.50%], −12.50%, *p* < .05) compared to placebo. However, the authors did not specify the type of ginseng source (Sotaniemi et al., 1995).

As regards studies on Asian ginseng, one trial showed that the assumption of Korean red ginseng (KRG) at the dose of 6 g/day for 12 weeks in 19 patients with well‐controlled T2DM resulted in a significant decrease in FPI levels (−2.31 μΙU/ml, −34.10%, *p* < .05) and an improvement of HOMA‐IS index (+0.1, +33.00%, *p* < .05) respect to placebo. In addition, KRG significantly reduced the AUC of plasma glucose by 8% and the AUC of plasma insulin by 34% compared with placebo (Vuksan et al., 2008).

Ma et al. (2008) investigated the action of *P. ginseng* roots administration at the dose of 2.214 g/day for 4 weeks in 20 T2DM subjects treated with conventional oral hypoglycemic agents. It has been observed a significant decrease in FPG levels (−3.60 mg/dl, −2.40%, *p* < .05) by ginseng with respect to the difference in response to placebo (+34.23 mg/dl, +22.90%) and a downward trend for FPI values (−4.32 μΙU/ml, −20.10%) after supplementation which can partly explain the significant HOMA‐IR reduction (−3.30, −44.60%, *p* < .05) after ginseng administration respect to after placebo (−0.90, −12.20%) (Ma et al., 2008).

H. O. Kim et al. (2011) reported that supplementation with 780 mg/day of fermented red ginseng (FRG) for a period of 12 weeks in 38 T2DM patients produced a significant decrease in FPG (−8.58 mg/dl, −6.30%, *p* < .05) and HbA_1c_ values (−0.40%, −7.00%, *p* < .05) compared to baseline. Regarding changes in HbA_1c_ by levels, it has been observed that the supplement induced a higher reduction in high HbA_1c_ (≥8%) (−0.63%, −10.10%, *p* < .05) with respect to low HbA_1c_ (<8%) (−0.27%, −6.00%). FRG administration also determined a significant decrease in FPI levels (−1.85 μIU/ml, −20.20%, *p* < .05) compared to baseline and HOMA‐IR (−0.38, −14.10%, *p* < .01) respect to placebo (H. O. Kim et al., 2011).

One study showed that red ginseng cheonggukjang, an extract of Korean red ginseng with cheonggukjang (fermented soybean), at the dose of 20 g/day for 8 weeks in 45 IFG subjects significantly lowered FPG levels compared to baseline values (−20.60 mg/dl, −17.10%, *p* < .001) and placebo (−18.10 mg/dl, −15.30%, *p* < .05) at the end of treatment (Shin et al., 2011).

Yoon et al. (2012) reported the effect of a vinegar extract of ginseng (ginsam) at three different doses (1.5, 2, and 3 g/day) for 8 weeks in 72 drugs naïve T2DM patients. It has been observed a significant decrease of FPG (−3.07 mg/dl, −2.10%, *p* = .043) and HbA_1c_ levels (−0.34%, −5.10%, *p* = .021) compared to placebo only in the group who received 1.5 g/day of ginsam (Yoon et al., 2012).

Another study demonstrated that the supplementation with 960 mg/day of a hydrolyzed ginseng extract, for 8 weeks in 23 IFG patients determined a significant reduction of FPG (*p* = .017) and PPG concentration at 60 min after OGTT (*p* = .01) respect to placebo (Park et al., 2014).

Bang et al. (2014) reported the effect of KRG (*P. ginseng* C.A. Meyer) supplementation, 5 g/day, in 41 patients who included those with IFG, IGT, and newly diagnosed T2DM. After 12 weeks of treatment both serum glucose (−22.23 mg/dl, −10.60%, *p* = .016) and whole blood glucose levels (−17.53 mg/dl, −8.10%, *p* = .002) were reduced at 30 min after OGTT compared to baseline. Among the study population, IFG and IGT subjects exhibited a significant decrease in serum (*p* = .041) and whole blood glucose values (*p* = .016) at 30 min, while those with T2DM showed a downward trend only in whole blood glucose concentration (*p* = .059) at 30 min after OGTT. It has also been shown that KRG assumption produced in IFG, IGT, and T2DM patients a significant decrease in serum insulin levels at 0 min (−0.90 μIU/dl, −13.40%, *p* = .024) and 30 min (−7.67 μIU/dl, −29.00%, *p* = .007) with a tendency to lowering at 60 min (−7.50 μIU/dl, −13.40%, *p* = .070) and 120 min (−8.24 μIU/dl, −21.00%, *p* = .070) during OGTT respect to baseline. Similarly, it has been observed that C‐peptide concentration was significantly lowered at 0 min (−0.31 μEq/L, −14.80%, *p* = .042) and 30 min (−0.79 μEq/L, −14.50%, *p* = .008) with a trend to a decrease at 60 min (−0.68 μEq/L, −7.80%, *p* = .085) after OGTT compared to baseline. Moreover, after the intervention, C‐peptide values were significantly reduced (*p* = .013) at 30 min during OGTT with respect to placebo. In addition, the supplement significantly lowered insulin (*p* = .006) and C‐peptide AUC (*p* = .039). About glucose‐linked markers, serum insulin levels at 30 min (*p* = .041) and 60 min (*p* = .028), insulin AUC (*p* = .033), and C‐peptide at 30 min (*p* = .050) were significantly decreased in IFG and IGT groups, whereas in T2DM patients were found reduced serum insulin (*p* = .012) and C‐peptide values (*p* = .022) at 120 min, as well as C‐peptide AUC (*p* = .010). There was also a tendency to a decrease in serum insulin levels at 0 min (*p* = .066) and 30 min (*p* = .079), insulin AUC (*p* = .092), and C‐peptide at 30 min (*p* = .059) and 60 min (*p* = .074) after OGTT. At last, a 12 weeks of KRG supplementation in IFG, IGT, and T2DM subjects determined a significant lowering of HOMA‐IR (−0.28, −15.50%, *p* < .05) compared to baseline values (Bang et al., 2014).

In a 4‐week clinical study including 42 IFG or T2DM naïve patients, the assumption of 2.7 g/day of FGR significantly reduced FPG levels (−7.20 mg/dl, −6.20%, *p* = .039) compared to baseline values. As regards post‐prandial parameters assessed during the meal tolerance test, the supplement produced a significant decrease of 2 hr‐PPG (−14.41 mg/dl, −9.40%, *p* = .008) and an increase of 2 hr‐PPI (+20.80 μΙU/ml, +58.60%, *p* = .040) compared to placebo. The FGR assumption also determined a significant improvement in the glucose AUC (−3.80 mmol/L, −22.00%, *p* = .013) compared to placebo (M. R. Oh et al., 2014).

However, unlike the other types of Asian ginseng, the acute assumption of Korean white ginseng, an air‐dried variety, at escalating doses (1, 3, or 6 g) did not affect any glycemic parameters in 25 T2DM patients, during five visits (Shishtar et al., 2014).

*P. ginseng* berry has a greater hypoglycemic action than its root with the same dosage probably due to a difference in ginsenosides composition and content which is much higher than the root (Dey et al., 2003; Y. K. Kim et al., 2009).

The administration of ginseng berry extract, 1 g/day, for 12 weeks in 72 subjects with FPG values between 100 and 140 mg/dl, determined a significant decrease of FPG (−4.44 mg/dl, −3.70%, *p* = .035), and PPG concentration at 60 min after OGTT (−24.81 mg/dl, −10.70%, *p* = .006) as well as the glucose AUC (−7.7%, *p* = .024) compared to a baseline without, however, bringing the blood sugar to normal levels (H. S. Choi et al., 2018).

In literature, there are also human trials focused on the hypoglycemic effect of American ginseng (AG). In some short‐term clinical studies, it has been reported that AG affects PPG. In this regard, the administration of 3 g AG on 4 separate occasions both 40 min before and together with a 25‐g oral glucose challenge test (GCT) in 9 patients with well‐controlled T2DM, determined a significant decrease in PPG levels. In particular, when AG was given 40 min before the glucose challenge, PPG lowering was found at 30 min (−18.02 mg/dl, −20.80%, *p* < .05) and 45 min (−14.42 mg/dl, −15.10%, *p* < .05), while when was consumed together with GCT, the decrease of this parameter was observed at 45 min (−19.82 mg/dl, −20.80%, *p* < .05) and 60 min (−23.43 mg/dl, −26.50%, *p* < .05) respect to placebo. The supplement also produced a significant reduction of blood glucose AUC, both when it was given before (−22.00%, *p* < .05) and together with GCT (−19.00%, *p* < .05) compared to placebo (Vuksan, Sievenpiper, et al., 2000).

Another study reported that AG produced a lowering in PPG levels regardless of dose and time administration in 10 T2DM subjects treated with doses of 3, 6, and 9 g and time assumptions of 120, 80, 40, and 0 min before a 25‐g GCT. Any dose of AG produced a similar PPG levels reduction at 30 min (16.3, 18.4, and 18.4% with 3, 6, and 9 g, respectively), 45 min (12.5, 14.3, and 14.3% with 3, 6, and 9 g respectively) and 120 min (59.1, 40.9, and 45.5% with 3, 6, and 9 g respectively) (*p* < .05 for all) from the beginning of GCT respect to placebo. The three AG doses also lowered blood glucose AUC by 19.7% with 3 g, 15.3% with 6 g and 15.9% with 9 g (*p* < .05 for all) compared to placebo. As regards the time of AG assumption, it did not affect PPG levels and blood glucose AUC. These data suggest that 3 g of AG may be the required dose to achieve desirable PPG lowering independently by the time of administration (Vuksan, Stavro, et al., 2000).

One long‐term clinical trial evaluated the effect of an AG extract at the dose of 3 g/day for 8 weeks in 23 T2DM patients. At the end of treatment, the supplement caused a significant decrease of FPG (−21.62 mg/dl, −12.80%, *p* = .006) and HbA_1c_ levels (−0.10%, −1.90%, *p* < .02) respect to baseline values. Furthermore, FPG and HbA_1c_ showed an end difference of 9.4 ± 3.2% (*p* = .008) and 4.2 ± 1.4% (*p* = .007) between AG and placebo, respectively. It has been also observed a not significant increase of 15.9 ± 10.9% (*p* = .12) in FPI levels suggests a potential ameliorating of β‐cell function by AG (Vuksan, Xu, et al., 2000).

A. L. Jenkins et al. (2018) showed that the co‐administration of konjac‐glucomannan‐based fiber blended (KGB) 6 g/day and AG 3 g/day for 12 weeks in 39 T2DM taking conventional therapy produced a significant decrease in HbA_1c_ levels (−0.31%, −6.00%, *p* = .011) compared to control (A. L. Jenkins et al., 2018).

It has been also demonstrated that 8‐week supplementation with 3 g/day of AG extract in 24 T2DM subjects controlled by conventional therapy resulted in a significant decrease of FPG (−9.55 mg/dl, −6.10%, *p* = .008) and HbA_1c_ levels (−0.27%, −5.40%, *p* = .041) compared to placebo (Vuksan et al., 2019).

One study also evaluated the hypoglycemic effect of the Asian and American ginseng association. It has been observed that the consumption of KRG and AG combination at the dose of 2.25 g/day for 12 weeks in 80 T2DM patients who maintained conventional therapy determined a significant lowering in HbA_1c_ values (−0.54%, −10.90%, *p* = .002) respect to control (Jovanovski et al., 2021).

Moreover, it has been investigated the effect of Korean red ginseng powder, banaba leaf extract, and mulberry leaf extract associated with glycemic parameters as previously described (H. J. Kim, Yoon, et al., 2012).

### 3.11 Naringenin [*Citrus* (L) Pers]

Naringenin is a citrus fruit‐derived flavonoid largely present in orange, grapefruit, tangerine, raw lemon peels, and raw lime peels. It is the aglycone form of naringin, but it resulted to be biologically more potent despite both flavonoid compounds showing antioxidant, antiinflammatory, cardioprotective, and hepatoprotective effects. Naringenin attracted interest to the scientific community because of its wide variety of pharmacological activities and its abundance in the diet. Moreover, this flavonoid presented a promising hypoglycemic effect (Joshi, Kulkarni, & Wairkar, 2018; Nyane, Tlaila, Malefane, Ndwandwe, & Owira, 2017).

#### 3.11.1 Mechanisms of action

**FIGURE 11:**
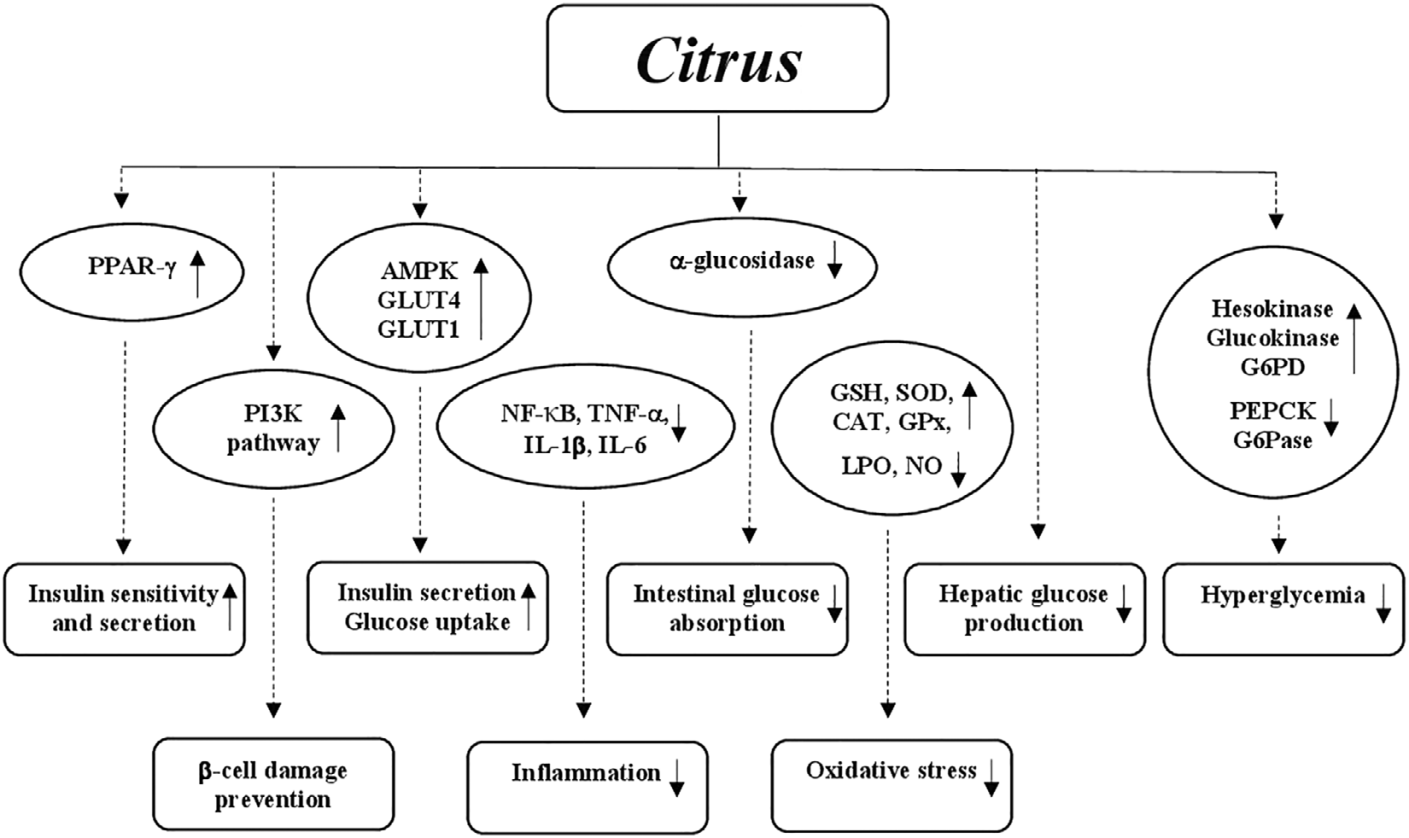
Hypoglycemic effects of *Citrus*. AMPK, 5′ adenosine monophosphate‐activated protein kinase; CAT, catalase; G6Pase, glucose 6‐phosphatase; G6PD, glucose‐6‐phosphate dehydrogenase; GLUT, glucose transporter; GPx, glutathione peroxidase; GSH, reduced glutathione; IL, interleukin; LPO, lipid peroxidation; NF‐κB, nuclear factor‐kappaB; NO, nitric oxide; PEPCK, phosphoenolpyruvate carboxykinase; PI3K, phosphatidylinositol 3‐kinase; PPAR‐γ, peroxisome proliferator activated receptor gamma; SOD, superoxide dismutase

Different mechanisms of naringenin hypoglycemic activity were reported comprising its ability to (a) improve insulin sensitivity and secretion, (b) enhance peripheral glucose uptake, (c) reduce intestinal glucose absorption, (d) suppress hepatic glucose production, (e) regulate the expression of enzymes involved in glycolysis and gluconeogenesis, (f) prevent pancreatic β‐cell damage, and (g) attenuate markers of inflammation and oxidative stress (Joshi et al., 2018) (Figure 11).

#### 3.11.2 Clinical trials

Human studies on naringenin hypoglycemic effect are limited (Table 11).

**TABLE 11:** Summary of human studies on hypoglycemic effect of *Citrus*

Subjects	Drug treatment and period	Results	References
Obesity patients (*n* = 91)	Placebo capsule with 207 ml of apple juice, grapefruit capsules contained 500 mg of whole grown grapefruit freeze‐dried and compressed with 207 ml of apple juice, 237 ml of grapefruit juice with placebo capsule, or half of a fresh grapefruit with placebo capsule, thrice daily 12 weeks	2 hr‐PPI decrease	Fujioka, Greenway, Sheard, and Ying (2006)
Overweight patients (*n* = 95)	900 mg/day citrus polyphenol extract (at least 20% naringin) 12 weeks	FPG and HbA_1c_, decrease	Dallas et al. (2014)
T2DM patients (*n* = 1)	450 mg/day naringenin 8 weeks	Insulin and HOMA‐IR decrease	Murugesan et al. (2020)

Abbreviations: FPG, fasting plasma glucose; HbA_1c_, glycated hemoglobin; HOMA‐IR, homeostatic model assessment of insulin resistance; PPI, post‐prandial insulin; T2DM, type 2 diabetes mellitus.

Grapefruit and its products are the main sources of naringenin and naringin. A 12‐week trial explored the effect of fresh grapefruit, grapefruit juice, grapefruit capsules, and/or placebo on insulin resistance in 91 obese subjects, 34% of whom had metabolic syndrome. The patients received either placebo capsule with 207 ml of apple juice, grapefruit capsules containing 500 mg of whole grown grapefruit freeze‐dried and compressed with 207 ml of apple juice, 237 ml of grapefruit juice with a placebo capsule, or half of a fresh grapefruit with a placebo capsule thrice daily before each meal. At the end of treatment, glucose tolerance testing showed a significant reduction in 2 hr‐PPI only in the fresh grapefruit group (−35 μU/ml, *p* = .046) compared to the grapefruit capsules group. Moreover, considering subjects with metabolic syndrome 2 hr‐PPI decreased more in the grapefruit juice group (−59 μU/ml, *p* < .05) than in the grapefruit capsule and placebo one as well as in the fresh grapefruit group (−76 μU/ml, *p* < .04) compared to placebo one (Fujioka et al., 2006).

Another trial was conducted on 95 healthy overweight subjects given 900 mg/day of a citrus polyphenol extract containing naringin or placebo at breakfast and lunch for 12 weeks. It has been shown that the supplement significantly reduced FPG levels both to baseline (−10.81 mg/dl, −10.30%, *p* < .05) and to placebo (−12.61 mg/dl, −11.90%, *p* < .0001) as well as HbA_1c_ values (−0.84%, −12.40%, *p* < .0001) respect to placebo (Dallas et al., 2014).

Recently, a case study reported that the daily administration of 1,608 mg extract containing 450 mg naringenin for 8 weeks in women with T2DM reduced insulin levels (−2.30 μU/ml, −18.00%) and HOMA‐IR values (−0.60, −17.60%). Further, the treatment did not decrease plasma glucose (Murugesan et al., 2020).

Deserve to be mentioned also an epidemiological study in which it has been evaluated the association between the ingestion of flavonoids, including naringenin, and the risk of chronic diseases. Particularly in 10.054 participants, it has been observed that the intake of this supplement at the dose of 5.1 mg/day has a trend to decrease the risk of T2DM (Knekt et al., 2002).

Moreover, another study evaluated blood levels of a variety of metabolites among which naringenin in 42 children of which 15 were normal weight, 13 were overweight and 14 were obese and it was found a negative correlation between blood naringenin levels (273.08 mg/ml) and HOMA‐IR (r = −0.42, *p* = .006) (Farook et al., 2015).

### 3.12 *T. faenum graecum* (L) Pers

*T. faenum graecum* also known as fenugreek is a plant that belongs to the Fabaceae family and is native to India, China, and North Africa. This herb is commonly used in cooking but also in the treatment of various conditions comprising diabetes, hyperlipidemia, arthritis, hypothyroidism, cancer, ophthalmic disorders, oxidative stress, inflammation, hot flashes in menopausal women, dysmenorrhea, hair growth, and infertility (Bahmani, Shirzad, Mirhosseini, Mesripour, & Rafieian‐Kopaei, 2016; Idris, Mishra, & Khushtar, 2021). Among different parts of fenugreek, the seeds are mainly used for their hypoglycemic properties due to the presence of bioactive compounds including trigonelle, diosgenin, 4‐hydroxyisoleucine (4‐OH‐Ile), and soluble dietary fiber (Fuller & Stephens, 2015; Idris et al., 2021). Moreover, it has been reported that whole seed powder had the highest hypoglycemic effect while the leaves had the weakest one (R. D. Sharma et al., 1996).

#### 3.12.1 Mechanisms of action

In vivo and in vitro studies explored the molecular mechanisms underlying the hypoglycemic effect of the three most studied bioactive compounds present in fenugreek represented by diosgenin, 4‐OH‐Ile and soluble fiber. Diosgenin, a major aglycone of saponin, acts on the pancreas promoting the restoration of pancreatic β‐cells and insulin secretion, as well as on adipose tissue enhancing PPAR‐γ expression. This compound acts also on the liver downregulating the enzymes involved in gluconeogenesis (decrease of G6Pase) and glucose export and upregulating glucokinase leading to a decrease in hepatic glucose output. Moreover, diosgenin increases the amount of hepatoprotective and antioxidant enzymes (Fuller & Stephens, 2015).

4‐Hydroxyisoleucine, a branched‐chain amino acid derivative, represents the majority of the total content of free amino acids in fenugreek seeds. This substance exerts its insulinotropic and hypoglycemic action by stimulating of insulin secretion which determined an increase in the translocation of GLUT4 to the plasma membrane and thus a rise in glucose uptake in adipose tissue and skeletal muscle. In these last 4 OH‐Ile enhance glucose uptake by PI3K activation and increase basal phosphorylation of Akt (Ser‐473) (Avalos‐Soriano, De la Cruz‐Cordero, Rosado, & Garcia‐Gasca, 2016; Fuller & Stephens, 2015).

**FIGURE 12:**
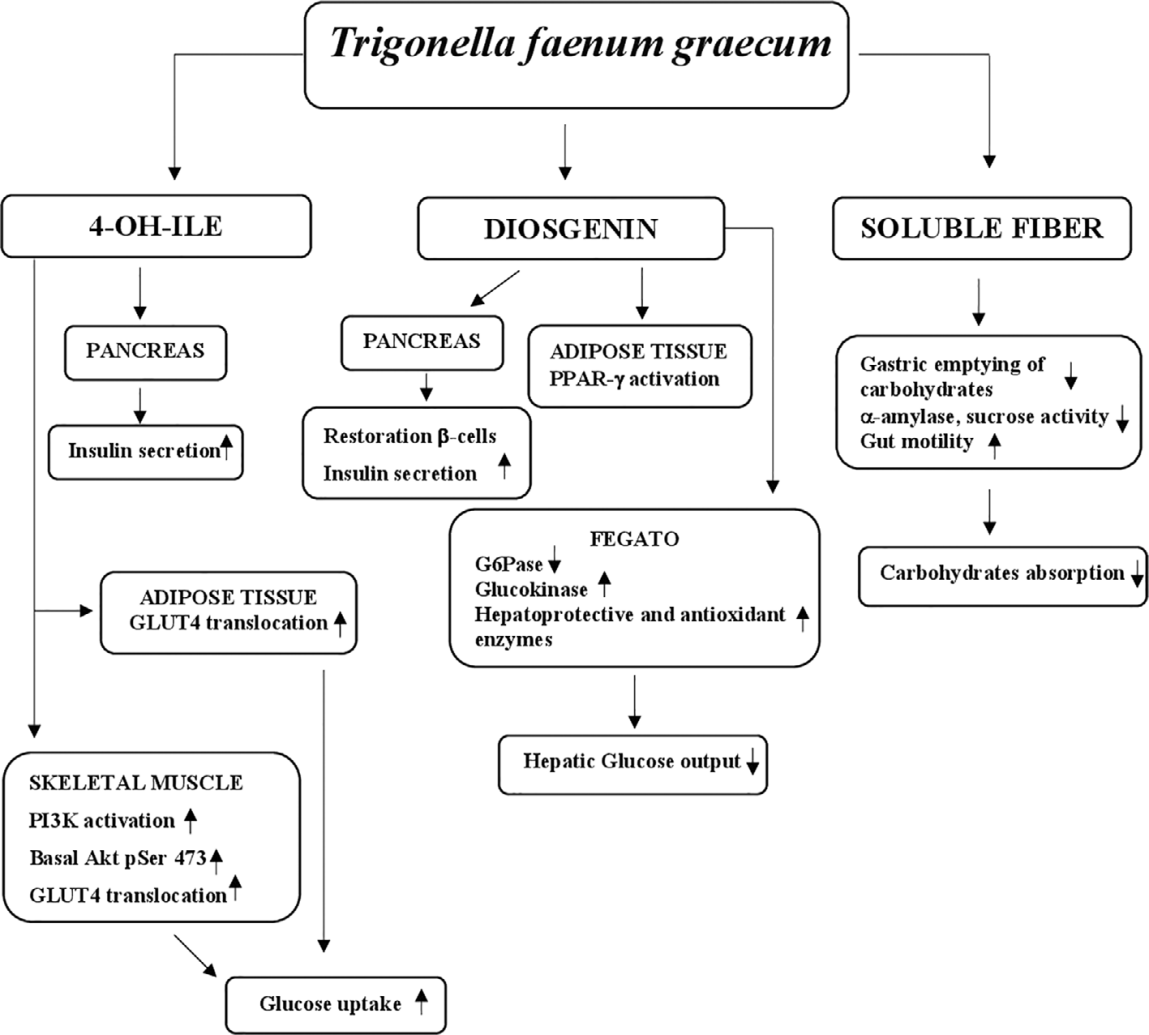
Hypoglycemic effects of *Trigonella faenum graecum* compounds. Phosphorylation at serine 473. 4‐OH‐Ile, 4‐Hydroxyisoleucine; Akt, serine/threonine kinase; G6Pase, glucose‐6‐phosphatase; GLUT, glucose transporter; PI3K, phosphatidylinositol 3‐kinase; PPAR‐γ, peroxisome proliferator‐activated receptor‐γ

Soluble fiber mainly composed by galactomannans exert a hypoglycemic effect by (a) delaying gastric emptying of carbohydrates, (b) reducing α‐amylase and sucrose activity, and (c) increasing gastrointestinal motility (Fuller & Stephens, 2015) (Figure 12).

#### 3.12.2 Clinical trials

Several human studies examined the role of fenugreek on glucose homeostasis (Table 12).

**TABLE 12:** Summary of human studies on glucose‐lowering effect of *Trigonella faenum graecum*

Subjects	Drug treatment and period	Results	References
T2DM patients (*n* = 21)	15 g/day ground fenugreek 4–7 days	PPG at 30, 60, and 120 min decrease after meal	Madar, Abel, Samish, and Arad (1988)
Type 1 diabetes patients (*n* = 10)	100 g/day defatted fenugreek seeds powder 10 days	FPG, PPG at 30, 60, and 90 min after OGTT, glucose AUC decrease	R. D. Sharma et al. (1990)
T2DM patients (*n* = 15) T2DM patients (*n* = 5)	100 g/day defatted fenugreek seeds powder 10 days 2 100 g/day defatted fenugreek seeds powder 20 days	FPG, FPI, PPG at 30, 60, 90, and 120 min after OGTT, PPI at 30, 90, and 120 min after OGTT, glucose and insulin AUC decrease FPG decrease, glucose tolerance improvement	R. D. Sharma and Raghuram (1990)
T2DM patients (*n* = 60)	25 g/day fenugreek seed powder 24 weeks	FPG, PPG at 30, 60, 90, and 120 min after GTT, PPI at 30, 60, and 120 min after GTT, glucose and insulin AUC decrease; HbA_1c_ decrease after 8 weeks of supplementation	R. D. Sharma et al. (1996)
T2DM patients (*n* = 40)	5 g/day fenugreek 1 months	FPG and PPG decrease	Bordia, Verma, and Srivastava (1997)
T2DM patients (*n* = 25)	1 g/day hydroalcoholic fenugreek seeds extract 2 months	FPG, FPI, 2 hr‐PPG, 2 hr‐PPI, glucose and insulin AUC decrease; Insulin sensitivity increase	A. Gupta, Gupta, and Lal (2001)
T2DM patients (*n* = 69)	18 capsules/day *T. faenum graecum* total saponins 12 weeks	FPG, 2 hr‐PPG and HbA_1c_ decrease	Lu et al. (2008)
Obesity patients (*n* = 18)	4 and 8 g/day fenugreek fiber powder 3 test breakfasts	Insulin AUC increase (8 g/day supplement)	Mathern, Raatz, Thomas, and Slavin (2009)
Overweight patients (*n* = 39)	1,176 g/day hydroalcoholic fenugreek seeds extract 6 weeks	Fasting insulin/glucose ratio	Chevassus et al. (2010)
T2DM patients (*n* = 88)	10 g/day fenugreek seeds powder 8 weeks	FPG, HbA_1c_, FPI and HOMA‐IR decrease	Rafraf, Malekiyan, Asghari‐Jafarabadi, and Aliasgarzadeh (2014)
T2DM patients (*n* = 60)	30 g/day fenugreek seeds 8 weeks	HbA_1c_ decrease	Suchitra and Parthasarathy (2015)
IFG or IGT patients (*n* = 140)	10 g/day debitterized, defatted and deodorized fenugreek fiber 3 years	FPG and PPG decrease; FPI and HOMA‐IR increase Cumulative incidence rate of diabetes decrease	Gaddam et al. (2015)
T2DM patients (*n* = 12)	2 g/day fenugreek seeds 5 g/day glibenclamide 12 weeks	FPI increase (supplement) HbA_1c_ and HOMA‐IR decrease (glibenclamide)	Najdi, Hagras, Kamel, and Magadmi (2019)
T2DM patients (*n* = 50)	15 g/day fenugreek seeds 8 weeks	FPG decrease	Hadi et al. (2020)
T2DM patients (*n* = 30)	12.8 g/day of mixture (5.0 g *T. faenum graecum* + 2.0 g psyllium + 600 mg *Allium sativum* + 600 mg *Aloe vera* + 3.6 g *Nigella sativa* + 1.0 g *Silybum marianum*) 40 days	FPG and HbA_1c_ decrease	Zarvandi et al. (2017)

Abbreviations: 2 hr‐PPG, 2 hour‐post‐prandial glucose; 2 hr‐PPI, 2 hour‐post‐prandial insulin; AUC, area under curve; FPG, fasting plasma glucose; FPI, fasting plasma insulin; HbA_1c_, glycated hemoglobin; HOMA‐IR, homeostatic model assessment of insulin resistance; OGTT, oral glucose tolerance test; T2DM, type 2 diabetes mellitus.

One trial investigated the effect of ground fenugreek on PPG and PPI in 21 non‐insulin‐dependent diabetic subjects after the meal tolerance test performed with and without the addition of the supplement 15 g for a period of 4–7 days. It has been observed that fenugreek significantly decreased PPG at 30, 60, and 120 min after the meal (*p* < .05 for all) whereas did not affect PPI except for the measurements at 120 and 180 min that tended to be lower but without a statistical significance (Madar et al., 1988).

R. D. Sharma, Raghuram, Rao (1990) reported that a sample of 10 type 1 diabetic patients given defatted fenugreek seeds powder 100 g/day during lunch and dinner for 10 days exhibited a significant decrease in FPG levels (−75.68 mg/dl, −27.80%, *p* < .01) compared to control the following supplementation. After OGTT, fenugreek intake reduced PPG values at 30 min (−95.50 mg/dl, −25.10%, *p* < .01), 60 min (−77.48 mg/dl, −17.40%, *p* < .05), and 90 min (−84.68 mg/dl, −19.00%, *p* < .01), as well as glucose AUC (−9,027.03 mg/dl × min, −18.70%, *p* < .05) respect to control. However, FPI, PPI, and insulin AUC were not significantly modified by fenugreek ingestion (R. D. Sharma et al., 1990).

The same authors evaluated the efficacy of fenugreek seeds in another study whose design was identical to that described above and in which recruited 15 T2DM patients. The nutraceutical produced a significant decrease in FPG (−42.00 mg/dl, −23.50%, *p* < .05), PPG at 30 min (−68.00 mg/dl, −23.10%, *p* < .01), 60 min (−93.00 mg/dl, −24.60%, *p* < .01), 90 min (−108.00 mg/dl, −25.80%, *p* < .01), and 120 min (−110.00 mg/dl, −27.40%, *p* < .01) after OGTT as well as AUC of glucose (−5,262.00 mg/dl × min, −26.40%, *p* < .01) compared to initial values. It has been also observed a significant reduction in FPI (−4.00 mU/L, −22.90%, *p* < .05), PPI at 30 min (−16.70 mU/L, −35.80%, *p* < .05), 90 min (−12.30 mU/L, −23.10%, *p* < .05), and 120 min (−17.60 mU/L, −30.00%, *p* < .05) after glucose load together with AUC of insulin (−1,142.00 mU/L × min, −30.20%, *p* < .05) respect to baseline following supplementation. Subsequently, the administration of fenugreek seeds at the same dose in five T2DM subjects for 20 days significantly reduced FPG levels and improved glucose tolerance by higher magnitude (R. D. Sharma & Raghuram, 1990).

R. D. Sharma et al. (1996) also explored the hypoglycemic effect of fenugreek seed powder in 60 T2DM patients who received 25 g/day of the nutraceutical at lunch and dinner for 24 weeks. The supplement significantly reduced FPG (−39.64 mg/dl, −26.20%, *p* < .001), PPG at 30 min (−90.10 mg/dl, −42.00%, *p* < .001), 60 min (−48.65 mg/dl, −19.90%, *p* < .001), 90 min (−70.27 mg/dl, −26.70%, *p* < .001), and 120 min (−86.49 mg/dl, −33.60%, *p* < .001) after GTT as well as AUC for glucose (−4,374.77 mg/dl × min, −40.90%, *p* < .001) compared to baseline. The fenugreek consumption did not alter FPI, whereas significantly decreased PPI at 30 min (−8.30 mU/L, −26.60%, *p* < .01), 60 min (−6.90 mU/L, −17.10%, *p* < .01), and 120 min (−12.40 mU/L, −32.50%, *p* < .01) during GTT along with AUC for insulin (−1,106.40 mU/L × min, −38.20%, *p* < .01) respect to initial levels. Moreover, HbA_1c_ lowered (−1.20%, −12.50%, *p* < .001) in a sample of 40 T2DM after 8 weeks of fenugreek ingestion compared to baseline values (R. D. Sharma et al., 1996).

In a 1‐month study including 40 mild and severe T2DM patients given 5 g/day of fenugreek or placebo it has been shown that the supplement significantly decreased FPG (−32.40 mg/dl, −18.60%, *p* < .01) and PPG (−33.40 mg/dl, −13.60%, *p* < .01) only in subjects with mild diabetes respect to baseline values. However, in severe cases, a slight decrease in both glycemic parameters has been found which did not reach statistical significance (Bordia et al., 1997).

In the trial by A. Gupta et al. (2001) were enrolled 25 newly diagnosed T2DM patients who taken hydroalcoholic extract of fenugreek seeds 1 g/day or a placebo for 2 months. At the end of treatment, it has been observed that the supplement caused a significant reduction of FPG (−28.40 mg/dl, −19.20%, *p* < .05), 2 hr‐PPG (−29.50 mg/dl, −14.00%, *p* < .05), and glucose AUC (−4,322.00 mg/dl × min, −15.70%, *p* < .01) compared to placebo. The fenugreek seed extract administration also significantly decreased FPI (−12.90 μIU/L, −58.10%, *p* < .05), 2 hr‐PPI (−22.16 μIU/L, −48.50%, *p* < .05), and insulin AUC (−26.88 μIU/L × min, −47.70%, *p* < .01) whereas increased insulin sensitivity (+55.76%, +97.60%, *p* < .05) respect to placebo (A. Gupta et al., 2001).

Lu et al. (2008) observed the efficacy of *T. faenum graecum* total saponins on glycemic parameters in 69 T2DM subjects not well controlled by oral sulfonylureas. These patients received 6 capsules of nutraceutical three times daily (0.35 g/capsule) after meals or a placebo for 12 weeks in association with sulfonylureas. After supplementation the levels of FPG, 2 hr‐PPG, and HbA_1c_ resulted significantly decreased both to baseline (−33.15 mg/dl, −21.30%, *p* < .05 for FPG; −69.91 mg/dl, −29.10%, *p* < .01 for 2 hr‐PPG; −1.46%, −18.20%, *p* < .05 for HbA_1c_) and to placebo (−28.65 mg/dl, −19.00%, *p* < .05 for FPG; −45.95 mg/dl, −21.20%, *p* < .01 for 2 hr‐PPG; −1.64%, −20.00%, *p* < .05 for HbA_1c_) (Lu et al., 2008).

A study conducted by Mathern et al. (2009) randomized 18 obese patients to three single meal treatments composed of a test breakfast with fenugreek fiber powder 4 and 8 g or without supplement used as control. The three test breakfasts were ingested on a different mornings at least 48 hr apart. It has been shown that the highest dose of fenugreek fiber powder produced a significant increase in insulin AUC (+12 mIU/L × hr, +8.5%, *p* = .04) with respect to control whereas no differences were reported for glucose AUC among treatments (Mathern et al., 2009).

Another trial showed that the administration of hydroalcoholic fenugreek seed extract 1.176 g/day or placebo in 39 overweight subjects for 6 weeks produced a significant decrease in fasting insulin/glucose ratio (−0.17 mUI/mmol, −16.00%, *p* = .044) without modifying fasting and postprandial glucose and insulin after supplementation (Chevassus et al., 2010).

Rafraf et al. (2014) reported that a sample of 88 T2DM patients given fenugreek seeds powder 10 g/day or 5 g/day of wheat starch for 8 weeks presented a significant decrease of FPG and HbA_1c_ following supplementation with respect to baseline (−43.00 mg/dl, −24.00%, *p* = .0001 for FPG; −1.43%, −15.60%, *p* = .0001 for HbA_1c_) and to control group (−22.00 mg/dl, −13.90%, *p* = .007 for FPG; −0.46%, −5.60%, *p* = .0001 for HbA_1c_). Fenugreek also significantly reduced FPI (−53.97 μU/ml, −78.60%, *p* = .03) and HOMA‐IR (−26.56, −84.60%, *p* = .004) compared to baseline values (Rafraf et al., 2014).

In an 8‐week study comprising 60 uncontrolled T2DM patients who consumed 30 g/day of fenugreek seeds or no intervention it has been shown that the supplement significantly decreased HbA_1c_ (−0.20%, −2.50%, *p* < .05) respect to baseline values (Suchitra & Parthasarathy, 2015).

Gaddam et al. (2015) explored the possible ability of debittered, defatted, and deodorized fenugreek fiber in the form of powder to prevent T2DM in 140 IFG or IGT subjects treated with 10 g/day of supplement or no intervention for a period of 3 years. It has been observed that fenugreek powder determined a significant reduction of FPG (−31.00 mg/dl, −23.70%, *p* < .05) and PPG (−13.90 mg/dl, −9.70%, *p* < .01) whereas FPI (−1.80 mU/L, −17.60%, *p* < .01) and HOMA‐IR (−0.40, −15.40%, *p* < .05) resulted increased significantly compared to baseline. In addition, the supplement decreased the cumulative incidence rate of diabetes with respect to the control group (*p* < .01) (Gaddam et al., 2015).

In the study by Najdi et al. (2019), 12 patients were recruited with T2DM not well controlled by metformin who received fenugreek seeds 2 g/day or glibenclamide 5 mg/day for 12 weeks. The supplement determined a significant increase of FPI (−2.64 μU/ml, −37.80%, *p* = .04) compared to baseline, while did not alter FPG, HbA_1c_, and HOMA‐IR. However, it has been also observed that glibenclamide significantly decreases HbA_1c_ (−1.49%, −17.40%, *p* = .036) and HOMA‐IR (−1.30, −36.80%, *p* = .007) by comparison with fenugreek (Najdi et al., 2019).

One trial performed on 50 T2DM patients treated with fenugreek seeds 15 g/day or nil intervention for 8 weeks showed a significant decrease of FPG levels (−10.01 mg/dl, −7.00%, *p* = .019) after supplementation with respect to control (Hadi et al., 2020).

The hypoglycemic action of *T. faenum graecum* in combination with other nutraceuticals was also studied. It has been reported the efficacy of *T. faenum graecum* is associated with *P. psyllium*, *A. sativum*, *A. vera*, *N. sativa*, and *S. marianum* on glucose metabolism as previously discussed (Zarvandi et al., 2017).

### 3.13 *Syzygium cumini* [*Eugenia jambolana* (L) Pers]

*S. cumini* (*E. jambolana*) is one of the best‐known species of the genus Syzygium belonging to the Myrtaceae family. This plant can be found in India, the Philippines, Thailand, Madagascar, Africa, tropical America, and Carribean. Different parts of *S. cumini* that include leaves, dried seeds, fruits, and bark are used in traditional medicine for the treatment of dysentery, inflammation, and diabetes (Ayyanar & Subash‐Babu, 2012; Zulcafli, Lim, Ling, Chye, & Koh, 2020). In particular, the hypoglycemic effect of this nutraceutical is attributed to the presence of bioactive compounds represented by flavonoids, glycosides, alkaloids, terpenoids, steroids, tannins, phenols, and cardiac glycosides (Gowri & Vasantha, 2010).

#### 3.13.1 Mechanisms of action

**FIGURE 13:**
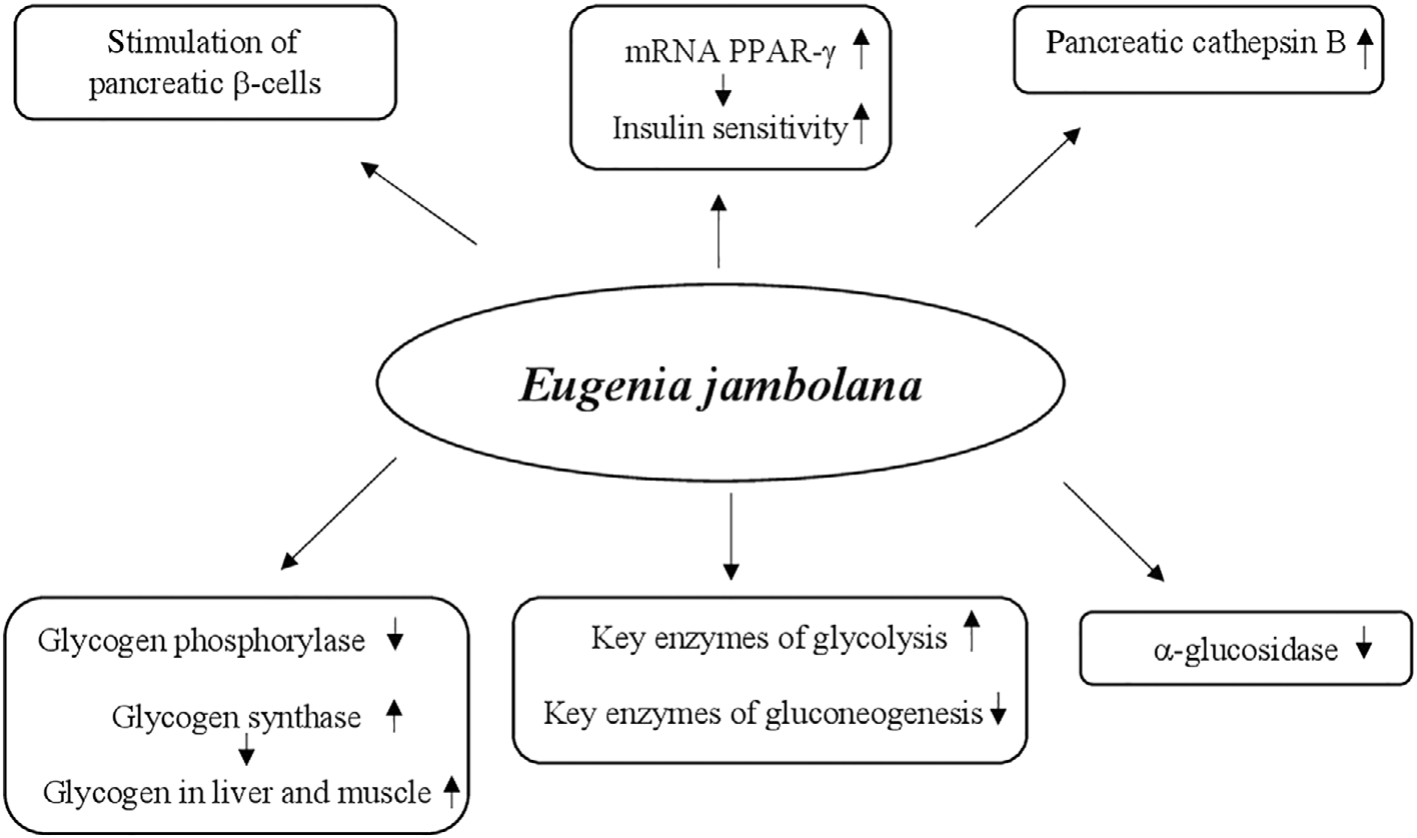
Proposed mechanisms of glucose‐lowering action of *Eugenia jambolana*. PPAR‐γ, peroxisome proliferator‐activated receptor‐γ

Studies in experimental animals reported the hypoglycemic activity of *S. cumini* and the underlying mechanisms of action that appeared to be both pancreatic and extra‐pancreatic. This plant might be exerting its glucose‐lowering effect through (a) the stimulation of surviving pancreatic β‐cells to increase the release of insulin similarly to the hypoglycemic oral agents' sulfonylureas and biguanides; (b) the increase of PPAR‐γ mRNA expression to improve insulin sensitivity; (c) the rise in pancreatic cathepsin B activity to enhance insulin content; (d) the increase in glycogen content of liver and skeletal muscle due to the reduction in the activity of glycogen phosphorylase and the increase in that of glycogen synthase; (e) the rise in the activity of glycolysis key enzymes and the decrease in those of gluconeogenesis; and (f) the inhibition of α‐glucosidase activity to delay the digestion of complex carbohydrate and intestinal absorption of glucose (Ayyanar, Subash‐Babu, & Ignacimuthu, 2013; Zulcafli et al., 2020) (Figure 13).

#### 3.13.2 Clinical trials

*S. cumini* was widely studied starting from the end of the 19th century after the importation from Indies to Europe. In the beginning, especially before the discovery of insulin, clinical case reports were mainly carried out. In these studies, the supplement was administered in the form of seed powder or fluid extracts (fruit or bark) usually at high dosages and some patients took up to 100 g/day of seed powder. In the reports, volume and glucose content of urine were criteria for treatment success although these endpoints can only provide an approximate evaluation of the drug's clinical efficacy. Moreover, the clinical case reports showed conflicting results on the hypoglycemic action of *S. cumini*. After the Second World War a restricted number of clinical studies were performed (Table 13) (Helmstädter, 2008). In the study of Bose and Sepaha (1956), 7 T2DM non‐insulin‐dependent patients received dried pericarp‐free seeds of *S. cumini* and only 3 of them presented a definite drop in urinary and blood glucose levels. However, this study does not satisfy modern criteria for clinical trials (Bose & Sepaha, 1956).

**TABLE 13:** Summary of clinical trials on hypoglycemic effect of *Eugenia jambolana*

Subjects	Drug treatment and period	Results	References
T2DM non‐insulin‐dependent patients (*n* = 7)	*Syzygium cumini* dried pericarp‐free seeds	Urinary and blood glucose drop	Bose and Sepaha (1956 **)**
Healthy subjects (*n* = 7) T2DM patients (*n* = 5)	110 g *S. cumini* fruit pulp without seeds 3 hr	Blood glucose decrease (healthy) Blood glucose not significant increase (T2DM)	Nande, Kale, Wagh, Anatakar, and Vaidya (1981)
T2DM patients (*n* = 28)	4–24 g *S. cumini* seed powder 1 day	FPG and PPG decrease	Srivastava, Venkatakrishna‐Bhatt, Gupta, and Gupta (1983)
T2DM non‐insulin‐dependent patients (*n* = 30)	12 g/day *S. cumini* seed powder 3 months 250 mg/day chlorpropamide 1 month	FPG, 1 hr‐ and 2 hr‐PPG decrease	Kohli and Singh (1993)
Volunteers nondiabetic (*n* = 30)	2 g *S. cumini* dry leaves 2/3 hr	Blood glucose has no effect	Teixeira et al. (1990, 2000)
T2DM patients	2 g/day *S. cumini* dry leaves 10 g/day glyburide 28 days	Blood glucose has no effect	Teixeira et al. (2004)
T2DM patients (*n* = 27)	2 g/day *S. cumini* dry leaves 10 mg/day glyburide 28 days	Blood glucose has no effect	Teixeira, Fuchs, Weinert, and Esteves (2006)
T2DM patients (*n* = 15)	10 g/day *S. cumini* seed powder 6 months	FPG and HOMA IR decrease	Sahana et al. (2010)
T2DM patients (*n* = 99)	10 g/day *S. cumini* seed powder 90 days	FPG, PPG and HbA_1c_ decrease	Sidana et al. (2017)

Abbreviations: FPG, fasting plasma glucose; HbA_1c_, glycated hemoglobin; HOMA‐IR, homeostatic model assessment of insulin resistance; PPG, post‐prandial glucose; T2DM, type 2 diabetes mellitus.

Another trial reported that the administration of about 110 g *S. cumini* fruit pulp without seeds in seven healthy and five T2DM subjects determined, after 3 hr, a significant decrease in blood glucose in the former and a not significant increase in diabetics. This result suggests that the fruit pulp of the supplement is not suitable for the treatment of diabetes (Nande et al., 1981).

Srivastava et al. (1983) evaluated the hypoglycemic effect of *S. cumini* seed powder in 28 T2DM patients. These subjects received 4–24 g of supplement in a gelatin capsule formulation and exhibited a significant decrease in mean FPG (−36.10 mg/dl, −17.30%, *p* < .05) and PPG (−116.60 mg/dl, −31.90%, *p* < .05) levels (Srivastava et al., 1983).

Similar results were obtained from a 3‐month trial conducted on 30 subjects with mild T2DM non‐insulin‐dependent treated with 12 g/day of *S. cumini* seed powder and subjected to OGTT. The supplement significantly decreased FPG after 1 month (−33.40 mg/dl, −20.50%, *p* < .001) and to a greater extent after 2 months of therapy (−63.36 mg/dl, −38.90%, *p* < .001) from baseline. *S. cumini* also resulted in a higher reduction in 1 hr‐ and 2 hr‐PPG levels after 2 months of treatment (−95.45 mg/dl, −34.20%, *p* < .001 for 1 hr‐PPG; −112.05 mg/dl, −36.80%, *p* < .001 for 2 hr‐PPG) than after 1 month (−56.32 mg/dl, −20.20%, *p* < .001 for 1 hr‐PPG; −55.67 mg/dl, −18.30%, *p* < .001 for 2 hr‐PPG) and 3 months (−58.67 mg/dl, −21.00%, *p* < .01 for 1 hr‐PPG; −78.67 mg/dl, −25.80%, *p* < .01 for 2 hr‐PPG) by comparison to baseline values. Moreover, 6 T2DM non‐insulin‐dependent patients were given 250 mg/day of chlorpropamide for 1 month. At the end of treatment, it has been observed a significant decrease of 2 hr‐PPG (−50.50 mg/dl, −18.00%, *p* < .05) with respect to baseline while the lowering in FPG and 1 hr‐PPG did not reach statistical significance mainly due to the little number of patients. Based on these data the authors concluded that the moderate glucose‐lowering effect of *S. cumini* was comparable with that of chlorpropamide (Kohli & Singh, 1993).

On the contrary, Teixeira et al. (1990, 2000) observed that the administration of a tea prepared by decoction of 2 g *S. cumini* dry leaves in 30 nondiabetic volunteers subjected to OGTT did not affect blood glucose levels (Teixeira et al., 1990, 2000). The absence of hypoglycemic action of this supplement was reported by the same authors in a clinical trial performed on T2DM patients that received a tea prepared from *S. cumini* dry leaves (2 g/day per liter of water) plus placebo tablets, placebo tea (prepared with *Imperata braziliensis* Trinius dried leaves) plus glyburide tablets (10 mg/day), or placebo tea plus placebo tablets for 28 days (Teixeira et al., 2004). This result was also confirmed by the authors in another study whose design was identical to that described above and in which included 27 T2DM patients (269). Based on these results the authors concluded that tea and extracts made from *S. cumini* leaves were ineffective on blood glucose levels (Teixeira et al., 2006).

However, Sahana et al. (2010) reported that in 15 newly diagnosed T2DM patients supplemented with 10 g/day of standardized *S. cumini* seed powder for 6 months was found a significant decrease of FPG at 3 months (−11.30 mg/dl, −7.40%, *p* = .016) and 6 months (−18.00 mg/dl, −11.80%, *p* = .043) as well as HOMA‐IR at 3 months (−3.80, −36.90%, *p* = .027) compared to baseline. However, the apparent reduction in PPG and HbA_1c_ levels at 6 months was not statistically significant (Sahana et al., 2010).

Another study evaluated the effect of *S. cumini* seed powder on glycemic control in 99 patients with T2DM not well controlled by diet and oral hypoglycemic agents that consumed 10 g/day of supplement or placebo for 90 days. It has been observed that *S. cumini* seed powder significantly decreased FPG and PPG levels after 30 days (−12.00 mg/dl, −8.40%, *p* = .0001 for FPG; −17.00 mg/dl, −7.00%, *p* = .085 for PPG), 60 days (−22.00 mg/dl, −15.40%, *p* = .0001 for FPG; −31.00 mg/dl, −12.80%, *p* = .0001 for PPG), and 90 days (−33.00 mg/dl, −23.10%, *p* = .0001 for FPG; −43.00 mg/dl, −17.80%, *p* = .0001 for PPG) as well as HbA_1c_ at the end of supplementation (−0.68%, −7.60%, *p* = .008) respect to baseline (Sidana et al., 2017).

### 3.14 *Ascophyllum nodosum* (L.) and *Fucus vesiculosus* (L.)

*A. nodosum* and *F. vesiculosus* are edible marine brown seaweeds used since ancient times by the coastal communities of Asia, Britain, and other countries (Rupérez, Ahrazem, & Leal, 2002). Recently it has been confirmed their efficacy in the treatment of metabolic diseases such as obesity and T2DM (Landin, Holm, Tengborn, & Smith, 1992; Ou, Kwok, Li, & Fu, 2001) due to the presence of numerous bioactive compounds including polyphenolics, phlrotannins, and fucoidans (Gabbia et al., 2017; S. Gupta & Abu‐Ghannam, 2011).

#### 3.14.1 Mechanisms of action

**FIGURE 14:**
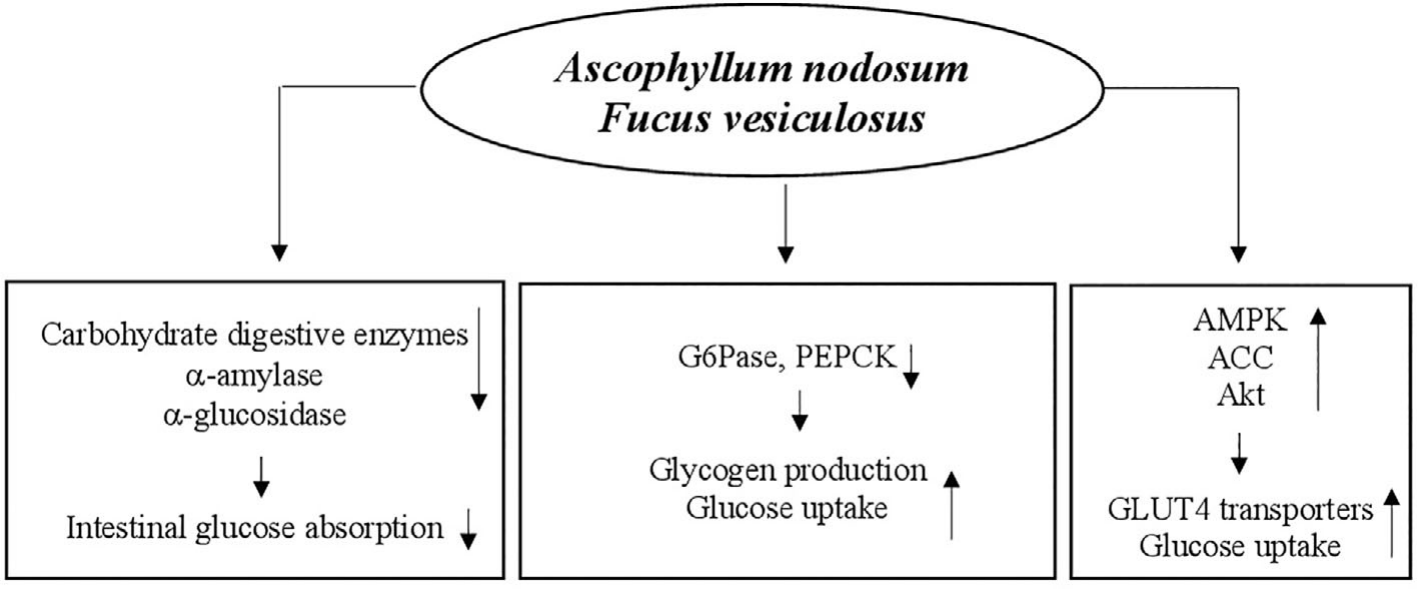
Hypoglycemic effects of *Ascophyllum nodosum* and *Fucus vesiculosus*. ACC, acetyl‐CoA carboxylase; Akt, serine/threonine protein kinase; AMPK, adenosine monophosphate‐activated protein kinase; GgPase, glucose‐6‐phosphatase; GLUT, glucose transporter; PEPCK, phosphoenolpyruvate carboxykinase

In vitro studies reported the potential hypoglycemic molecular mechanisms of bioactive compounds present in *A. nodosum* and *F. vesiculosus*. These molecules are able to reduce hyperglycemia via (a) inhibition of carbohydrate digestive enzymes, α‐amylase and α‐glucosidase thus delaying and decreasing intestinal absorption of glucose; (b) inhibition of hepatic enzymes (G6Pase and PEPCK) promoting glycogen production and glucose uptake at the cellular level; (c) upregulation of AMPK, ACC (acetyl‐CoA carboxylase) and Akt phosphorylation increasing the number of GLUT4 transporters at the cell membrane and glucose uptake at the cellular level (Murray, Dordevic, Ryan, & Bonham, 2019) (Figure 14).

#### 3.14.2 Clinical trials

Some human studies investigated the hypoglycemic effects of *A. nodosum* and *F. vesiculosus* (Table 14).

**TABLE 14:** Summary of human studies on hypoglycemic effects of *Ascophyllum nodosum* and *Fucus vesiculosus*

Subjects	Drug treatment and period	Results	References
Overweight or obesity patients (*n* = 12)	100 g of bread with *A. nodosum* 4% 4 hr following supplement intake	PPG no decrease	Hall, Fairclough, Mahadevan, and Paxman (2012)
Healthy subjects (*n* = 43)	2 capsules/day *A. nodosum* 900 mg and iodine 175 μg 6 months	FPG, FPI, and HOMA index no effect	Iacoviello et al. (2013)
Healthy subjects (*n* = 38)	500 mg *F. vesiculosus* 2 g *F. vesiculosus* 2 hr after carbohydrate consumption	PPG and PPI no effect Different insulin sensitivity in Asian subjects	Murray, Dordevic, Ryan, and Bonham (2018)
Normotensive subjects (*n* = 18)	2 g/day *F. vesiculosus* 3 hr after supplement ingestion	PPG has no effects except a reduction in females PPI iAUC and peak plasma insulin increase in Asian subjects	Murray et al. (2019)
Healthy subjects (*n* = 23)	500 mg *A. nodosum* and *F. vesiculosus* 3 hr after carbohydrate ingestion	No effect on glucose response Insulin iAUC decreases and Cederholm index of insulin sensitivity increases	Paradis, Couture, and Lamarche (2011)
Overweight or obesity patients (*n* = 50)	3 capsules/day *A. nodosum* 237.5 mg, *F. vesiculosus* 12.5 mg, and chromium picolinate 7.5 μg 6 months	FPG, FPI, and HOMA index decrease	De Martin, Gabbia, Carrara, and Ferri (2018)
Dysglycemic patients (*n* = 65)	3 capsules/day *A. nodosum* 237.5 mg, *F. vesiculosus* 12.5 mg, and chromium picolinate 7.5 μg 6 months	FPG, PPG, HbA_1c_, and HOMA index decrease	Derosa, Cicero, D'Angelo, and Maffioli, (2019)
T2DM patients (*n* = 175)	3 capsules/day *A. nodosum* 237.5 mg, *F. vesiculosus* 12.5 mg, and chromium picolinate 7.5 μg 6 months	FPG, PPG, and HbA_1c_ decrease	Derosa, Pascuzzo, D'Angelo, and Maffioli, (2019)

Abbreviations: FPG, fasting plasma glucose; FPI, fasting plasma insulin; HbA_1c_, glycated hemoglobin; HOMA index, homeostatic model assessment index; iAUC, incremental area under curve; PPG, post‐prandial plasma glucose; PPI, post‐prandial plasma insulin; T2DM, type 2 diabetes mellitus.

One trial evaluated the effect of *A. nodosum* on PPG in 12 overweight or obese men that received 100 g of bread with the supplement 4% at breakfast. It has been observed that *A. nodosum* did not affect PPG levels at a test meal 4 hr later (Hall et al., 2012).

Iacoviello et al. (2013) showed that the administration of 2 capsule/day each containing dried *A. nodosum* 900 mg and 175 μg iodine for 6 weeks in 43 healthy subjects did not affect FPG, FPI, and HOMA index respect to placebo (Iacoviello et al., 2013).

Murray et al. (2018) evaluated the effect of a powdered extract from *F. vesiculosus* on PPG and PPI in a sample of 38 healthy adults who received 500 mg and 2 g of the nutraceutical, containing 140 mg and 560 mg polyphenols, respectively, or 2 g of placebo on a single occasion prior to a carbohydrate load. Neither dose of *F. vesiculosus* altered PPG and PPI levels 2 hr after carbohydrate consumption compared to placebo. However, it has been observed that Asian participants had significantly higher insulin iAUC with respect to non‐Asian ones (*p* = .016) showing an influence of ethnicity on this parameter (Murray et al., 2018).

The same authors explored the efficacy of a powdered extract from *F. vesiculosus* on PPG and PPI in the evening in 18 normotensive subjects treated with 2 g of the nutraceutical, containing 560 mg polyphenols and 1,340 mg fucoidan, or placebo 30 min prior to a carbohydrate load. Three hours after the ingestion of the supplement no effect was seen on PPG and PPI with respect to placebo. However, *F. vesiculosus* reduced peak PPG in females (−9.01 mg/dl, −6.00%, *p* = .018) compared to placebo suggesting the influence of sex on this outcome. In addition, PPI iAUC and peak plasma insulin concentration were higher in the Asian population (*p* = 0.07 for both) with respect to non‐Asian ones indicating the influence of ethnicity (Murray et al., 2019).

Some trials evaluated the glucose‐lowering action of *A. nodosum and F. vesiculosus* in combination with each other and with other nutraceuticals.

A study conducted by Paradis et al. (2011) enrolled 23 healthy subjects who received an associate of *A. nodosum* and *F. vesiculosus* 500 mg or placebo 30 min prior to the ingestion of carbohydrates 50 g from bread. It has been observed that the supplement had no effect on glucose response 3 hr following the test meal consumption whereas produced a significant reduction of insulin iAUC (−12.10%, *p* = .04) and an increase of Cederholm index of insulin sensitivity (+7.90%, *p* < .05) respect to placebo (Paradis et al., 2011).

De Martin et al. (2018) explored the hypoglycemic effect of *A. nodosum*, *F. vesiculosus*, and chromium picolinate combination in 50 overweight or obese patients who consumed 3 capsules a day of the phytocomplex each containing *A. nodosum* 237.5 mg, *F. vesiculosus* 12.5 mg, and chromium picolinate 7.5 μg for a period of 6 months. The supplement significantly reduced FPG, FPI, and HOMA index after 3 months (−7.00 mg/dl, −6.40%, *p* < .05 for FPG; −3.80 μU/ml, −16.80%, *p* < .05 for FPI; −1.25, −20.40%, *p* < .05 for HOMA index) and 6 months of treatment (−12.00 mg/dl, −10.90%, *p* < .001 for FPG; −4.80 μU/ml, −21.20%, *p* < .05 for FPI; −1.68, −27.60%, *p* < .01 for HOMA index) respect to baseline values (De Martin et al., 2018).

Another trial investigated the effect of this nutraceutical combination on glycemic status in 65 dysglycemic patients randomized to three capsules a day of supplement or placebo for 6 months. At the end of treatment, the nutraceutical agent caused a significant decrease in FPG (−13.20 mg/dl, −11.20%, *p* < .05), PPG (−18.90 mg/dl, −11.80%, *p* < .05), HbA_1c_ (−5.00%, −9.30%, *p* < .05), and HOMA index (−0.60, −20.70%, *p* < .05) compared to placebo (Derosa, Cicero, D'Angelo, & Maffioli, 2019).

The same authors evaluated the efficacy of the nutraceutical combination on glucose parameters in another study whose design was identical to that described above and in which were enrolled 175 T2DM patients. The supplement produced a significant decrease in FPG, PPG, and HbA_1c_ respect to baseline (−35.60 mg/dl, −23.40%, *p* < .01 for FPG; −32.10 mg/dl, −17.00%, *p* < .01 for PPG; −0.80%, −10.80%, *p* < .05 for HbA_1c_) and to placebo (−25.60 mg/dl, −18.00%, *p* < .05 for FPG; −19.50 mg/dl, −11.10%, *p* < .05 for PPG; −0.40%, −5.70%, *p* < .05 for HbA_1c_) (Derosa, Pascuzzo, D'Angelo, & Maffioli, 2019).

### 3.15 *Ilex paraguariensis* (L) Pers

*I. paraguariensis* (St. Hill, Aquifoliaceae), also known as yerba mate, is a perennial tree that grows in southern Brazil, Paraguay, and Argentina. Dried and ground leaves of this plant are used to prepare drinks regionally known as chimarrão, tererê, or maté characterized by stimulating properties and bitter taste. Dried, ground, and roasted leaves of *I. paraguariensis* are used to prepare another beverage consumed in the form of infusion known as mate tea in Brazil. It is appreciated for its soft and pleasant aroma and is consumed hot or cold (Bracesco, Sanchez, Contreras, Menini, & Gugliucci, 2011). *I. paraguariensis* showed potential benefits to human health such as weight reduction, hypocholesterolemic and hypoglycemic effects as well as a high antioxidant activity due to the presence of various bioactive substances including polyphenols (chlorogenic and gallic acids, catechins), saponins, methylxantines (caffeine and theobromine), flavonoids, amino acids, minerals, and vitamins (Riachi & Bastos De Maria, 2017).

#### 3.15.1 Mechanisms of action

**FIGURE 15:**
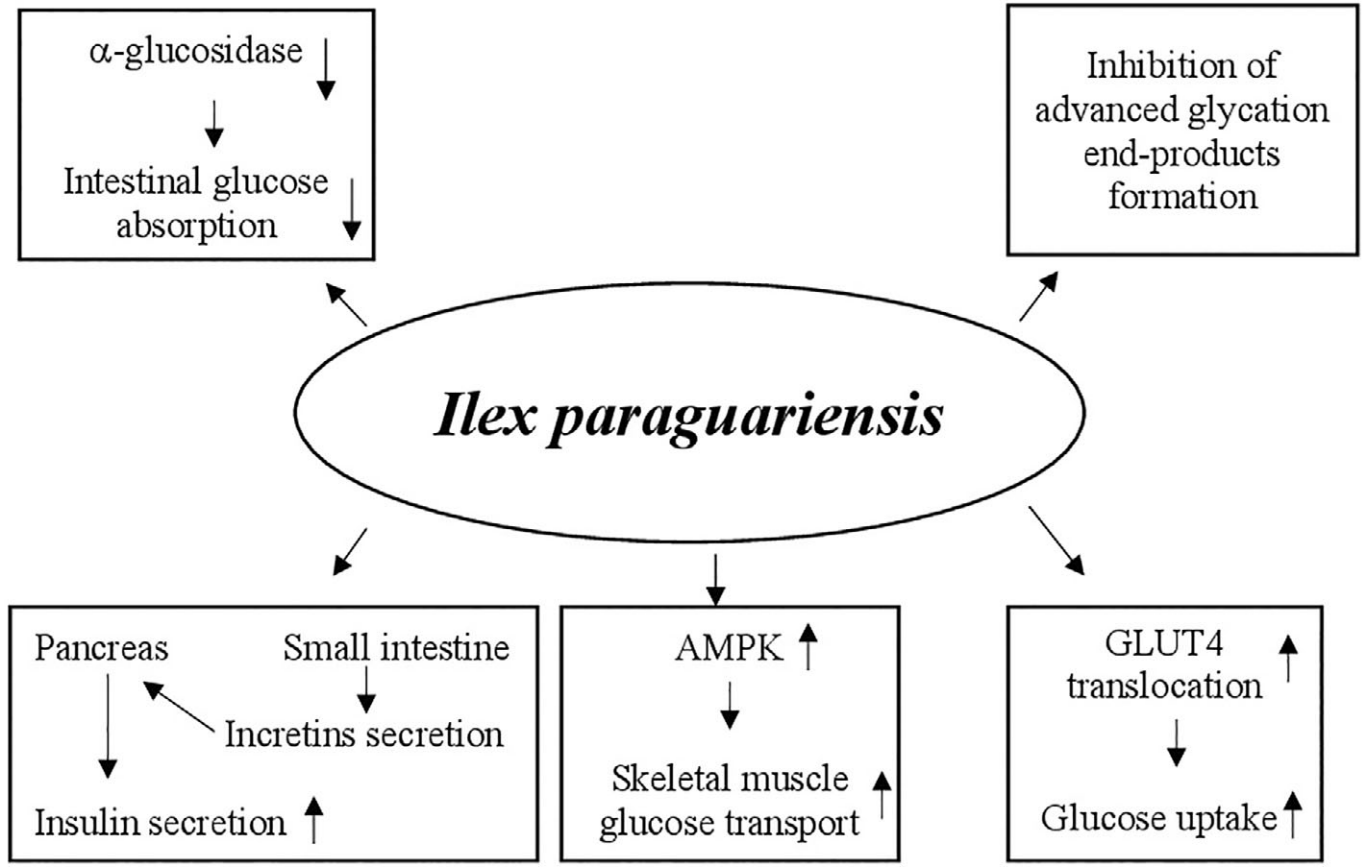
Hypoglycemic effects of *Ilex paraguariensis*. AMPK, adenosine monophosphate‐activated protein kinase; GLUT4, glucose transporter 4

Animal studies showed the hypothetical hypoglycemic mechanisms of bioactive substances, especially polyphenols, contained in *I. paraguariensis*. These compounds exert glucose‐lowering action (a) increasing insulin secretion both stimulating its release in the pancreas and inducing incretins secretion in the small intestine, which, in turn, promotes insulin release in the pancreas; (b) stimulating glucose transport in skeletal muscle via AMPK activation; (c) inducing GLUT4 translocation and glucose uptake activity; (d) inhibiting α‐glucosidase thus reducing intestinal glucose absorption; and (e) inhibiting advanced glycation end‐products formation (Ong, Hsu, & Tan, 2012; Pereira et al., 2012; Prasad, Anjana, Banerji, & Gopalakrishnapillai, 2010) (Figure 15).

#### 3.15.2 Clinical trials

Human studies reported contrasting evidence about the hypoglycemic effects of *I. paraguariensis* (Table 15).

**TABLE 15:** Summary of human studies on glucose‐lowering effects of *Ilex paraguariensis*

Subjects	Drug treatment and period	Results	References
Normolipidemic subjects (*n* = 42) Hyperlipidemic patients (*n* = 18)	200 ml matè tea (12.5 mg/ml) per day 2 months	FPG no decrease	Arcari et al. (2011)
T2DM patients (*n* = 29) Prediabetic patients (*n* = 29)	19.8 g yerba mate leaves to 990 of boiling water per day 60 days	FPG and HbA_1c_ decrease in T2DM patients	Klein et al. (2011)
Overweight patients (*n* = 46)	334 mg/day green mate powder extract 6 weeks	FPG no decrease	H. J. Kim, Ko, et al. (2012)
T2DM patients (*n* = 11) Prediabetic patients (*n* = 11)	19.8 g yerba mate leaves to 990 of boiling water per day 60 days	FPG and HbA_1c_ decrease in T2DM patients; HbA_1c_ decrease in prediabetics	Boaventura et al. (2013)
Obese women (*n* = 33)	3 g/day matè extract 6 weeks	FPG no decrease	Jung and Hur (2016)
IFG, IGT patients (*n* = 137)	1 tablet/day *I. paraguariensis* 500 mg, white mulberry 50 mg, and chromium picolinate 100 μg 3 months	FPG and HOMA index decrease M value increase	Derosa, D'Angelo, and Maffioli (2020)
IFG, IGT patients (*n* = 143)	1 tablet/day *I. paraguariensis* 1,000 mg, white mulberry 50 mg, and chromium picolinate 100 μg 3 months	FPG, PPG, HbA_1c_, and HOMA index decrease M value increase	Derosa, D'Angelo, and Maffioli (2021)

Abbreviations: FPG, fasting plasma glucose; HbA_1c_, glycated hemoglobin; HOMA index, homeostatic model assessment index; IFG, impaired fasting glucose; IGT, impaired glucose tolerance; PPG, post‐prandial plasma glucose; T2DM, type 2 diabetes mellitus.

One trial showed that the ingestion of 200 ml of matè tea (12.5 mg/ml) per day for 2 months in 42 normolipidemics and 18 hyperlipidemics did not affect FPG levels in either group (Arcari et al., 2011).

Conversely, in the study conducted by Klein et al. (2011) 29 T2DM and 29 prediabetic patients consumed roasted matè tea with or without nutritional counseling or only undertook dietetic counseling for 60 days. Matè tea was prepared by adding 6.6 g of yerba mate leaves to 330 ml of boiling water and ingested thrice a day, at breakfast, lunch, and dinner. It has been observed that only T2DM patients exhibited a significant decrease in FPG after 60 days (−25.00 mg/dl, −17.00%, *p* < .05) and HbA_1c_ after 20 days (−0.80%, −10.50%, *p* < .05) and 60 days (−0.70%, −9.30%, *p* < .05) compared with baseline values following matè tea consumption (Klein et al., 2011).

Instead, H. J. Kim, Ko, Storni, Song, & Cho (2012) evaluated the effect of a powdered extract from dried green leaves of *I. paraguariensis* on FPG in 46 overweight subjects that received 334 mg/day of supplement or placebo for 6 weeks. At the end of treatment, no significant changes were found in the FPG levels of both groups (H. J. Kim, Ko, et al., 2012).

However, another study showed results partially in agreement with those of Klein et al. (2011). A sample of 11 T2DM and 11 prediabetic patients ingested 330 ml of roasted matè tea three times a day, at breakfast, lunch, and dinner for 60 days. The matè tea was prepared as described in the study by Klein et al. (2011) In T2DM patients the supplement significantly decreased FPG (−10.89 mg/dl, −7.40%, *p* < .05) and HbA_1c_ (−1.15%, −15.00%, *p* < .05) after 60 days respect to baseline, whereas in prediabetics it reduced HbA_1c_ (−0.33%, −5.40%, *p* < .05) after 40 days compared to baseline (Boaventura et al., 2013).

On the contrary, Jung and Hur demonstrated that the administration of matè extract 3 g/day or placebo in 33 obese women for 6 weeks did not modify FPG levels after supplementation (Jung & Hur, 2016).

Some trials explored the hypoglycemic action of *I. paraguariensis* in association with other supplements.

Derosa, D'Angelo, and Maffioli (2020) evaluated the effects of a nutraceutical compound containing *I. paraguariensis* 500 mg, white mulberry 50 mg of which 1 mg I‐deoxinojirimcina and chromium picolinate 100 μg on glucose parameters in 137 patients with IFG or IGT randomized to take 1 tablet at breakfast of supplement or placebo for 3 months. The nutraceutical combination significantly reduced FPG and HOMA index both respect to baseline (−8.70 mg/dl, −7.80%, *p* < .05 for FPG; −0.19, −7.90%, *p* < .05 for HOMA index) and to placebo (−11.10 mg/dl, −9.80%, *p* < .05 for FPG; −0.24, −9.80%, *p* < .05 for HOMA index). In addition, M value resulted higher compared to baseline and placebo (*p* < .05 for both) after supplementation. It has been also observed that 67% of patients returned to normal insulin sensitivity and 33% of them had an increase of M value following nutraceutical assumption respect to placebo (Derosa, D'Angelo, & Maffioli, 2020).

The same authors investigated the efficacy of another nutraceutical containing *I. paraguariensis* 1,000 mg, white mulberry 50 mg of which 1 mg I‐deoxinojirimcina and chromium picolinate 100 μg on glycemic status in 143 patients with IFG or IGT treated with a supplement or placebo 1 tablet at breakfast for a period of 3 months. It has been found a significant decrease of FPG, PPG, HbA_1c_, and HOMA index compared to baseline (−12.00 mg/dl, −10.50%, *p* < .05 for FPG; −12.50 mg/dl, −9.40%, *p* < .05 for PPG; −0.40%, −6.80%, *p* < .05 for HbA_1c_; −0.15, −5.90%, *p* < .05 for HOMA index) and to placebo (−11.70 mg/dl, −13.50%, *p* < .05 for FPG; −11.70 mg/dl, −8.90%, *p* < .05 for PPG; −0.30%, −5.20%, *p* < .05 for HbA_1c_; −0.20, −7.80%, *p* < .05 for HOMA index) after supplementation. Moreover, the M value was higher compared to baseline (*p* < .05) following nutraceutical treatment. It has been also reported that 78% of patients returned to have a M value whitin range of normal insulin sensitivity and 22% of them had an amelioration of M value with the supplement compared to placebo (Derosa et al., 2021).

### 3.16 Morus

The genus *Morus*, also known as mulberry, belongs to the family Moraceae. The most common species are *Morus alba* (L) Pers (white mulberry), *Morus nigra* (L) Pers (blackberry), and *Morus rubra* (L) Pers (red berry) largely distributed in India, China, Japan, North Africa, Arabia, and southern Europe (V. Kumar & Chauhan, 2008).

The species *Morus* contain a great amount of phenolic compounds such as flavonoids and anthocyanins and are used for the prevention of liver and kidney disorders, joint damage, and antiaging thanks to the antioxidant properties of these substances (Mena et al., 2016). Moreover, the species *Morus* are efficacy in the treatment of T2DM due to the wide presence of sugar‐mimicking alkaloids known to have hypoglycemic properties, including 1,4‐dideoxy‐1,4‐imino‐d‐arabinitol, 1‐deoxynojirimycin (DNJ), and 1,4‐dideoxy‐1,4‐imino‐d‐ribitol (Konno et al., 2006; Sánchez‐Salcedo, Amorós, Hernández, & Martínez, 2017). Generally, fruits, roots, and leaves are the parts of the mulberry tree studied to test their therapeutic purposes (Rodrigues et al., 2019).

#### 3.16.1 Mechanisms of action

**FIGURE 16:**
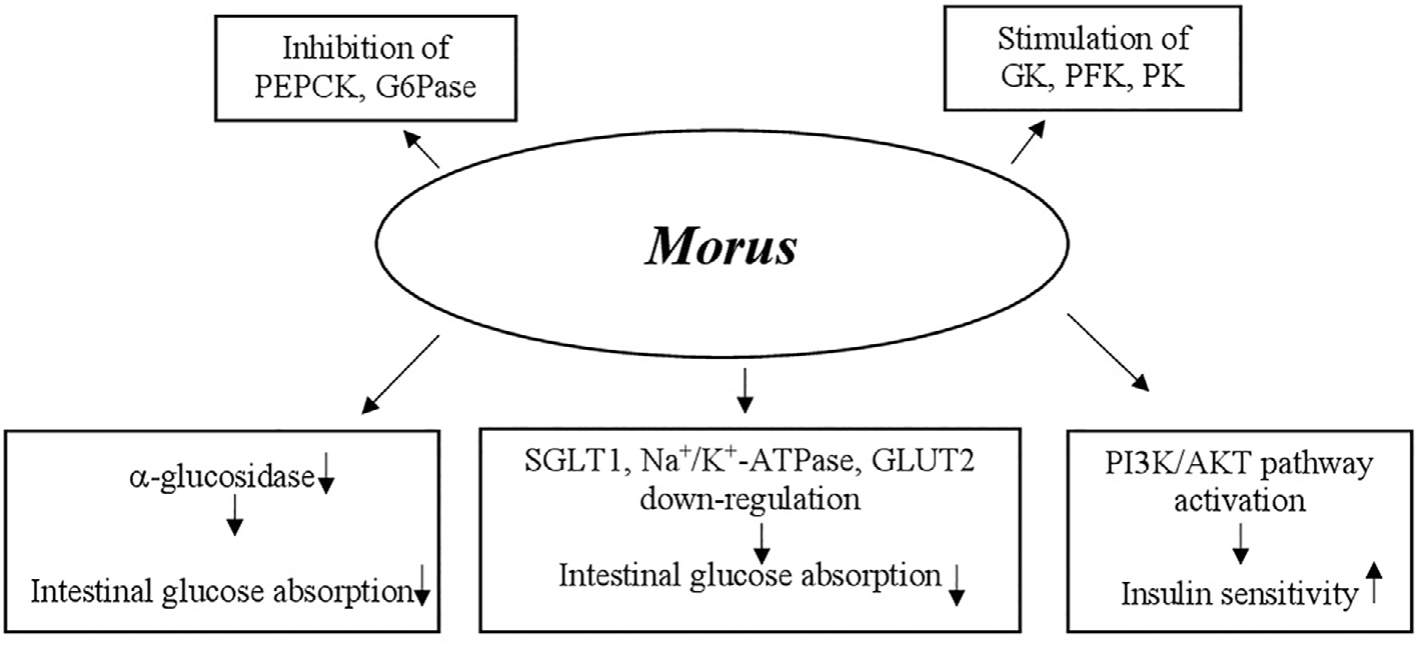
Hypoglycemic effects of *Morus*. G6Pase, glucose‐6‐phosphatase; GK, glucokinase; GLUT2, glucose transporter 2; PEPCK, phosphoenolpyruvate carboxykinase; PFK, phosphofructokinase; PI3K/AKT, phosphatidylinositol‐3‐kinase/protein kinase B; PK, pyruvate kinase; SGLT1, intestinal sodium glucose co‐transporter 1

The potential mechanisms that underlie the hypoglycemic activity of species *Morus* consist of (a) decrease of intestinal glucose absorption by inhibition of α‐glucosidase activity and down‐regulation of intestinal sodium‐glucose co‐transporter 1 (SGLT1), Na^+^/K^+^‐ATPase as well as GLUT2 mRNA and protein expression; (b) enhanced of insulin sensitivity by activation of insulin signaling PI3K/AKT pathway; and (c) suppression of gluconeogenic enzymes (PEPCK and G6Pase) and stimulation of glycolytic enzymes activities (glucokinase [GK], phosphofructokinase [PFK], and pyruvate kinase [PK]) (Kimura et al., 2007; Wang, Shen, Zhao, & Ye, 2021) (Figure 16).

#### 3.16.2 Clinical trials

Clinical trials reported in Literature showed controversial evidence about the effect of *Morus* on glycemic control (Table 16).

**TABLE 16:** Summary of human studies on hypoglycemic effects of *Morus*

Subjects	Drug treatment and period	Results	References
Healthy subjects (*n* = 24)	*Morus alba* powder leaves enriched with 0.4, 0.8, or 1.2 g DNJ 3 hr 2 *M. alba* powder leaves enriched with 3.6 g/day DNJ 3 38 days	PPG and PPI decrease FPG no decrease	Kimura et al. (2007)
T2DM patients (*n* = 10) Controls (*n* = 10)	*M. alba* leaf extract 1 g/day 1 week	PPG decrease	Mudra, Ercan‐Fang, Zhong, Furne, and Levitt (2007)
Dysglycemic patients (*n* = 12) Dysglycemic patients (*n* = 76)	*M. alba* leaf extract enriched with 3, 6, or 9 mg DNJ 2 hr 2 *M. alba* leaf extract enriched with 18 g/day DNJ 12 weeks	PPG and PPI decrease FPG, FPI, HbA_1c_, and GA no decrease; 1,5AG decrease	Asai et al. (2011)
T2DM patients (*n* = 10) Healthy subjects (*n* = 10)	3.3 g *M. alba* leaf extract 2 hr	PPG and PPI decrease	Nakamura, Hashiguchi, Yoshihiko, and Oku (2011)
Healthy subjects (*n* = 50)	1.25, 2.5, or 5 g *M. alba* leaf aqueous extract 3 hr	PPG decrease	Chung, Kim, Kim, and Kwon (2013)
IFG patients (*n* = 38)	5 g/day *M. alba* leaf aqueous extract 4 weeks	PPG, PPI, insulin AUC and C‐peptide decrease	J. Y. Kim et al., 2015
T2DM patients (*n* = 48)	70 ml *M. alba* leaf tea 1.30 hr	FPG and PPG decrease	Banu et al., 2015
Non‐diabetic subjects (*n* = 14)	2 g *M. alba* leaf tea powder 2.30 hr	PPG decrease	Sukriket, Lookhanumarnjao, and Bumrungpert (2016)
T2DM patients (*n* = 17)	3 g/day *M. alba* leaf extract 3 months	Post‐prandial SMBG and HbA_1c_ decrease	Riche, Riche, East, Barrett, and May (2017)
Healthy subjects (*n* = 85) Obese (*n* = 54)	*M. alba* leaf powder with DNJ 6, 12, and 18 mg 3 hr 2 *M. alba* leaf powder with DNJ 36 mg/day 12 weeks	PPG decrease FPG and HbA_1c_ decrease	Thaipitakwong, Supasyndh, Rasmi, and Aramwit (2020)
Healthy subjects (*n* = 38)	250 mg *M. alba* leaf aqueous extract 2 hr	PPG, PPI, glucose, and insulin iAUC decrease	Thondre, Lightowler, Ahlstrom, and Gallagher (2021)
T2DM patients (*n* = 100)	9 ml/day 4% hydro‐alcoholic extract of *Morus nigra* leaves 3 months	FPG and HbA_1c_ decrease	Momeni, Salehi, Absalan, and Akbari (2021)
IGT and mild T2DM patients (*n* = 62)	6 g/day white mulberry leaf water extract powder, Korean red ginseng powder, and banaba leaf water extract powder 24 weeks	OGTT AUC glucose decrease; FPG FPI, HOMA‐IR, and HbA_1c_ no decrease	H. J. Kim, Yoon, et al. (2012)
Dyslipidemia patients (*n* = 40)	8 capsules/day each containing *Crataegus pinnatifida* 129 mg, *Alisma orientalis* 86 mg, *Stigma maydis* 86 mg, *Ganoderma lucidum* 43 mg, *Polygonum multiflorum* 43 mg, and *M. alba* 43 mg 12 weeks	HbA_1c_ decrease	Hu, Zeng, and Tomlinson (2014)
Hypercholesterolemia patients (*n* = 23)	Combination A: Policosanol (10 mg), red yeast rice (200 mg; 3 mg monacolin K), Berberine (500 mg), Astaxantine (0.5 mg), folic acid (200 μg), and coenzyme Q10 (2 mg) Combination B: Berberine (531.25 mg), red yeast rice powder (220 mg; 3.3 mg monacolin K), and leaf extract of *M. alba* (200 mg) 8 weeks	FPG FPI, HbA_1c_, and HOMA index decrease with combination B	Trimarco et al. (2015)
IFG, IGT patients (*n* = 137)	1 tablet/day white mulberry 50 mg, *Ilex paraguariensis* 500 mg, and chromium picolinate 100 μg 3 months	FPG and HOMA index decrease M value increase	Derosa, D'Angelo, Vanelli, and Maffioli (2020); Derosa, D'Angelo, and Maffioli (2020)
IFG, IGT patients (*n* = 143)	1 tablet/day white mulberry 50 mg, *I. paraguariensis* 1,000 mg, and chromium picolinate 100 μg 3 months	FPG, PPG, HbA_1c_, and HOMA index decrease M value increase	Derosa et al. (2021)

Abbreviations: 1,5AG, 1,5‐anhydroglucitol; FPG, fasting plasma glucose; FPI, fasting plasma insulin; GA, glycated albumin; HbA_1c_, glycated hemoglobin; HOMA‐IR, homeostatic model assessment of insulin resistance; iAUC, incremental area under the curve; IFG, impaired fasting glucose; IGT, impaired glucose tolerance; OGTT, oral glucose tolerance test; PPG, post‐prandial plasma glucose; PPI, post‐prandial plasma insulin; SMBG, self‐monitoring blood glucose; T2DM, type 2 diabetes mellitus.

In the study conducted by Kimura et al. (2007), 24 healthy subjects received mulberry (*M. alba*) powder leaves enriched with 0.4, 0.8, or 1.2 g of DNJ followed by an oral sucrose load. It has been observed that the supplement contained 0.8 or 1.2 g of DNJ significantly decrease PPG and PPI levels (*p* < .05 for both) at 60 min after sucrose load with respect to placebo. The authors also reported that the administration of 3.6 g/day of DNJ‐enriched mulberry powder leaves or placebo in 24 healthy individuals for 38 days did not change FPG levels after supplementation (Kimura et al., 2007).

Another trial evaluated the efficacy of mulberry (*M. alba*) leaf extract on PPG in 10 controls and 10 T2DM patients who ingested 75 g of sucrose with 1 g of supplement or placebo for 1 week. The supplement caused a significant decrease of PPG over the first 120 min of the study both in controls (*p* = .005) and diabetics (*p* = .002) compared to placebo. In addition, the blood glucose reduction at the tail end of the study was less with nutraceutical respect to placebo (Mudra et al., 2007).

Asai et al. (2011) investigated the effects of a single intake of mulberry (*M. alba*) leaf extract enriched with 3, 6, or 9 mg DNJ or placebo on PPG and PPI in 12 dysglycemic subjects after a carbohydrate (200 g boiled white rice) tolerance test. The supplement tended to reduce PPG levels in a dose‐dependent manner. There was a significant decrease in PPG values at 30 min after the test meal in the groups who have received mulberry leaf extract enriched with 6 mg (−24.00 mg/dl, −16.60%, *p* < .05) and 9 mg DNJ (−31.00 mg/dl, −21.40%, *p* < .05) compared to placebo. Post‐prandial insulin levels were found significantly lower at 30 min in the subjects who were given the supplement with 3 mg (−4.70 μU/ml, −29.70%, *p* < .05), 6 mg (−6.60 μU/ml, −41.80%, *p* < .05), and 9 mg DNJ (−5.80 μU/ml, −36.70%, *p* < .05) than placebo. It has been also observed that the ingestion of 18 g/day of DNJ‐enriched mulberry leaf extract or placebo in 76 dysglycemic subjects for 12 weeks did not modify FPG, FPI, HbA_1c_, and glycated albumin (GA) after supplementation. However, the nutraceutical increased serum 1,5‐anhydroglucitol (1,5AG), an indicator of post‐prandial glycemic control, at 4 (+2.10 μg/ml, +12.00%, *p* < .05), 8 (+3.50 μg/ml, +20.00%, *p* < .05), and 12 weeks (+3.00 μg/ml, +17.10%, *p* < .05) respect to baseline as well as at 8 (+3.30 μg/ml, +18.60%, *p* < .05) and 12 weeks (+3.00 μg/ml, +17.10%, *p* < .05) compared to placebo (Asai et al., 2011).

One trial explored the efficacy of a jelly containing 3.3 g of *M. alba* leaf extract (LEM) on PPG and PPI in 10 T2DM patients treated with or without sulfonylureas and 10 healthy subjects. Type 2 diabetics exhibited a significant decrease in PPG (−61.00 mg/dl, −29.20%, *p* < .05) and PPI (−250.00 pg/ml, −31.20%, *p* < .05) at 30 min after supplement ingestion compared to placebo. A similar trend for PPG and PPI (*p* < .05 for both) was observed in T2DM patients untreated or treated with sulfonylureas. Also, in healthy subjects, there was a significant reduction in PPG (−28.00 mg/dl, −22.40%, *p* < .05) and PPI (−1,100.00 pg/ml, −64.70%, *p* < .05) at 30 min after LEM intake respect to placebo (Nakamura et al., 2011).

In the study of Chung et al. (2013), 50 healthy subjects took a beverage containing 75 g maltose powder dissolved in water and mixed with 0 (placebo) 1.25, 2.5, or 5 g of mulberry (*M. alba*) leaf aqueous extract (MLAE). It has been shown that PPG significantly decreased after ingestion of 2.5 g MLAE at 30 min (−38.00 mg/dl, −41.39%, *p* = .0137) and 5 g MLAE at 60 min (−44.00 mg/dl, −47.80%, *p* = .0423) following the maltose load (Chung et al., 2013).

A randomized, double‐blind placebo‐controlled trial assessed the effect of MLAE on PPG, PPI, and post‐prandial C‐peptide in 38 IFG patients given 5 g/day of supplement. After 4 weeks of treatment, the nutraceutical determined a significant decrease in PPG, PPI, and post‐prandial C‐peptide 30 min (*p* = .0003 for PPG; *p* = .0005 for PPI; *p* = .0096 for post‐prandial C‐peptide) and 60 min (*p* = .0325 for PPG; *p* = .035 for PPI; *p* = .0156 for post‐prandial C‐peptide) after a carbohydrate load compared to placebo. There was also a reduction of AUC for PPI (−26.22 μIU/ml × hr, −36.13%, *p* = .0207) and post‐prandial C‐peptide (−1.04 ng/ml × hr, −17.51%, *p* = .059) after supplementation respect to placebo (J. Y. Kim et al., 2015).

One study reported that the administration of mulberry (*M. alba*) leaf tea or normal black tea 70 ml together with a standardized breakfast in 48 T2DM patients produced a significant reduction of FPG (−25.05 mg/dl, −14.03%, *p* = .055) and PPG at 90 min (−76.99 mg/dl, −26.81%, *p* < .001) after the mulberry tea consumption respect to the normal one (Banu et al., 2015).

Sukriket et al. (2016) showed that in 14 non‐diabetic subjects who drank mulberry (*M. alba*) leaf tea (2 g of mulberry leaf tea powder) or water as control 30‐min prior OGTT, the supplement significantly decreases PPG at 30 min (−25.65 mg/dl, −16.67%, *p* = .04) compared to control (Sukriket et al., 2016).

It has also been evaluated the effect of mulberry (*M. alba*) leaf extract (MLE) on HbA_1c_ and self‐monitoring blood glucose (SMBG) in 17 T2DM patients who took 3 g/day of supplement or placebo for 3 months. At the end of treatment, post‐prandial SMBG was significantly reduced in the MLE group both to baseline (−16.10%, *p* < .05) and to placebo (−18.20%, *p* < .05) whereas HbA_1c_ was decreased without reaching statistical significance (−0.36%, −4.90%, *p* = .079) after supplementation respect to baseline (Riche et al., 2017).

Another trial firstly examined the optimal dose of DNJ in mulberry (*M. alba*) leaves for attenuating PPG levels in 85 healthy adults randomly allocated to intake a single dose of 50 g sucrose mixed with mulberry leaf powder 0 (control), 2.3, 4.6, and 6.9 g corresponding to 0, 6, 12, and 18 mg of DNJ. It has been observed that DNJ 6 mg significantly reduced PPG at 30 min after carbohydrate load (−23.60 mg/dl, −16.86%, *p* < .001) compared with control. Similarly, the doses of DNJ 12 and 18 mg decreased PPG at 30 (−34.60 mg/dl, −24.71%, *p* < .001 for DNJ 12 mg; −41.80 mg/dl, −29.86%, *p* < .001 for DNJ 18 mg) and 60 min (−16.40 mg/dl, −13.67%, *p* < .05 for DNJ 12 mg; −21.00 mg/dl, −17.50%, *p* < .001 for DNJ 18 mg) respect to control and at 30 min (−11.00 mg/dl, −9.45%, *p* < .05 for DNJ 12 mg; −18.20 mg/dl, −15.64%, *p* < .001 for DNJ 18 mg) compared to DNJ 6 mg. These findings showed that the dose of DNJ 18 mg reduced PPG levels to a greater extent than the others. However, DNJ 12 mg resulted in the optimal dose attenuating PPG levels due to a minor incidence rate of gastrointestinal side effects respect to DNJ 18 mg. Subsequently, the same authors recruited 54 obese patients with FPG levels of 100–140 mg/dl and or 2 hr‐PPG of 140–199 mg/dl who received 12 mg of mulberry DNJ three times daily before main meals or nutritional counseling. After 12 weeks of treatment, the supplement caused a significant decrease in FPG (−4.22 mg/dl, −3.93%, *p* < .05) and HbA_1c_ (−0.12%, −2.06%, *p* < .05) compared with baseline values (Thaipitakwong et al., 2020).

Thondre et al. (2021) explored the efficacy of MLAE standardized to contain 5% DNJ on PPG and PPI in 38 healthy individuals who were given 75 g sucrose alone (placebo) or mixed with the supplement 250 mg. It has been found a significant decrease in PPG at 15 (*p* < .001), 30 (*p* < .001), 45 (*p* = .008), and 120 min (*p* < .001) as well as in PPI at 15 (*p* < .001), 30 (*p* < .001), 45 (*p* < .001), 60 (*p* = .001), and 120 min (*p* < .001) following supplementation compared to placebo. The supplement also significantly reduced the mean glucose and insulin iAUC at 60 (−45%, *p* < .001 for glucose iAUC; −49%, *p* < .001 for insulin iAUC), 90 (−44%, *p* < .001 for glucose iAUC; −46% *p* < .001 for insulin iAUC) and 120 min (−42%, *p* = .001 for glucose iAUC; −40%, *p* < .001 for insulin iAUC), the peak blood glucose (−40%, *p* < .001) and the peak insulin (−41%, *p* < .001) compar to placebo (Thondre et al., 2021).

In the study of Momeni et al. (2021) 100 T2DM patients were treated with 3 ml of 4% hydroalcoholic extract of *M. nigra* leaves or placebo in water, thrice a day, for 3 months. It has been observed that the supplement significantly decreased FPG (−21.00 mg/dl, −11.52%, *p* < .001) and HbA_1c_ (−1.10%, −15.21%, *p* < .001) compared to baseline values (Momeni et al., 2021).

It has been also evaluated the hypoglycemic action of Morus in association with other nutraceuticals.

One study showed the efficacy of mulberry leaf extract, Korean red ginseng powder and banaba leaf extract association on glycemic parameters as already reported (H. J. Kim, Yoon, et al., 2012).

Another trial evaluated the effect of a nutraceutical combination in the form of capsules each containing *Crataegus pinnatifida* 129 mg, *Alisma orientalis* 86 mg, *Stigma maydis* 86 mg, *Ganoderma lucidum* 43 mg, *Polygonum multiflorum* 43 mg, and *M. alba* 43 mg on glucose parameters in 40 dyslipidemic patients who received the supplement 8 capsules/day or placebo for 12 weeks. At the end of treatment, it has been observed a significant decrease in HbA_1c_ after supplementation (−0.28%, −4.25%, *p* < .01) compared to baseline (Hu et al., 2014).

In the study of Trimarco et al. (2015), 23 hypercholesterolemic patients not on statins received two different nutraceutical combinations (A and B) for 8 weeks. Combination A was composed of policosanol (10 mg), red yeast rice (200 mg; 3 mg monacolin K), berberine (500 mg), astaxantine (0.5 mg), folic acid (200 μg), and coenzyme Q10 (2 mg) whereas combination B contained berberine (531.25 mg), red yeast rice powder (220 mg; 3.3 mg monacolin K) and leaf extract of *M. alba* (200 mg). It has been observed that combination B significantly reduced FPG, FPI, HbA_1c_ and HOMA index both to baseline (−7.65 mg/dl, −8.32%, *p* = .0001 for FPG; −2.20 μU/ml, −21.78%, *p* = .006 for FPI; −0.11%, −2.10%, *p* = .002 for HbA_1c_; −0.67, −28.76%, *p* = .006 for HOMA index) and to combination A (−9.30 mg/dl, −9.93%, *p* = .0001 for FPG; −2.30 μU/ml, −22.55%, *p* = .02 for FPI; −0.09%, −1.70%, *p* = .03 for HbA_1c_; −0.79, −32.24%, *p* = .002 for HOMA index) (Trimarco et al., 2015).

In addition, it has been examined the effect of white mulberry, *I. paraguariensis* and chromium picolinate combined on glucose metabolism as previously described (Derosa, D'Angelo, & Maffioli, 2020; Derosa, D'Angelo, Vanelli, & Maffioli, 2020; Derosa et al., 2021).

## 4 CONCLUSIONS

The findings suggest a role for some nutraceuticals, and in particular *Berberis*, *Banaba*, *Curcumin*, and *Guar gum*, in the management of prediabetes and diabetes; these nutraceuticals could be proposed as an additional tool to ameliorate the glycemic profile in prediabetes and T2DM supporting their therapeutic use also due to their tolerability and safe on the basis of the analyzed trials on the efficacy of the supplements on glucose homeostasis. However, different studies presented conflicting results as regards the efficacy of some supplements such as *Morus*, *I. paraguariensis*, Omega‐3, *A. cepa*, and *T. faenum graecum* on glucose metabolism. The controversial data may be mainly due to unstandardized preparations, a wide range of supplements doses used, variability of the trial duration, studies not well designed, small sample size, and differences in population characteristics. Because of this, long‐term clinical trials are needed to confirm their glucose‐lowering effects. Further studies are also required for *E. jambolana*, as well as for *A. nodosum* and *F. vesiculosus* which did show hypoglycemic effects when given in combination, but not when taken separately. Further trials are also needed for quercetin.

## CONFLICT OF INTEREST

The authors have no relevant affiliations or financial involvement with any organization or entity with a financial interest in or financial conflict with the subject matter or materials discussed in the manuscript. This includes employment, consultancies, honoraria, stock ownership or options, expert testimony, grants or patents received or pending, or royalties. No writing assistance was utilized in the production of this manuscript.

## Data Availability

Data sharing not applicable to this article as no datasets were generated or analysed during the current study.
